# Supplementary material for: Effects of Hypoxia on RNA Cargo in Extracellular Vesicles from Human Adipose-Derived Stromal/Stem Cells
Source: Int J Mol Sci. 2022 Jul 2;23(13):7384. doi: 10.3390/ijms23137384 (PMC9266528; doi:10.3390/ijms23137384)
Supplement: Supplementary file 1 [file ijms-23-07384-s001.zip › Suppl. Table. S1.pdf]

Supplement

Table S1. Primer used for PCR analyses.

Gene	Primer Forward	Primer Reverse	Product Length (bp)	NCBI Reference Sequence
IL-10	TAC CTG GAG GAG GTG AT	GGC CTT GCT CTT GTT TTC AC	148	NM_000572.3
IL-6	AAA GAT GGC TGA AAA AGA TGG ATG C	ACA GCT CTG GCT TGT TCC TCA CTA C	150	NM_000600.4
IDO	GGG CTT TGC TCT GCC AAA TC	CAA CTC TTT CTC GAA GCT GG C	116	NM_002164.6
TNFa	CGG GAC GTG AGC TGG CCG AGG AG	CAC CAG CTG GTT ATC TCT CAG CTC	354	NM_000594.4
HGF	TTC CAT GAT ACC ACA CGA ACA	TGG ATT GCT TGT GAA ACA CC	124	NM_000601.6
β-Actin	ACT GGA ACG GTG AAG GGT GAC	AGA GAA GTG GGG TGG CTT TT	169	NM_001101
