# Supplementary material for: Effects of Hypoxia on RNA Cargo in Extracellular Vesicles from Human Adipose-Derived Stromal/Stem Cells
Source: Int J Mol Sci. 2022 Jul 2;23(13):7384. doi: 10.3390/ijms23137384 (PMC9266528; doi:10.3390/ijms23137384)
Supplement: Supplementary file 1 [file ijms-23-07384-s001.zip › Suppl. Table. S2.pdf]

Table S2. List of all significantly regulated transcripts.

ENST	Symbol	log2FC (hEVs/nEVs)	padj (hEVs/nEVs)
ENST00000341049	CAV1	30.00	1.2172E-10
ENST00000530019	FTH1	30.00	1.2172E-10
ENST00000510187	SQSTM1	29.63	1.2172E-10
ENST00000262030	ATP5F1B	29.55	1.2172E-10
ENST00000295955	RPL9	29.48	1.2172E-10
ENST00000354420	RNH1	29.44	1.2172E-10
ENST00000407332	CYB5R3	29.42	1.2172E-10
ENST00000236671	CTSD	29.37	1.2172E-10
ENST00000358278	TPM1	29.36	1.5481E-11
ENST00000439696	ZFP36L1	29.35	1.2172E-10
ENST00000451791	PENK	29.34	1.2172E-10
ENST00000620463	None	29.27	1.2172E-10
ENST00000643579	TPM4	29.22	1.2172E-10
ENST00000467076	FDPS	29.21	1.2172E-10
ENST00000434316	TMCO3	29.20	1.2172E-10
ENST00000433215	SPG21	29.20	1.2172E-10
ENST00000393467	CAV1	29.12	1.2172E-10
ENST00000337554	TSPO	29.07	1.2172E-10
ENST00000451728	CNBP	29.02	1.2172E-10
ENST00000341872	LGALS8	29.01	1.2172E-10
ENST00000294072	CYB561A3	28.97	1.2172E-10
ENST00000602162	ZNF611	28.97	1.2172E-10
ENST00000340828	CCNG1	28.96	1.2172E-10
ENST00000348547	ERGIC3	28.94	1.2172E-10
ENST00000619920	PSMC3	28.92	1.2172E-10
ENST00000368209	HDGF	28.89	1.2172E-10
ENST00000646241	CTSA	28.87	1.2172E-10
ENST00000262139	WIPI1	28.76	1.2172E-10
ENST00000341223	CFL2	28.75	1.2172E-10
ENST00000450814	TKT	28.75	1.2172E-10
ENST00000219204	ARL2BP	28.70	1.2172E-10
ENST00000583389	EIF4A1	28.70	1.2172E-10
ENST00000369349	PDE4DIP	28.67	1.2172E-10
ENST00000329582	IL20RB	28.66	1.2172E-10
ENST00000361234	RNF34	28.62	1.2172E-10
ENST00000312263	DCAF6	28.61	1.2172E-10
ENST00000395648	TP53I11	28.60	1.2172E-10
ENST00000248879	DGCR6L	28.58	1.2172E-10
ENST00000304786	SRP9	28.56	1.2172E-10
ENST00000258770	TBRG4	28.56	1.2172E-10
ENST00000432207	CD36	28.52	1.2172E-10
ENST00000426002	SHISA5	28.51	1.2172E-10

ENST00000262812	COPE	28.49	1.2172E-10
ENST00000333256	TSSC4	28.48	1.2172E-10
ENST00000510283	SPAG9	28.46	1.2172E-10
ENST00000299767	HSP90B1	28.45	1.2172E-10
ENST00000278671	LAMTOR1	28.45	1.2172E-10
ENST00000325285	FBXW5	28.44	1.2172E-10
ENST00000563468	GSPT1	28.44	1.2172E-10
ENST00000369306	PEX11B	28.41	1.2172E-10
ENST00000552909	LIMA1	28.39	1.2172E-10
ENST00000216259	PMM1	28.39	1.2172E-10
ENST00000472642	RHEB	28.39	1.2172E-10
ENST00000529826	BCLAF1	28.38	1.2172E-10
ENST00000369642	RHOC	28.35	1.2172E-10
ENST00000301935	UBXN1	28.33	1.2172E-10
ENST00000317534	MRPS24	28.32	1.2172E-10
ENST00000360079	XRCC6	28.32	1.2172E-10
ENST00000397615	RNH1	28.32	1.2172E-10
ENST00000619599	None	28.32	1.2172E-10
ENST00000281141	CDC123	28.31	1.2172E-10
ENST00000532097	PSMD13	28.30	1.2172E-10
ENST00000301727	E4F1	28.30	1.2172E-10
ENST00000374492	EDEM2	28.30	1.2172E-10
ENST00000248244	TICAM1	28.29	1.2172E-10
ENST00000354488	DDRGK1	28.28	1.2172E-10
ENST00000353801	HSP90AB1	28.28	1.2172E-10
ENST00000622181	None	28.27	1.2172E-10
ENST00000534624	HSPA8	28.27	1.2172E-10
ENST00000529691	ZNF202	28.26	1.2172E-10
ENST00000358514	PSMB10	28.26	1.2172E-10
ENST00000311008	SPNS1	28.24	1.2172E-10
ENST00000295920	GMPS	28.24	1.2172E-10
ENST00000502499	MATR3	28.22	1.2172E-10
ENST00000606999	None	28.22	1.2172E-10
ENST00000409920	ANXA4	28.22	1.2172E-10
ENST00000649817	KAT6A	28.21	1.2172E-10
ENST00000371674	UBE2V1	28.21	1.2172E-10
ENST00000476756	INSIG1	28.20	1.2172E-10
ENST00000372390	TSC22D3	28.20	1.2172E-10
ENST00000534182	ARCN1	28.19	1.2172E-10
ENST00000552981	SLC25A3	28.19	1.2172E-10
ENST00000359671	FN1	28.19	1.2509E-10
ENST00000527974	LGALS8	28.17	1.2172E-10
ENST00000244519	BTN3A3	28.15	1.2172E-10
ENST00000448451	DIAPH1	28.14	1.2172E-10
ENST00000418740	MRPS24	28.14	1.2172E-10

ENST00000268220	SEC11A	28.13	1.2172E-10
ENST00000216733	EFS	28.13	1.2172E-10
ENST00000303927	CAVIN3	28.11	1.2172E-10
ENST00000473244	MED15	28.11	1.2172E-10
ENST00000260956	SSB	28.11	1.2172E-10
ENST00000320172	TENT5A	28.10	1.2172E-10
ENST00000412318	ANKRD28	28.10	1.2172E-10
ENST00000304735	VASN	28.10	1.2172E-10
ENST00000447601	FAM3A	28.09	1.2172E-10
ENST00000029410	B4GALT7	28.08	1.2172E-10
ENST00000492773	EIF2B5	28.04	1.2693E-10
ENST00000479421	YWHAB	28.03	1.2756E-10
ENST00000267859	BNIP2	28.00	1.3043E-10
ENST00000394934	None	27.99	1.3043E-10
ENST00000580888	None	27.96	1.3043E-10
ENST00000406106	PPP1R7	27.94	1.3043E-10
ENST00000420247	SCYL1	27.93	1.3043E-10
ENST00000359479	SLC33A1	27.92	1.3043E-10
ENST00000194530	STRADB	27.91	1.3043E-10
ENST00000339093	NOSIP	27.91	1.3043E-10
ENST00000518047	DIAPH1	27.91	1.3043E-10
ENST00000525095	VPS26B	27.91	1.3043E-10
ENST00000357519	FIBP	27.90	1.3043E-10
ENST00000295704	RNF25	27.90	1.3043E-10
ENST00000435572	NFE2	27.89	1.3043E-10
ENST00000370854	ZDHHC16	27.89	1.3043E-10
ENST00000495186	EBP	27.88	1.3043E-10
ENST00000614294	DUSP14	27.88	1.3043E-10
ENST00000447532	CYB561A3	27.86	1.3043E-10
ENST00000368235	NAXE	27.86	1.3043E-10
ENST00000377710	MGME1	27.86	1.3043E-10
ENST00000548044	NAP1L1	27.86	1.3043E-10
ENST00000253794	VPS25	27.84	1.3043E-10
ENST00000420250	ASNSD1	27.84	1.3043E-10
ENST00000403490	IMP3	27.83	1.3043E-10
ENST00000535923	PHB2	27.83	1.3043E-10
ENST00000265038	ERCC8	27.83	1.3043E-10
ENST00000537057	PRKAB1	27.82	1.3043E-10
ENST00000263867	CAPG	27.82	1.3043E-10
ENST00000647446	GPI	27.81	1.3043E-10
ENST00000644593	CRYL1	27.79	1.3043E-10
ENST00000577789	FLOT2	27.78	1.3043E-10
ENST00000653961	TPM4	27.78	1.3043E-10
ENST00000367804	SLC19A2	27.78	1.3043E-10
ENST00000396969	DNAJB1	27.78	1.3043E-10

ENST00000301458	CD320	27.77	1.3043E-10
ENST00000292807	AP2M1	27.77	1.3043E-10
ENST00000235835	AKR7A2	27.76	1.3043E-10
ENST00000507528	PDCD6	27.76	1.3043E-10
ENST00000611266	OSBPL8	27.76	1.3043E-10
ENST00000538721	PCYT2	27.75	1.3043E-10
ENST00000372868	CCN5	27.75	1.3043E-10
ENST00000368954	CERS2	27.75	1.3043E-10
ENST00000397415	RPL7L1	27.75	1.3043E-10
ENST00000263309	CLNS1A	27.75	1.3043E-10
ENST00000526049	SELENOS	27.74	1.3492E-10
ENST00000440960	PRELID3A	27.74	1.3043E-10
ENST00000442046	MACF1	27.74	1.3043E-10
ENST00000357849	COPS5	27.73	1.3043E-10
ENST00000379412	LDHA	27.73	1.3043E-10
ENST00000673204	None	27.73	1.3043E-10
ENST00000252288	GAMT	27.73	1.3043E-10
ENST00000541050	CORO1C	27.70	1.3043E-10
ENST00000636882	RILPL1	27.70	1.3043E-10
ENST00000370053	STMN3	27.70	1.3043E-10
ENST00000294258	ZFPL1	27.70	1.3043E-10
ENST00000398030	APEX1	27.69	1.3043E-10
ENST00000353245	YWHAZ	27.69	1.3043E-10
ENST00000369332	MAP3K7	27.68	1.3043E-10
ENST00000450852	MOCS2	27.68	1.3043E-10
ENST00000347048	PTPA	27.68	1.3043E-10
ENST00000444739	CASP4	27.67	1.3043E-10
ENST00000203630	MLF2	27.67	1.3043E-10
ENST00000616143	MARCHF8	27.65	1.3043E-10
ENST00000324862	PRPF3	27.65	1.3043E-10
ENST00000430379	SLC25A20	27.64	1.3043E-10
ENST00000503617	FAM200B	27.62	1.3043E-10
ENST00000522098	CLU	27.62	1.3043E-10
ENST00000424922	CALD1	27.62	1.3043E-10
ENST00000378546	FAAP20	27.62	1.3043E-10
ENST00000641927	PHGDH	27.62	1.3043E-10
ENST00000659595	TPM4	27.61	1.3043E-10
ENST00000588991	GPI	27.61	1.3043E-10
ENST00000358139	PDE1A	27.61	1.3043E-10
ENST00000272424	TPRKB	27.60	1.3043E-10
ENST00000357052	RAB4B	27.60	1.3043E-10
ENST00000449349	THNSL2	27.59	1.3043E-10
ENST00000452857	ELMO2	27.59	1.3043E-10
ENST00000522223	RASA4	27.58	1.3043E-10
ENST00000428192	OTUB1	27.58	1.3043E-10

ENST00000418625	COPS6	27.57	1.3043E-10
ENST00000326335	CUL4A	27.57	1.3043E-10
ENST00000396265	TSPO	27.57	1.3043E-10
ENST00000435345	SCP2	27.56	1.3043E-10
ENST00000227157	LDHA	27.55	1.3043E-10
ENST00000672078	None	27.55	1.3043E-10
ENST00000318158	GRHPR	27.55	1.3043E-10
ENST00000476163	CAMTA1	27.55	1.3043E-10
ENST00000650247	IGFBP6	27.55	1.3043E-10
ENST00000217971	PGRMC1	27.54	1.3043E-10
ENST00000535589	ACTR3	27.54	1.3043E-10
ENST00000520367	ENDOV	27.53	1.3043E-10
ENST00000543485	MMP2	27.53	1.3043E-10
ENST00000381140	GTF3A	27.53	1.3043E-10
ENST00000342407	INSIG1	27.52	1.3043E-10
ENST00000536442	CCDC91	27.52	1.3043E-10
ENST00000374450	EIF6	27.52	1.5660E-10
ENST00000314673	SNX14	27.52	1.3043E-10
ENST00000479595	None	27.52	1.3043E-10
ENST00000555932	MAX	27.51	1.3049E-10
ENST00000315808	PHF20L1	27.51	1.3049E-10
ENST00000376488	OTUD5	27.50	1.3138E-10
ENST00000493518	CACUL1	27.49	1.3171E-10
ENST00000220849	EIF3S6	27.49	1.3247E-10
ENST00000608083	ACOT7	27.47	1.3434E-10
ENST00000397133	EMC6	27.46	1.3459E-10
ENST00000319273	TAC1	27.46	1.3459E-10
ENST00000418391	DEF8	27.45	1.3492E-10
ENST00000481941	DHX36	27.44	1.3492E-10
ENST00000602866	PSMC3	27.44	1.3492E-10
ENST00000606142	LAP3	27.44	1.3967E-10
ENST00000380498	CD68	27.44	1.3492E-10
ENST00000427217	ARPC1B	27.44	1.3492E-10
ENST00000592299	SPHK1	27.44	1.3495E-10
ENST00000372508	ZNF691	27.43	1.3492E-10
ENST00000449058	TOM1	27.43	1.3492E-10
ENST00000648694	PEG3	27.43	1.3492E-10
ENST00000266263	MTFP1	27.42	1.5231E-10
ENST00000395047	VDAC1	27.41	1.3657E-10
ENST00000376809	HLA-A	27.41	1.5601E-10
ENST00000322776	NDUFV1	27.41	1.5660E-10
ENST00000648869	None	27.41	1.3657E-10
ENST00000380985	NLN	27.41	1.3657E-10
ENST00000560765	None	27.39	1.3818E-10
ENST00000432261	RNF24	27.39	1.4097E-10

ENST00000221855	TBCB	27.38	1.4032E-10
ENST00000544301	HEL113	27.37	1.4097E-10
ENST00000380625	FAM9C	27.36	1.4097E-10
ENST00000610455	DEF8	27.36	1.4097E-10
ENST00000345523	None	27.35	1.4097E-10
ENST00000411774	None	27.35	1.4097E-10
ENST00000531289	RAB2A	27.34	1.4097E-10
ENST00000267973	WDR61	27.34	1.4097E-10
ENST00000624247	SMTN	27.34	1.4097E-10
ENST00000304511	TMEM126A	27.34	1.4097E-10
ENST00000326427	ITM2C	27.31	1.6727E-10
ENST00000373146	ST6GALNAC6	27.31	1.6727E-10
ENST00000396549	MFAP5	27.24	1.5660E-10
ENST00000547382	LMBR1L	27.24	1.5660E-10
ENST00000545312	PLXNC1	27.24	1.5660E-10
ENST00000407022	BSCL2	27.24	1.6998E-10
ENST00000572995	PCYT2	27.23	1.5660E-10
ENST00000556766	GNG2	27.22	1.5660E-10
ENST00000317058	SGF29	27.22	1.5660E-10
ENST00000333651	HMG20B	27.21	1.6248E-10
ENST00000497571	KLF6	27.21	1.5660E-10
ENST00000415688	STRADB	27.21	1.5660E-10
ENST00000391881	CYTH2	27.21	1.5660E-10
ENST00000527302	RPS2	27.20	1.5660E-10
ENST00000375446	NINJ1	27.20	1.5660E-10
ENST00000405867	HADHB	27.20	1.5660E-10
ENST00000395314	KTN1	27.20	1.5660E-10
ENST00000268668	NDUFB10	27.20	1.5660E-10
ENST00000397070	TM2D2	27.20	1.5660E-10
ENST00000509606	HSD17B4	27.17	1.6083E-10
ENST00000592290	FXYD5	27.17	1.6083E-10
ENST00000438169	KRR1	27.17	1.6083E-10
ENST00000474033	MXRA8	27.17	1.6083E-10
ENST00000415213	BNIP2	27.15	1.6325E-10
ENST00000613052	None	27.15	1.6325E-10
ENST00000277010	SIGMAR1	27.14	1.6727E-10
ENST00000521309	YWHAZ	27.14	1.6338E-10
ENST00000333449	TMEM179B	27.14	1.6727E-10
ENST00000463674	BACE2	27.13	1.6338E-10
ENST00000221801	FBL	27.13	1.6338E-10
ENST00000625241	None	27.13	1.6338E-10
ENST00000632796	None	27.13	1.6338E-10
ENST00000314392	DPM2	27.13	1.6338E-10
ENST00000305784	DTYMK	27.12	1.6338E-10
ENST00000423516	TKT	27.12	1.6338E-10

ENST00000581767	DDX42	27.12	1.6338E-10
ENST00000452462	RBMS3	27.12	1.6338E-10
ENST00000652974	CALM2	27.12	1.6727E-10
ENST00000569693	ADPGK	27.12	1.6338E-10
ENST00000416293	ALDH2	27.12	1.6338E-10
ENST00000366728	GUK1	27.12	1.6727E-10
ENST00000409197	DYNC1I2	27.11	1.6727E-10
ENST00000468043	SLC66A1L	27.11	1.6373E-10
ENST00000541435	FXYD5	27.11	1.6373E-10
ENST00000316626	GSK3B	27.10	1.6373E-10
ENST00000319725	FUBP3	27.10	1.6727E-10
ENST00000353332	MTMR14	27.10	1.6373E-10
ENST00000509359	TIMM8B	27.10	1.6373E-10
ENST00000359478	MFAP5	27.10	1.6727E-10
ENST00000395238	IP6K1	27.10	1.6727E-10
ENST00000377595	UCHL3	27.10	1.6727E-10
ENST00000426421	CAPZA2	27.10	1.6727E-10
ENST00000621955	None	27.10	1.6727E-10
ENST00000586425	GPI	27.09	1.6727E-10
ENST00000264080	GPR108	27.09	1.6727E-10
ENST00000407712	CBLB	27.08	1.6727E-10
ENST00000372257	ERI3	27.08	1.6727E-10
ENST00000324001	PRX	27.08	1.6727E-10
ENST00000351829	AKR1A1	27.08	1.6727E-10
ENST00000530996	CHMP4A	27.08	1.6727E-10
ENST00000643482	CHMP4A	27.08	1.6727E-10
ENST00000508076	TGFBI	27.08	1.6727E-10
ENST00000359208	DNTTIP2	27.08	1.6727E-10
ENST00000372838	CERCAM	27.05	1.6727E-10
ENST00000315758	MDH2	27.04	1.9542E-10
ENST00000304298	HSPB2	27.04	1.9542E-10
ENST00000367287	TIMM17A	27.03	1.6962E-10
ENST00000563060	ALDOA	27.02	1.6727E-10
ENST00000504102	SPOP	27.02	1.6727E-10
ENST00000393991	TSC22D4	27.02	1.6727E-10
ENST00000402799	MADD	27.01	1.6727E-10
ENST00000253099	MRPL4	27.01	1.6727E-10
ENST00000374706	CCNY	27.01	1.6727E-10
ENST00000343827	TCF12	27.01	1.7309E-10
ENST00000383694	FILIP1L	27.01	1.6727E-10
ENST00000514505	BTF3	27.00	1.7323E-10
ENST00000487747	SMC4	27.00	1.6727E-10
ENST00000310118	PSMD2	27.00	1.6727E-10
ENST00000620761	ACD	26.99	1.7274E-10
ENST00000439741	MTMR11	26.99	1.6727E-10

ENST00000380546	SERPINB6	26.98	1.6727E-10
ENST00000444840	MRPS25	26.98	1.6727E-10
ENST00000335698	ATP5F1C	26.98	1.6727E-10
ENST00000651476	UROD	26.98	1.6727E-10
ENST00000282058	HAUS1	26.98	1.6727E-10
ENST00000566113	CDIPT	26.98	1.6727E-10
ENST00000558653	DTWD1	26.98	1.6727E-10
ENST00000527350	FEZ1	26.98	1.6727E-10
ENST00000369329	MAP3K7	26.96	1.6943E-10
ENST00000412584	CADPS2	26.96	1.6943E-10
ENST00000215375	ATP5F1D	26.96	1.6961E-10
ENST00000527899	CRYAB	26.95	1.6962E-10
ENST00000419121	BSDC1	26.95	1.6962E-10
ENST00000345264	RSU1	26.95	1.6962E-10
ENST00000458167	GPN1	26.94	1.6998E-10
ENST00000476671	KMT2E	26.94	1.9967E-10
ENST00000530731	TBRG1	26.93	1.9967E-10
ENST00000437787	CD99L2	26.93	1.7309E-10
ENST00000371514	SCP2	26.93	1.9967E-10
ENST00000447566	SLC25A17	26.93	1.9967E-10
ENST00000397408	TSPAN4	26.92	1.7323E-10
ENST00000299438	CYB5A	26.92	1.9967E-10
ENST00000589737	POLR2E	26.91	1.7323E-10
ENST00000369637	RHOC	26.91	1.7323E-10
ENST00000547568	DCN	26.91	1.7323E-10
ENST00000462138	TKT	26.91	1.7323E-10
ENST00000393028	GIPC1	26.91	1.7354E-10
ENST00000460039	TMCO3	26.90	1.7466E-10
ENST00000421097	COASY	26.88	1.7811E-10
ENST00000430553	LDHA	26.87	1.7811E-10
ENST00000620312	None	26.87	1.7811E-10
ENST00000672996	None	26.87	1.7811E-10
ENST00000373166	TRAPPC3	26.87	1.7811E-10
ENST00000380946	KLF6	26.87	1.7811E-10
ENST00000309244	ETFB	26.87	1.7811E-10
ENST00000404295	NMRAL1	26.86	1.7811E-10
ENST00000616587	None	26.86	1.7811E-10
ENST00000249736	SLTM	26.86	1.7857E-10
ENST00000558265	EIF5	26.86	1.7860E-10
ENST00000614273	CYP1B1	26.86	1.7882E-10
ENST00000464228	ITGB1BP1	26.85	1.7893E-10
ENST00000465759	CCDC174	26.85	1.7882E-10
ENST00000596655	C19orf48	26.85	1.7882E-10
ENST00000515017	ANXA5	26.85	1.7882E-10
ENST00000265963	GTF2H1	26.84	1.8240E-10

ENST00000672527	GTF2H1	26.84	1.8240E-10
ENST00000430330	SCP2	26.83	1.8240E-10
ENST00000462330	TXNRD2	26.83	1.8240E-10
ENST00000279270	None	26.82	1.9967E-10
ENST00000319471	SORBS2	26.82	1.8406E-10
ENST00000524958	C11orf98	26.81	1.8453E-10
ENST00000644285	ANKRD11	26.81	1.8533E-10
ENST00000403712	SH3YL1	26.81	1.8453E-10
ENST00000303731	TRAPPC1	26.81	1.8453E-10
ENST00000377139	IRF3	26.81	1.8453E-10
ENST00000553300	HNRNPC	26.80	1.8453E-10
ENST00000362052	ENSA	26.80	1.8453E-10
ENST00000369314	POLR3GL	26.76	1.9259E-10
ENST00000518374	COPS5	26.76	1.9341E-10
ENST00000586636	CIRBP	26.76	1.9341E-10
ENST00000371663	RABL6	26.75	1.9404E-10
ENST00000381570	RNF6	26.75	1.9404E-10
ENST00000616346	None	26.75	1.9404E-10
ENST00000617832	TIMM22	26.74	1.9404E-10
ENST00000613269	TIMM22	26.74	1.9404E-10
ENST00000403747	UBE2I	26.74	1.9967E-10
ENST00000587994	ACAA2	26.74	1.9967E-10
ENST00000598418	JOSD2	26.74	1.9967E-10
ENST00000554507	KTN1	26.73	1.9967E-10
ENST00000608443	FKBP8	26.73	1.9967E-10
ENST00000372554	SH3GLB2	26.72	1.9572E-10
ENST00000399523	UFD1	26.72	1.9572E-10
ENST00000464988	RBM5	26.72	1.9572E-10
ENST00000472931	ETV1	26.72	1.9967E-10
ENST00000584895	RPL17-C18orf32	26.72	1.9572E-10
ENST00000539040	RIT1	26.72	1.9572E-10
ENST00000251074	NUP37	26.72	1.9572E-10
ENST00000498639	CPB1	26.72	1.9572E-10
ENST00000510338	ZBTB38	26.72	1.9572E-10
ENST00000615871	AUTS2	26.71	1.9643E-10
ENST00000574924	ANAPC11	26.71	1.9967E-10
ENST00000636409	ATP6AP2	26.71	1.9674E-10
ENST00000622898	DTNBP1	26.71	1.9967E-10
ENST00000535411	MFAP5	26.70	1.9867E-10
ENST00000648109	C1orf21	26.70	1.9867E-10
ENST00000417750	LMBR1L	26.70	1.9876E-10
ENST00000473629	TBRG1	26.69	2.0027E-10
ENST00000397424	EPC2	26.69	1.9967E-10
ENST00000648046	GRB2	26.68	1.9967E-10
ENST00000586802	HDAC5	26.68	2.0131E-10

ENST00000323213	EIF3M	26.68	1.9967E-10
ENST00000374643	YIPF6	26.68	1.9967E-10
ENST00000642241	CBLB	26.67	1.9967E-10
ENST00000372259	ERI3	26.67	1.9967E-10
ENST00000487106	LAMTOR2	26.67	1.9967E-10
ENST00000392568	NUDT14	26.67	2.0177E-10
ENST00000469858	SMC4	26.67	1.9967E-10
ENST00000433983	ETFA	26.67	2.0125E-10
ENST00000344114	HERPUD1	26.67	2.0125E-10
ENST00000497931	ARMCX6	26.67	1.9967E-10
ENST00000467199	HEL97	26.66	2.0177E-10
ENST00000420846	CD63	26.66	2.0177E-10
ENST00000484262	WIPI2	26.66	2.0177E-10
ENST00000455824	ST13	26.66	1.9967E-10
ENST00000544392	CACUL1	26.65	1.9967E-10
ENST00000261623	CYBA	26.65	1.9967E-10
ENST00000305978	SCAND1	26.65	1.9967E-10
ENST00000531496	SYTL2	26.65	1.9967E-10
ENST00000398020	PIP4P1	26.65	2.0273E-10
ENST00000374436	EIF6	26.65	1.9967E-10
ENST00000357355	ADGRE5	26.65	2.0273E-10
ENST00000584289	PSMD12	26.65	1.9967E-10
ENST00000568886	VAC14	26.65	2.0273E-10
ENST00000345519	CLTA	26.64	1.9967E-10
ENST00000532444	EIF3M	26.64	1.9967E-10
ENST00000055077	RFC2	26.64	1.9967E-10
ENST00000493831	ETV1	26.64	1.9967E-10
ENST00000545822	HADHB	26.64	1.9967E-10
ENST00000265304	SSBP1	26.64	2.0333E-10
ENST00000570767	RSL1D1	26.63	2.0333E-10
ENST00000553966	DAAM1	26.63	1.9967E-10
ENST00000531405	ZNRD2	26.63	2.0333E-10
ENST00000443660	MRPL40	26.63	1.9967E-10
ENST00000642789	TPM4	26.63	1.9967E-10
ENST00000248451	PNKD	26.63	1.9967E-10
ENST00000303698	NFU1	26.62	1.9967E-10
ENST00000450863	GOLGA4	26.62	2.0333E-10
ENST00000352327	THYN1	26.62	2.0333E-10
ENST00000513231	UBE2K	26.62	2.0333E-10
ENST00000394048	ESYT1	26.62	2.0333E-10
ENST00000508500	None	26.62	1.9967E-10
ENST00000466494	IDH3B	26.62	1.9967E-10
ENST00000326134	MROH1	26.61	1.9967E-10
ENST00000515708	TSPAN17	26.61	1.9967E-10
ENST00000228922	OGFOD2	26.61	1.9967E-10

ENST00000438848	PPP1R2	26.60	1.9967E-10
ENST00000598626	GPR108	26.60	1.9967E-10
ENST00000272898	None	26.60	1.9967E-10
ENST00000445439	PPP2R5C	26.60	1.9967E-10
ENST00000471189	UQCRC1	26.60	1.9967E-10
ENST00000535020	NAP1L1	26.59	1.9967E-10
ENST00000526180	CRYAB	26.59	1.9967E-10
ENST00000191018	CTSA	26.59	1.9967E-10
ENST00000457167	DNAJC7	26.59	1.9967E-10
ENST00000372433	HYI	26.58	2.0333E-10
ENST00000412476	PHYHD1	26.58	2.0333E-10
ENST00000276704	C8orf76	26.58	1.9967E-10
ENST00000520210	ELOC	26.58	1.9967E-10
ENST00000555431	ATP6V1D	26.58	1.9967E-10
ENST00000400052	CLIC1	26.58	1.9967E-10
ENST00000375784	CLIC1	26.58	1.9967E-10
ENST00000425464	CLIC1	26.58	1.9967E-10
ENST00000422167	CLIC1	26.58	1.9967E-10
ENST00000418285	CLIC1	26.58	1.9967E-10
ENST00000456863	CLIC1	26.58	1.9967E-10
ENST00000457485	CLIC1	26.58	1.9967E-10
ENST00000637071	POLR1D	26.58	1.9967E-10
ENST00000483765	RPL21	26.58	1.9970E-10
ENST00000450235	MAD1L1	26.57	2.0333E-10
ENST00000426960	ARL6IP4	26.57	2.0019E-10
ENST00000422957	KRT223P	26.57	2.0021E-10
ENST00000348411	FTSJ1	26.57	2.0023E-10
ENST00000539887	STRAP	26.56	2.0027E-10
ENST00000490235	VEPH1	26.56	2.0120E-10
ENST00000478247	DVL3	26.56	2.0389E-10
ENST00000427895	GNB2	26.55	2.0125E-10
ENST00000425172	INSIG1	26.55	2.0131E-10
ENST00000395560	UPP1	26.55	2.0464E-10
ENST00000548921	SCRN3	26.55	2.0131E-10
ENST00000309328	ZNRD2	26.55	2.3169E-10
ENST00000441728	UBE2F	26.55	2.0133E-10
ENST00000262722	FBLN1	26.55	2.0136E-10
ENST00000403633	BUD31	26.54	2.0177E-10
ENST00000378891	DVL1	26.54	2.0946E-10
ENST00000419582	PTPA	26.54	2.0177E-10
ENST00000591099	PLEKHJ1	26.54	2.0177E-10
ENST00000350605	PYCARD	26.54	2.0177E-10
ENST00000380996	TSSC4	26.53	2.0177E-10
ENST00000468479	FDPS	26.53	2.1139E-10
ENST00000246784	BCL2L12	26.53	2.0177E-10

ENST00000267869	GTF2A2	26.53	2.0177E-10
ENST00000551446	NFYB	26.52	2.0255E-10
ENST00000254940	NIP7	26.52	2.0273E-10
ENST00000558582	NUSAP1	26.52	2.0273E-10
ENST00000648280	COPA	26.52	2.0273E-10
ENST00000526975	CFL1	26.51	2.1218E-10
ENST00000221418	ECH1	26.51	2.0273E-10
ENST00000634245	ECH1	26.51	2.0273E-10
ENST00000560979	FGF7	26.51	2.1361E-10
ENST00000487888	DENND10	26.51	2.0275E-10
ENST00000567958	PSMD7	26.51	2.0275E-10
ENST00000425490	TRIM22	26.50	2.1424E-10
ENST00000409953	UBE2F	26.50	2.0333E-10
ENST00000579755	CDKN2A	26.49	2.0333E-10
ENST00000517671	NPM1	26.49	2.2796E-10
ENST00000475043	PDPN	26.49	2.0333E-10
ENST00000356708	ATP5F1C	26.49	2.0333E-10
ENST00000395678	TYMP	26.48	2.1911E-10
ENST00000236980	KIAA0971	26.48	2.0333E-10
ENST00000467324	TSN	26.48	2.0333E-10
ENST00000619865	None	26.47	2.0389E-10
ENST00000534940	ASMTL	26.47	2.1965E-10
ENST00000545215	LDHA	26.47	2.1965E-10
ENST00000672754	None	26.47	2.1965E-10
ENST00000589934	APBA3	26.47	2.0395E-10
ENST00000601048	SELENOW	26.47	2.0395E-10
ENST00000467315	PFKL	26.47	2.0576E-10
ENST00000521514	ATP6V1C1	26.46	2.0576E-10
ENST00000578663	CDK5RAP3	26.46	2.0584E-10
ENST00000628684	SLTM	26.46	2.0584E-10
ENST00000392757	LHPP	26.46	2.3526E-10
ENST00000272227	PDIA6	26.46	2.3526E-10
ENST00000417292	SSB	26.45	2.0687E-10
ENST00000644774	ACTG1	26.45	2.0687E-10
ENST00000530796	GPAA1	26.45	2.0698E-10
ENST00000467084	MRPS24	26.45	2.0708E-10
ENST00000403094	FASTKD2	26.45	2.2152E-10
ENST00000647559	None	26.44	2.0772E-10
ENST00000404405	PPP1R7	26.44	2.0901E-10
ENST00000626873	SH3YL1	26.42	2.1198E-10
ENST00000188376	SLC25A3	26.42	2.1218E-10
ENST00000449653	COMT	26.42	2.2472E-10
ENST00000377911	RSU1	26.42	2.1245E-10
ENST00000478935	ARL6IP5	26.41	2.2485E-10
ENST00000509182	DIMT1	26.41	2.1371E-10

ENST00000531551	CTSB	26.41	2.1371E-10
ENST00000571105	PCYT2	26.41	2.1381E-10
ENST00000476170	PHYKPL	26.40	2.2485E-10
ENST00000494864	CYP1B1	26.40	2.2485E-10
ENST00000503411	SDAD1	26.40	2.2485E-10
ENST00000549115	CHURC1	26.40	2.2485E-10
ENST00000529581	SYTL2	26.40	2.2485E-10
ENST00000354914	SCARA5	26.39	2.1715E-10
ENST00000528265	SPCS2	26.39	2.1891E-10
ENST00000589265	GRN	26.39	2.1891E-10
ENST00000244217	MCEE	26.38	2.2001E-10
ENST00000498491	FLNA	26.37	2.2012E-10
ENST00000515430	FAM200B	26.37	2.2061E-10
ENST00000578105	SKA2	26.36	2.2152E-10
ENST00000377254	THNSL2	26.36	2.2152E-10
ENST00000548176	IGFBP6	26.36	2.2152E-10
ENST00000506182	SNX14	26.36	2.2152E-10
ENST00000368690	CHTOP	26.36	2.2388E-10
ENST00000345358	BAX	26.36	2.2388E-10
ENST00000426907	HUWE1	26.35	2.2152E-10
ENST00000626748	None	26.35	2.2152E-10
ENST00000302392	TMEM42	26.35	2.2152E-10
ENST00000420940	WDR48	26.35	2.2172E-10
ENST00000550787	TMEM19	26.35	2.2472E-10
ENST00000329463	DHX36	26.34	2.2388E-10
ENST00000249442	MTX2	26.34	2.2409E-10
ENST00000533892	SYTL2	26.33	2.2472E-10
ENST00000342572	CYBC1	26.32	2.2472E-10
ENST00000392359	CENPX	26.32	2.2472E-10
ENST00000306704	CENPX	26.32	2.2472E-10
ENST00000548400	MYL6	26.32	2.2472E-10
ENST00000573763	SERPINF1	26.32	2.2472E-10
ENST00000586988	DNMT1	26.32	2.2472E-10
ENST00000547303	DDIT3	26.32	2.2472E-10
ENST00000500655	TIFA	26.32	2.2472E-10
ENST00000526361	SPCS2	26.32	2.2485E-10
ENST00000620248	BCL10	26.32	2.2485E-10
ENST00000392324	RFXANK	26.32	2.2485E-10
ENST00000391855	GGPS1	26.31	3.0200E-10
ENST00000502495	SCLT1	26.31	2.2485E-10
ENST00000507773	SLC35B1	26.31	2.2485E-10
ENST00000564404	TRAPPC2L	26.31	2.2485E-10
ENST00000416967	TXN2	26.31	2.2485E-10
ENST00000396634	HLA-A	26.30	2.2485E-10
ENST00000458039	RALBP1	26.30	2.2485E-10

ENST00000465650	KCTD3	26.30	2.2485E-10
ENST00000450599	DDX5	26.30	2.2530E-10
ENST00000331821	RANBP1	26.30	2.2530E-10
ENST00000414154	SMIM19	26.29	3.0845E-10
ENST00000479690	WIPI2	26.29	2.2534E-10
ENST00000540056	MGST1	26.29	2.2534E-10
ENST00000394701	AIMP1	26.29	2.2534E-10
ENST00000397614	RNH1	26.29	2.2534E-10
ENST00000618184	None	26.29	2.2534E-10
ENST00000257904	CDK4	26.28	2.2683E-10
ENST00000609777	LSP1P4	26.28	2.2683E-10
ENST00000418862	SNX14	26.28	2.2683E-10
ENST00000610832	KLF4	26.28	2.2683E-10
ENST00000497054	MOB1A	26.27	2.2719E-10
ENST00000547252	SLC38A2	26.27	2.2719E-10
ENST00000374741	OXLD1	26.27	2.2719E-10
ENST00000549384	CORO1C	26.27	2.2719E-10
ENST00000507503	LUC7L3	26.27	2.2796E-10
ENST00000473598	LMNA	26.26	2.2796E-10
ENST00000484996	GLUL	26.26	2.2796E-10
ENST00000519627	EIF3E	26.26	2.2796E-10
ENST00000596260	PTGIR	26.26	2.2796E-10
ENST00000496844	THNSL2	26.26	2.4674E-10
ENST00000539134	RELT	26.26	2.4674E-10
ENST00000425728	JKAMP	26.26	3.1201E-10
ENST00000312419	POLD4	26.26	2.2796E-10
ENST00000476436	ACOT13	26.26	2.2796E-10
ENST00000554943	PPP4R3A	26.26	2.2796E-10
ENST00000356187	RNH1	26.26	2.2796E-10
ENST00000454828	TRIM22	26.26	2.2796E-10
ENST00000617351	None	26.26	2.2796E-10
ENST00000506411	GLRB	26.24	2.3166E-10
ENST00000403658	SH3YL1	26.24	2.3166E-10
ENST00000484711	TTLL12	26.24	2.3166E-10
ENST00000590956	PRELID3A	26.24	2.3166E-10
ENST00000503368	UBE2K	26.24	2.3169E-10
ENST00000560338	EIF5	26.24	2.3169E-10
ENST00000238651	ACOT2	26.23	2.3357E-10
ENST00000476658	DFFA	26.23	2.3357E-10
ENST00000512913	SUB1	26.23	2.3357E-10
ENST00000493900	NAA50	26.22	2.3357E-10
ENST00000544848	H2AFJ	26.22	2.3526E-10
ENST00000558985	ANXA2	26.20	2.4703E-10
ENST00000253457	EMC8	26.19	2.4264E-10
ENST00000422453	CNBP	26.18	2.4403E-10

ENST00000575087	ACTG1	26.18	2.4403E-10
ENST00000454694	OGFOD2	26.17	2.4703E-10
ENST00000552635	TMBIM6	26.17	2.4703E-10
ENST00000529579	RAB2A	26.17	2.4703E-10
ENST00000467810	TPR	26.17	2.4703E-10
ENST00000411850	TOM1	26.16	2.5007E-10
ENST00000488542	DMKN	26.14	2.5480E-10
ENST00000311487	HMGA1	26.14	2.5480E-10
ENST00000570054	None	26.14	2.5732E-10
ENST00000591922	RNMT	26.13	2.5732E-10
ENST00000538057	PTMS	26.13	2.5773E-10
ENST00000549561	MRPL42	26.12	2.5896E-10
ENST00000540651	ARF1	26.11	2.6139E-10
ENST00000620430	WASHC4	26.11	2.6139E-10
ENST00000491523	RPL13	26.11	2.6345E-10
ENST00000357857	CLN3	26.10	2.6388E-10
ENST00000468318	CNIH4	26.10	2.6434E-10
ENST00000340006	CSRP1	26.10	2.6474E-10
ENST00000355151	MRPL52	26.10	2.6474E-10
ENST00000467911	NCK1	26.10	2.6474E-10
ENST00000591636	ERCC1	26.09	2.6707E-10
ENST00000448741	HEBP2	26.09	2.6707E-10
ENST00000398712	SHARPIN	26.09	2.6707E-10
ENST00000290949	ATP6V0D1	26.08	2.6834E-10
ENST00000586375	RAD23A	26.08	2.6894E-10
ENST00000420267	MRPS25	26.08	2.6894E-10
ENST00000550524	TMEM19	26.07	2.7014E-10
ENST00000571732	YWHAE	26.07	2.7107E-10
ENST00000616643	None	26.07	2.7107E-10
ENST00000456170	ERI3	26.07	2.7110E-10
ENST00000335174	ANKRD37	26.07	2.7110E-10
ENST00000555568	NSFL1C	26.06	2.7146E-10
ENST00000512419	UCHL1	26.06	2.7376E-10
ENST00000509889	COPG1	26.06	2.7376E-10
ENST00000637327	ATP6AP2	26.06	2.7376E-10
ENST00000436787	WDR33	26.05	2.7414E-10
ENST00000482504	SH3GLB1	26.05	2.7443E-10
ENST00000216194	ADSL	26.05	2.7443E-10
ENST00000596149	CLPP	26.05	2.7443E-10
ENST00000541233	CD27	26.04	2.7741E-10
ENST00000481386	CD81	26.04	2.7741E-10
ENST00000461997	RHNO1	26.04	2.7741E-10
ENST00000543709	TAOK3	26.03	2.7741E-10
ENST00000555324	DDX24	26.03	2.7741E-10
ENST00000627947	None	26.03	2.7741E-10

ENST00000587727	DNAJC7	26.03	2.7790E-10
ENST00000549338	SLC25A3	26.03	3.1061E-10
ENST00000353555	BSG	26.03	2.7893E-10
ENST00000255688	PLAAT4	26.03	2.7893E-10
ENST00000243112	SMUG1	26.02	2.7927E-10
ENST00000536588	None	26.02	2.7927E-10
ENST00000443913	CTSK	26.01	2.8421E-10
ENST00000620860	EIF2B3	26.01	2.8421E-10
ENST00000250124	MPDU1	26.01	3.8684E-10
ENST00000344085	ZNF880	26.00	2.8483E-10
ENST00000446293	BSDC1	26.00	2.8506E-10
ENST00000356936	NCL	26.00	2.8483E-10
ENST00000291839	ST6GALNAC6	26.00	2.8483E-10
ENST00000565833	AMZ2P1	26.00	2.8483E-10
ENST00000306049	TEFM	26.00	2.8483E-10
ENST00000452080	TBRG1	26.00	2.8483E-10
ENST00000486355	DNAJC19	26.00	2.8483E-10
ENST00000427250	LSR	25.99	2.8609E-10
ENST00000311534	ISCA1	25.99	2.8752E-10
ENST00000402468	C1GALT1	25.99	2.8752E-10
ENST00000495720	RAB13	25.99	2.8752E-10
ENST00000642236	TOPBP1	25.99	2.8752E-10
ENST00000378576	MEST	25.98	2.8888E-10
ENST00000577427	SCO1	25.97	2.9306E-10
ENST00000589631	GIPC1	25.97	2.9306E-10
ENST00000598955	GPR108	25.97	2.9306E-10
ENST00000418289	FASTKD2	25.96	2.9410E-10
ENST00000219542	METRN	25.96	2.9410E-10
ENST00000536657	WASF2	25.95	2.9671E-10
ENST00000442805	RALY	25.95	2.9884E-10
ENST00000557670	TMED10	25.95	2.9888E-10
ENST00000560585	GCNT3	25.94	3.0062E-10
ENST00000494131	CYB5A	25.94	3.0216E-10
ENST00000260359	NUSAP1	25.93	3.0275E-10
ENST00000294489	PDPN	25.93	3.0565E-10
ENST00000557658	VIPAS39	25.92	3.0643E-10
ENST00000597551	FLT3LG	25.92	3.0779E-10
ENST00000493356	PFDN4	25.91	3.0845E-10
ENST00000596435	FLT3LG	25.91	3.0845E-10
ENST00000596975	FCGRT	25.91	3.0845E-10
ENST00000422285	PDHA1	25.91	3.1647E-10
ENST00000428260	ZNF646	25.91	3.1647E-10
ENST00000246337	UROD	25.91	3.0845E-10
ENST00000567648	VAC14	25.91	3.0845E-10
ENST00000502897	SUB1	25.91	3.0845E-10

ENST00000651860	LAMA4	25.91	3.0845E-10
ENST00000254878	RIDA	25.91	3.0845E-10
ENST00000634702	MAP4K4	25.90	3.0845E-10
ENST00000482094	MUTYH	25.90	3.0845E-10
ENST00000616120	NBPF14	25.90	3.0845E-10
ENST00000394817	SIL1	25.90	3.0845E-10
ENST00000457974	GYS1	25.90	3.0860E-10
ENST00000582475	SNRPD1	25.89	3.1061E-10
ENST00000588776	CAPS	25.89	3.1061E-10
ENST00000398033	TMBIM4	25.89	3.1061E-10
ENST00000531339	SIPA1	25.89	3.1061E-10
ENST00000366654	FAM89A	25.89	3.1061E-10
ENST00000378990	CAP2	25.89	3.1061E-10
ENST00000599537	NOSIP	25.88	3.1403E-10
ENST00000535139	SPX	25.87	3.1770E-10
ENST00000573251	RSL1D1	25.87	3.1770E-10
ENST00000640289	None	25.87	3.1819E-10
ENST00000530628	CDKN2A	25.87	3.1770E-10
ENST00000554736	GNG2	25.87	3.1770E-10
ENST00000616502	EPHB4	25.85	3.2238E-10
ENST00000263354	NAPA	25.84	3.5466E-10
ENST00000534028	SLC25A45	25.84	3.2668E-10
ENST00000651658	RAD50	25.84	3.2668E-10
ENST00000369905	ACTR1A	25.83	3.2994E-10
ENST00000485487	REV1	25.82	3.3198E-10
ENST00000409453	DYNC1I2	25.82	3.3198E-10
ENST00000485435	SURF4	25.82	3.3198E-10
ENST00000626303	None	25.82	3.3198E-10
ENST00000483839	GTPBP4	25.82	3.3856E-10
ENST00000500777	SEC31A	25.82	3.3358E-10
ENST00000555597	RNASE4	25.82	3.3358E-10
ENST00000413379	SAE1	25.81	3.3592E-10
ENST00000532339	CD44	25.80	3.3897E-10
ENST00000477783	TCEA2	25.80	3.4015E-10
ENST00000419828	GNB2	25.80	3.5239E-10
ENST00000530428	MTCH2	25.78	3.4674E-10
ENST00000584350	AMZ2	25.78	3.4674E-10
ENST00000642838	None	25.78	3.4674E-10
ENST00000465584	NKTR	25.78	3.4717E-10
ENST00000315491	TUBB3	25.78	3.4674E-10
ENST00000439780	UBE2F	25.78	3.4674E-10
ENST00000538107	MFAP5	25.78	3.4674E-10
ENST00000560682	SLTM	25.78	3.4719E-10
ENST00000562125	TRAPPC2L	25.77	3.4719E-10
ENST00000397514	UBE2I	25.77	3.4719E-10

ENST00000518918	MRPL13	25.77	3.4719E-10
ENST00000407461	POLR3H	25.77	3.4719E-10
ENST00000591583	COASY	25.77	3.4719E-10
ENST00000446041	SLC25A36	25.77	3.4719E-10
ENST00000586760	CALR	25.76	3.4977E-10
ENST00000594092	VRK3	25.76	5.2455E-10
ENST00000549992	NUDT4	25.76	3.5182E-10
ENST00000572250	RABEP1	25.76	3.5204E-10
ENST00000566835	MLST8	25.75	3.5283E-10
ENST00000495267	KMT2E	25.75	3.5406E-10
ENST00000588118	DNMT1	25.75	4.3538E-10
ENST00000509022	FAM200B	25.74	3.5639E-10
ENST00000308153	H2AZ2	25.73	3.6196E-10
ENST00000614237	SENP3-EIF4A1	25.73	3.6196E-10
ENST00000233627	NDUFS7	25.73	3.6237E-10
ENST00000559412	MYO1E	25.73	3.6237E-10
ENST00000454220	PPP2R1A	25.72	3.6526E-10
ENST00000460327	DMKN	25.72	3.6526E-10
ENST00000503608	SNX14	25.72	3.6526E-10
ENST00000216330	FKBP3	25.71	3.6537E-10
ENST00000538396	MCRIP1	25.71	3.6537E-10
ENST00000673369	None	25.71	3.6537E-10
ENST00000426563	DYNLT2B	25.71	3.6599E-10
ENST00000492044	AZI2	25.71	3.6599E-10
ENST00000368301	LMNA	25.71	3.6667E-10
ENST00000647567	MED13L	25.71	3.6667E-10
ENST00000644527	SMARCE1	25.70	3.6793E-10
ENST00000602561	RPL7L1	25.70	3.6923E-10
ENST00000526267	EIF3M	25.70	3.7998E-10
ENST00000496858	PRKAB2	25.69	3.7502E-10
ENST00000322008	CD151	25.69	3.7502E-10
ENST00000347519	CHMP4A	25.69	3.7502E-10
ENST00000645308	None	25.69	3.7502E-10
ENST00000536981	SLC3A2	25.68	3.7760E-10
ENST00000458574	MPC2	25.68	3.7760E-10
ENST00000508218	LUC7L3	25.68	3.7760E-10
ENST00000554437	SCFD1	25.68	3.7760E-10
ENST00000368322	RIT1	25.67	3.7760E-10
ENST00000421687	PABPC4	25.67	3.7760E-10
ENST00000510934	UBE2K	25.67	3.7760E-10
ENST00000527794	SYTL2	25.67	3.7760E-10
ENST00000356950	H2BC12	25.67	3.7860E-10
ENST00000398409	CST3	25.67	3.7860E-10
ENST00000426541	RBSN	25.66	3.7998E-10
ENST00000466371	FTSJ1	25.66	3.7998E-10

ENST00000518793	PIEZO1	25.66	3.7998E-10
ENST00000375779	CLIC1	25.65	3.7998E-10
ENST00000383404	None	25.65	3.7998E-10
ENST00000423055	None	25.65	3.7998E-10
ENST00000423143	None	25.65	3.7998E-10
ENST00000423804	None	25.65	3.7998E-10
ENST00000434202	None	25.65	3.7998E-10
ENST00000438708	None	25.65	3.7998E-10
ENST00000498219	HPS1	25.65	3.7998E-10
ENST00000263688	SUCO	25.65	3.7998E-10
ENST00000392659	MRPL47	25.65	3.7998E-10
ENST00000495054	MTMR11	25.65	3.7998E-10
ENST00000559602	ETFA	25.65	3.7998E-10
ENST00000260502	BCAR3	25.65	3.8181E-10
ENST00000410071	EVA1A	25.65	3.8181E-10
ENST00000533729	ARL2	25.65	3.8181E-10
ENST00000538636	RHNO1	25.62	3.9283E-10
ENST00000539107	CCDC91	25.62	3.9283E-10
ENST00000585693	DNAJC7	25.62	3.9283E-10
ENST00000590238	APBA3	25.62	4.1867E-10
ENST00000448364	RALY	25.62	4.1867E-10
ENST00000540688	NDUFA9	25.62	3.9283E-10
ENST00000477985	BOD1	25.62	3.9405E-10
ENST00000395418	IFT20	25.61	4.0992E-10
ENST00000543979	None	25.60	4.0156E-10
ENST00000372484	CTSA	25.60	4.0156E-10
ENST00000396682	RPA3	25.60	4.0156E-10
ENST00000631280	THOC1	25.60	4.0156E-10
ENST00000592273	TRIR	25.60	4.0156E-10
ENST00000621097	RFC2	25.60	4.0385E-10
ENST00000368949	CERS2	25.59	4.0538E-10
ENST00000532577	ZNF143	25.59	4.0538E-10
ENST00000461234	ADAM15	25.58	4.0863E-10
ENST00000518696	MRPL13	25.58	4.0863E-10
ENST00000330188	TPM3	25.58	4.3423E-10
ENST00000618438	FHL1	25.58	4.0923E-10
ENST00000652033	TPP2	25.57	4.1178E-10
ENST00000397665	METTL26	25.57	4.1299E-10
ENST00000514121	SPOP	25.57	4.1299E-10
ENST00000361871	MSRB1	25.55	4.1935E-10
ENST00000451119	ZNF219	25.55	4.1935E-10
ENST00000395969	VPS41	25.54	4.2308E-10
ENST00000514685	SMUG1	25.54	4.2308E-10
ENST00000407025	PPP1R7	25.54	4.2308E-10
ENST00000460807	NKTR	25.54	5.3210E-10

ENST00000637763	SUPT3H	25.54	4.2759E-10
ENST00000553360	KTN1	25.53	4.2988E-10
ENST00000333552	RALY	25.53	4.2988E-10
ENST00000472253	PPP2CA	25.53	4.3383E-10
ENST00000625194	ADSL	25.53	4.3383E-10
ENST00000495958	CAAP1	25.53	4.9979E-10
ENST00000581858	FLII	25.51	4.3611E-10
ENST00000640412	None	25.51	4.3611E-10
ENST00000350771	H2AZ2	25.51	4.3611E-10
ENST00000517403	PABPC1	25.51	4.3611E-10
ENST00000615219	None	25.51	4.3611E-10
ENST00000352231	LGALS8	25.51	4.3729E-10
ENST00000461665	MDH2	25.51	4.3729E-10
ENST00000633112	None	25.51	4.3729E-10
ENST00000393193	NME1-NME2	25.51	4.3729E-10
ENST00000392508	DYNLL1	25.50	4.3794E-10
ENST00000393300	OLFML3	25.50	4.3794E-10
ENST00000525807	CTSH	25.50	4.3794E-10
ENST00000269298	SAT2	25.50	4.3966E-10
ENST00000521569	SPARC	25.49	4.4361E-10
ENST00000537368	GTF2H3	25.49	4.4361E-10
ENST00000587950	MICOS13	25.49	4.4361E-10
ENST00000382464	HEATR5A	25.49	4.4473E-10
ENST00000422978	None	25.48	4.4473E-10
ENST00000423382	None	25.48	4.4473E-10
ENST00000229725	NEU1	25.48	4.4473E-10
ENST00000437432	NEU1	25.48	4.4473E-10
ENST00000434496	NEU1	25.48	4.4473E-10
ENST00000373408	CCDC167	25.48	4.4582E-10
ENST00000492398	PLK3	25.48	4.4582E-10
ENST00000420576	GSPT1	25.47	4.5183E-10
ENST00000370092	IDH3G	25.46	4.5183E-10
ENST00000321926	CLIC2	25.46	4.5183E-10
ENST00000441340	PLEKHO1	25.46	4.5183E-10
ENST00000452913	CIBAR1	25.46	4.5183E-10
ENST00000457404	PPA2	25.46	4.5183E-10
ENST00000547832	TMBIM6	25.46	4.5183E-10
ENST00000617660	None	25.46	4.5183E-10
ENST00000548802	None	25.46	4.5183E-10
ENST00000400770	DTYMK	25.45	4.5670E-10
ENST00000397914	CYB5A	25.43	4.6756E-10
ENST00000484799	SMC4	25.43	4.6756E-10
ENST00000491426	PDE4DIP	25.43	4.7158E-10
ENST00000555012	ATP6V1D	25.42	4.7531E-10
ENST00000640458	None	25.42	4.7531E-10

ENST00000357146	PSMD12	25.41	5.7331E-10
ENST00000261842	AP4E1	25.41	4.8844E-10
ENST00000571688	LITAF	25.41	4.8844E-10
ENST00000397817	MPG	25.41	4.8844E-10
ENST00000396064	GTF2A2	25.41	4.8844E-10
ENST00000602011	SHKBP1	25.41	4.8844E-10
ENST00000497556	ATP13A1	25.40	6.8943E-10
ENST00000474265	FLII	25.39	4.8844E-10
ENST00000522385	PPP2CA	25.39	4.8844E-10
ENST00000640241	None	25.39	4.8844E-10
ENST00000600791	SHKBP1	25.39	4.8844E-10
ENST00000229390	SRSF9	25.39	7.0028E-10
ENST00000450808	CMPK1	25.39	4.9138E-10
ENST00000601800	FLT3LG	25.38	4.9421E-10
ENST00000168216	HSD17B10	25.38	7.0124E-10
ENST00000392531	CRIP1	25.37	5.6733E-10
ENST00000428336	TSPO	25.37	5.6733E-10
ENST00000615321	RSBN1	25.37	5.6733E-10
ENST00000591645	TPM4	25.37	7.0935E-10
ENST00000619362	None	25.36	5.0713E-10
ENST00000529543	TBRG1	25.35	5.1567E-10
ENST00000454094	ABCF1	25.34	5.1838E-10
ENST00000421608	ABCF1	25.34	5.1838E-10
ENST00000556840	MRPL52	25.34	5.1838E-10
ENST00000350697	SEC13	25.34	5.1838E-10
ENST00000521877	PIEZO1	25.33	5.2795E-10
ENST00000322428	MAF1	25.32	5.3210E-10
ENST00000597396	SHKBP1	25.32	5.3313E-10
ENST00000614868	CWC25	25.32	5.3031E-10
ENST00000519120	MRPS28	25.32	5.3031E-10
ENST00000612655	POLR2E	25.32	5.3031E-10
ENST00000437355	PSPH	25.32	5.3031E-10
ENST00000395566	MDK	25.32	7.0028E-10
ENST00000493695	SMC4	25.32	5.3124E-10
ENST00000533178	MUTYH	25.32	5.3124E-10
ENST00000555729	AHSA1	25.31	5.3313E-10
ENST00000540249	PDHA1	25.31	6.9291E-10
ENST00000308418	RNASEH2C	25.31	5.3313E-10
ENST00000395353	MVP	25.31	5.3313E-10
ENST00000600615	STRN4	25.31	5.3313E-10
ENST00000316052	EXOSC4	25.30	5.4119E-10
ENST00000479269	DNAJC19	25.30	5.4119E-10
ENST00000619573	GGNBP2	25.30	5.4119E-10
ENST00000633649	None	25.30	5.4119E-10
ENST00000493278	CALU	25.30	5.4699E-10

ENST00000532993	ARHGEF12	25.29	5.4130E-10
ENST00000617672	None	25.29	5.4130E-10
ENST00000423990	CIBAR1	25.28	5.5216E-10
ENST00000479237	SH3GLB2	25.28	5.5216E-10
ENST00000416469	SMIM19	25.27	5.7659E-10
ENST00000455667	PCBP2	25.27	5.7659E-10
ENST00000473900	NENF	25.27	5.7659E-10
ENST00000634591	SEPTIN7	25.27	5.7659E-10
ENST00000331808	DEXI	25.27	5.5607E-10
ENST00000421063	PHYHD1	25.27	5.5607E-10
ENST00000519100	PABPC1	25.27	5.5607E-10
ENST00000647721	ITPRIP	25.27	5.5607E-10
ENST00000394555	EFEMP1	25.25	5.6733E-10
ENST00000578734	RAB31	25.25	5.6733E-10
ENST00000309212	MXRA8	25.25	7.4372E-10
ENST00000369509	VANGL1	25.25	5.6733E-10
ENST00000394788	CD36	25.25	5.6733E-10
ENST00000395711	SDAD1	25.25	5.6733E-10
ENST00000667857	KMT2E	25.25	5.6733E-10
ENST00000461447	GLUL	25.24	5.7331E-10
ENST00000527158	SNRNP35	25.24	5.7331E-10
ENST00000214869	TMED1	25.24	5.7331E-10
ENST00000510207	AIMP1	25.23	5.8818E-10
ENST00000485757	RSPO3	25.23	5.8279E-10
ENST00000546840	None	25.23	5.8279E-10
ENST00000391758	TFPT	25.23	5.7331E-10
ENST00000611344	TFPT	25.23	5.7331E-10
ENST00000361036	GPAA1	25.23	7.6035E-10
ENST00000249842	ISLR	25.23	5.7623E-10
ENST00000671810	ISLR	25.23	5.7623E-10
ENST00000496672	DNTTIP2	25.22	5.7633E-10
ENST00000497666	DCTN1	25.22	5.7633E-10
ENST00000393611	RAB24	25.22	5.7633E-10
ENST00000651235	MRM2	25.22	5.7659E-10
ENST00000423617	NBDY	25.22	5.7659E-10
ENST00000463624	WDR82	25.22	5.7659E-10
ENST00000407360	RFXANK	25.22	7.7874E-10
ENST00000216479	AHSA1	25.22	5.7725E-10
ENST00000295685	ARPC2	25.22	5.7725E-10
ENST00000484646	ATRAID	25.21	5.7981E-10
ENST00000651945	UBE2W	25.21	7.7894E-10
ENST00000472168	PIEZO1	25.21	7.7894E-10
ENST00000566233	RAB11A	25.21	7.7894E-10
ENST00000658489	TPM4	25.21	7.7894E-10
ENST00000414401	CECR7	25.21	5.8115E-10

ENST00000547698	LMBR1L	25.21	5.8115E-10
ENST00000588841	C19orf53	25.21	5.8115E-10
ENST00000518157	SBSN	25.20	5.8279E-10
ENST00000578996	CDK5RAP3	25.20	5.8279E-10
ENST00000449323	RPS27A	25.20	5.8524E-10
ENST00000357179	TOP3B	25.19	5.8834E-10
ENST00000477184	RASA4B	25.19	5.8834E-10
ENST00000227520	CCDC86	25.18	6.0317E-10
ENST00000457596	UPP1	25.17	6.0318E-10
ENST00000550189	NFYB	25.17	6.0318E-10
ENST00000593786	NCOA6	25.17	6.0318E-10
ENST00000616054	CCL18	25.17	6.0579E-10
ENST00000530584	POLD4	25.17	6.0579E-10
ENST00000409203	SGO2	25.16	6.0847E-10
ENST00000480975	GNAS	25.16	6.0847E-10
ENST00000417700	RMDN2	25.16	6.1014E-10
ENST00000331803	UPP1	25.16	6.1014E-10
ENST00000425675	ISOC2	25.15	6.1292E-10
ENST00000509589	CYSTM1	25.15	6.1244E-10
ENST00000485486	FTSJ1	25.15	6.1244E-10
ENST00000492182	XPO1	25.15	6.1244E-10
ENST00000408998	WDR33	25.14	6.2784E-10
ENST00000425851	SECISBP2	25.14	6.2784E-10
ENST00000461431	CCDC80	25.14	6.2784E-10
ENST00000215587	POLR2E	25.13	7.1304E-10
ENST00000491099	EPC2	25.13	6.3112E-10
ENST00000497755	CLIC4	25.13	6.3112E-10
ENST00000529115	RNH1	25.12	6.3487E-10
ENST00000632667	None	25.12	6.3487E-10
ENST00000377488	PARK7	25.12	7.1797E-10
ENST00000520796	CLU	25.12	7.1797E-10
ENST00000612988	None	25.11	6.4232E-10
ENST00000618533	RPP40	25.11	6.4232E-10
ENST00000636766	None	25.11	6.4232E-10
ENST00000484375	ARPC1B	25.11	6.4531E-10
ENST00000586564	C19orf25	25.11	6.4531E-10
ENST00000635175	SEPTIN7	25.11	6.4531E-10
ENST00000383391	LSM2	25.10	6.4948E-10
ENST00000432122	LSM2	25.10	6.4948E-10
ENST00000434125	LSM2	25.10	6.4948E-10
ENST00000424975	LSM2	25.10	6.4948E-10
ENST00000455705	LSM2	25.10	6.4948E-10
ENST00000532047	ARL14EP	25.10	6.4948E-10
ENST00000492709	HM13	25.09	9.3925E-10
ENST00000462166	TPRKB	25.09	6.5495E-10

ENST00000517501	NUDCD2	25.09	6.5495E-10
ENST00000475534	RRP1	25.09	6.6033E-10
ENST00000474709	ADAM15	25.08	6.5879E-10
ENST00000545616	RNF24	25.08	6.6437E-10
ENST00000527163	BSDC1	25.06	6.7608E-10
ENST00000547281	CDK4	25.06	6.8064E-10
ENST00000612493	NCOA6	25.06	6.8064E-10
ENST00000346867	TAC1	25.05	6.8325E-10
ENST00000486199	PHF20L1	25.05	6.8325E-10
ENST00000505719	MRPS18C	25.05	6.8325E-10
ENST00000403050	PHF14	25.04	6.8654E-10
ENST00000467606	ITGB1BP1	25.04	6.8654E-10
ENST00000524553	CFL1	25.04	6.8654E-10
ENST00000429243	PRR13	25.04	6.8654E-10
ENST00000440277	PHB2	25.04	6.8654E-10
ENST00000554969	HNRNPC	25.04	6.8654E-10
ENST00000577906	SNRPD1	25.04	6.8654E-10
ENST00000417101	ACTB	25.04	6.9247E-10
ENST00000356714	NUDT1	25.03	6.9465E-10
ENST00000487865	NDFIP2	25.03	6.9465E-10
ENST00000460439	ANXA4	25.02	7.2194E-10
ENST00000565031	NDUFB10	25.02	7.0028E-10
ENST00000589165	ERCC1	25.02	7.0028E-10
ENST00000611509	None	25.02	7.0028E-10
ENST00000651529	LAMA4	25.02	7.0028E-10
ENST00000438045	PEX1	25.02	7.0028E-10
ENST00000510920	FAM200B	25.02	7.0028E-10
ENST00000505186	TNIP2	25.02	7.0028E-10
ENST00000554840	GNG2	25.02	7.0028E-10
ENST00000569417	MLST8	25.02	7.0028E-10
ENST00000595765	CYTH2	25.01	7.0445E-10
ENST00000487078	AGBL5	25.01	7.0524E-10
ENST00000636836	UROD	25.01	7.0524E-10
ENST00000648915	EIF2B5	25.00	7.4372E-10
ENST00000517946	CAAP1	25.00	7.4372E-10
ENST00000551823	CHURC1-FNTB	25.00	7.4372E-10
ENST00000620897	CYREN	25.00	1.0490E-09
ENST00000424167	MDH2	25.00	7.1304E-10
ENST00000443409	DGCR6L	25.00	7.1304E-10
ENST00000495224	RBM34	25.00	7.1304E-10
ENST00000462686	NMNAT1	24.99	7.1709E-10
ENST00000521693	LAMA4	24.99	7.8487E-10
ENST00000366676	None	24.99	7.1950E-10
ENST00000576353	SNF8	24.99	7.1950E-10
ENST00000369721	ATP5PB	24.98	1.0775E-09

ENST00000315144	FLAD1	24.97	7.3312E-10
ENST00000519511	STC2	24.97	7.3312E-10
ENST00000520337	CTHRC1	24.97	7.3656E-10
ENST00000216037	XBP1	24.97	7.3656E-10
ENST00000457309	YWHAZ	24.97	1.0897E-09
ENST00000585900	HMG20B	24.96	7.4372E-10
ENST00000268676	DEF8	24.95	7.4372E-10
ENST00000402580	XRCC6	24.95	7.4372E-10
ENST00000461349	C9orf78	24.95	7.4372E-10
ENST00000470181	DPM2	24.95	7.4372E-10
ENST00000618135	TSEN34	24.95	7.4372E-10
ENST00000612393	TSEN34	24.95	7.4372E-10
ENST00000226105	RANGRF	24.94	1.1143E-09
ENST00000307712	SEC23A	24.94	1.1143E-09
ENST00000425441	CCDC12	24.94	7.5731E-10
ENST00000514089	SNF8	24.94	7.5731E-10
ENST00000571476	DERL2	24.94	7.5731E-10
ENST00000259632	DCTN3	24.93	1.1255E-09
ENST00000557126	CAMTA1	24.93	7.6350E-10
ENST00000560458	BNIP2	24.93	7.6350E-10
ENST00000312475	MRPL46	24.92	7.6598E-10
ENST00000477948	NTNG1	24.92	7.6598E-10
ENST00000515770	DEK	24.92	7.6598E-10
ENST00000634938	MADD	24.92	7.6598E-10
ENST00000424358	MMP24OS	24.92	7.6872E-10
ENST00000463292	DMKN	24.92	7.6872E-10
ENST00000493959	SENP6	24.92	7.6872E-10
ENST00000520484	ENDOV	24.92	7.6872E-10
ENST00000331728	LIMK2	24.92	8.0250E-10
ENST00000458600	SLC25A17	24.92	8.0250E-10
ENST00000474315	IDH3B	24.92	8.0250E-10
ENST00000540558	RHBDL2	24.92	8.0250E-10
ENST00000567887	MACF1	24.90	7.8069E-10
ENST00000361611	PSMB5	24.89	8.0250E-10
ENST00000510500	CAST	24.89	8.0250E-10
ENST00000556322	LGALS3	24.89	8.0250E-10
ENST00000372542	SNX21	24.88	8.0250E-10
ENST00000483859	SENP6	24.88	8.0250E-10
ENST00000396394	PSMA1	24.88	8.0654E-10
ENST00000540962	PSMD9	24.88	8.0654E-10
ENST00000551396	MRPL42	24.88	8.0654E-10
ENST00000436900	DAZAP2	24.87	1.0173E-09
ENST00000541873	RFXANK	24.86	9.8285E-10
ENST00000543227	ZNF611	24.86	9.8285E-10
ENST00000571971	DERL2	24.86	9.8285E-10

ENST00000586242	GRN	24.86	9.8285E-10
ENST00000539687	SPARC	24.86	8.1889E-10
ENST00000548118	ATF7	24.86	8.1889E-10
ENST00000558644	AKAP13	24.86	8.1889E-10
ENST00000507614	TIMM8B	24.85	9.9914E-10
ENST00000563415	CDIPT	24.85	8.2471E-10
ENST00000589686	CIRBP	24.85	8.4124E-10
ENST00000412806	SH3BP5	24.85	1.2301E-09
ENST00000391918	CALM3	24.84	8.3412E-10
ENST00000484501	IL20RB	24.84	8.3412E-10
ENST00000409332	UBE2F	24.84	8.3412E-10
ENST00000477172	MAD1L1	24.84	8.3412E-10
ENST00000596605	CLPP	24.84	8.3412E-10
ENST00000538217	TMBIM4	24.84	8.3988E-10
ENST00000542103	None	24.83	8.5646E-10
ENST00000326092	RPL21	24.83	1.2502E-09
ENST00000427685	C19orf25	24.83	1.0395E-09
ENST00000252029	TYMP	24.82	8.5130E-10
ENST00000367439	GLRX2	24.82	1.2661E-09
ENST00000637551	None	24.81	8.6208E-10
ENST00000652485	RAD50	24.81	8.6208E-10
ENST00000633401	AATF	24.81	8.8838E-10
ENST00000423591	FAM120AOS	24.81	8.8838E-10
ENST00000610798	AATF	24.81	8.8838E-10
ENST00000498107	SSBP1	24.80	8.7493E-10
ENST00000359889	FAM3A	24.80	8.7972E-10
ENST00000468427	MPHOSPH10	24.80	8.7972E-10
ENST00000508136	SLC9B2	24.80	8.7972E-10
ENST00000376228	HLA-C	24.79	1.2909E-09
ENST00000443875	C1S	24.79	1.2909E-09
ENST00000374922	NBDY	24.79	8.8551E-10
ENST00000565910	ETFA	24.79	8.8551E-10
ENST00000445033	RBMS3	24.78	8.8838E-10
ENST00000510019	RPS23	24.78	8.8838E-10
ENST00000581779	AMZ2	24.78	8.8838E-10
ENST00000507479	ANKRD37	24.78	8.9149E-10
ENST00000419040	CASP8AP2	24.78	8.9149E-10
ENST00000672165	CASP8AP2	24.78	8.9149E-10
ENST00000489992	FLAD1	24.78	8.9149E-10
ENST00000537126	MLF2	24.78	8.9149E-10
ENST00000630695	UBE2A	24.78	8.9149E-10
ENST00000613394	None	24.77	8.9871E-10
ENST00000535336	MFAP5	24.76	9.0536E-10
ENST00000620645	None	24.76	9.0536E-10
ENST00000465325	RPL14	24.76	9.1218E-10

ENST00000521330	ENDOV	24.76	9.1218E-10
ENST00000498481	ADAM15	24.75	9.1831E-10
ENST00000619610	U2AF1L5	24.75	9.1831E-10
ENST00000650659	RIT1	24.75	9.1831E-10
ENST00000439082	AKR1C3	24.74	9.2607E-10
ENST00000395633	ATP5F1D	24.74	1.1288E-09
ENST00000414897	TRIM22	24.74	1.1288E-09
ENST00000517789	POLR3D	24.74	1.3714E-09
ENST00000496993	ACBD6	24.74	9.3220E-10
ENST00000517760	C8orf76	24.74	9.3220E-10
ENST00000538118	BCAT1	24.74	9.3220E-10
ENST00000639886	HLA-Cw	24.73	9.3925E-10
ENST00000338401	METTL26	24.72	9.4589E-10
ENST00000245923	RTN2	24.72	9.4589E-10
ENST00000641834	C19orf48	24.72	9.5026E-10
ENST00000453394	RAD50	24.72	9.5026E-10
ENST00000479376	IDH3B	24.72	9.5026E-10
ENST00000589669	YJU2B	24.72	9.5026E-10
ENST00000642928	CD59	24.72	9.5026E-10
ENST00000485106	AOX1	24.68	1.4678E-09
ENST00000579374	RPL19	24.68	1.4678E-09
ENST00000444946	SOD2	24.64	1.0509E-09
ENST00000452789	TMEM134	24.64	1.0509E-09
ENST00000509153	DCAF4	24.63	1.0701E-09
ENST00000347699	MAP4K4	24.62	1.0775E-09
ENST00000553418	PNP	24.62	1.0775E-09
ENST00000529984	MUTYH	24.61	1.0848E-09
ENST00000613627	BSG	24.61	1.0848E-09
ENST00000622871	None	24.61	1.0848E-09
ENST00000559880	SLTM	24.61	1.6244E-09
ENST00000672804	HEL-S-133P	24.60	1.0912E-09
ENST00000543445	LDHA	24.60	1.0912E-09
ENST00000546677	TMEM19	24.60	1.0912E-09
ENST00000400175	MYL12B	24.60	1.0988E-09
ENST00000422436	FAP	24.60	1.0988E-09
ENST00000472258	NKTR	24.60	1.0988E-09
ENST00000338946	PHF8	24.59	1.1088E-09
ENST00000597212	DMKN	24.59	1.1096E-09
ENST00000438031	TMED2	24.59	1.1096E-09
ENST00000418261	DMKN	24.58	1.6985E-09
ENST00000559436	ZHX3	24.58	1.1230E-09
ENST00000614796	HSF1	24.58	1.1230E-09
ENST00000643099	None	24.58	1.1230E-09
ENST00000429258	CDC123	24.57	1.1255E-09
ENST00000480432	RFC2	24.57	1.1255E-09

ENST00000588826	RAD23A	24.57	1.1255E-09
ENST00000391751	RPS9	24.56	1.1292E-09
ENST00000420206	None	24.56	1.1292E-09
ENST00000459956	RPS19BP1	24.56	1.1292E-09
ENST00000475590	RBM5	24.56	1.1292E-09
ENST00000551694	FKBP11	24.56	1.1292E-09
ENST00000569234	TERF2IP	24.56	1.1292E-09
ENST00000615167	None	24.56	1.1292E-09
ENST00000360786	CXNM	24.55	1.1463E-09
ENST00000530463	ZNF143	24.55	1.1463E-09
ENST00000546670	DCTN2	24.55	1.1463E-09
ENST00000360947	ZNF219	24.55	1.1463E-09
ENST00000546795	TMEM19	24.54	1.1560E-09
ENST00000551117	METTL1	24.54	1.1560E-09
ENST00000598840	SAE1	24.54	1.1681E-09
ENST00000360579	VPS29	24.53	1.7969E-09
ENST00000444620	ARID4B	24.53	1.7969E-09
ENST00000468534	CCDC88A	24.53	1.7969E-09
ENST00000620540	None	24.53	1.7969E-09
ENST00000584514	CENPX	24.53	1.1799E-09
ENST00000461018	CNN3	24.52	1.1887E-09
ENST00000476174	AZI2	24.52	1.1887E-09
ENST00000331285	PCYT2	24.52	1.1887E-09
ENST00000300850	ZNF646	24.52	1.1887E-09
ENST00000245552	NT5C	24.52	1.8366E-09
ENST00000524854	RIC8A	24.51	1.2004E-09
ENST00000465139	ANP32A	24.51	1.2070E-09
ENST00000583037	HYI	24.51	1.2070E-09
ENST00000370066	NTNG1	24.51	1.2070E-09
ENST00000400907	RERE	24.51	1.2070E-09
ENST00000520382	ATOX1	24.51	1.2070E-09
ENST00000611584	None	24.51	1.2070E-09
ENST00000474939	PRKAB2	24.50	1.2176E-09
ENST00000540564	SOAT1	24.50	1.2176E-09
ENST00000542255	ACADVL	24.48	1.2408E-09
ENST00000426395	FCGRT	24.48	1.2504E-09
ENST00000543175	PTRH1	24.48	1.2504E-09
ENST00000505307	UBE2D3	24.47	1.2602E-09
ENST00000515836	SDAD1	24.47	1.2602E-09
ENST00000544649	CCDC91	24.47	1.2602E-09
ENST00000508495	HINT1	24.46	1.2686E-09
ENST00000543891	TMEM183A	24.46	1.2686E-09
ENST00000553204	LMBR1L	24.46	1.2686E-09
ENST00000555414	APEX1	24.45	1.2760E-09
ENST00000164227	BCL3	24.45	1.2760E-09

ENST00000535296	SLC3A2	24.45	1.2760E-09
ENST00000652287	UROD	24.45	1.2760E-09
ENST00000565975	SPIN1	24.45	1.2760E-09
ENST00000426679	ISCA1P1	24.44	1.2909E-09
ENST00000355082	EIF3I	24.44	1.2909E-09
ENST00000408952	None	24.44	1.2909E-09
ENST00000460950	PRDX6	24.44	1.2909E-09
ENST00000468085	DUSP10	24.44	1.2909E-09
ENST00000498549	DENND10	24.44	1.2909E-09
ENST00000585518	HAUS1	24.44	1.2909E-09
ENST00000622665	CWC25	24.44	1.2909E-09
ENST00000632717	None	24.44	1.2909E-09
ENST00000302422	TMEM256	24.44	1.2909E-09
ENST00000639136	TMEM256	24.44	1.2909E-09
ENST00000396822	SNAI2	24.44	1.2909E-09
ENST00000409773	DYNC1I2	24.44	1.2909E-09
ENST00000489022	AKR1B1	24.44	1.2909E-09
ENST00000618773	UCHL3	24.43	2.0103E-09
ENST00000479362	CDK11A	24.42	1.3094E-09
ENST00000481274	IMPDH2	24.42	1.3094E-09
ENST00000507753	ANKRD37	24.42	1.3094E-09
ENST00000542638	ELOVL5	24.42	1.3094E-09
ENST00000607471	MED16	24.42	1.3094E-09
ENST00000641939	PHGDH	24.42	1.3094E-09
ENST00000371460	SUPT3H	24.41	1.3342E-09
ENST00000493454	NAA50	24.40	1.3481E-09
ENST00000636843	CTSD	24.40	1.3481E-09
ENST00000376027	TEX30	24.39	1.3620E-09
ENST00000483896	RRP1	24.39	1.3620E-09
ENST00000597202	PNPLA6	24.39	1.3620E-09
ENST00000541426	ETV6	24.38	1.3766E-09
ENST00000511393	ANKRD37	24.38	1.3766E-09
ENST00000508361	TARS1	24.38	1.3766E-09
ENST00000619603	None	24.37	2.4170E-09
ENST00000461311	CAMTA1	24.36	1.4417E-09
ENST00000507396	SDAD1	24.36	1.4417E-09
ENST00000530860	DCPS	24.17	2.8599E-09
ENST00000243213	IL13RA2	24.16	2.9615E-09
ENST00000296126	WDR43	24.16	2.9615E-09
ENST00000361682	COMT	23.94	3.8728E-09
ENST00000508573	SMARCA5	23.94	3.8728E-09
ENST00000514300	QDPR	23.94	3.8728E-09
ENST00000361891	ILF2	23.61	6.4016E-09
ENST00000338338	AURKAIP1	23.41	8.2861E-09
ENST00000457215	ITM2C	23.25	1.0136E-08

ENST00000467351	RASA4B	23.25	1.0136E-08
ENST00000523589	CLU	23.25	1.0136E-08
ENST00000582773	TMEM104	23.25	1.0136E-08
ENST00000389463	LAMA4	23.25	1.0193E-08
ENST00000532375	TSPAN4	23.25	1.0193E-08
ENST00000412505	ARL6IP4	23.24	1.0228E-08
ENST00000446821	ACTR3	23.24	1.0228E-08
ENST00000591695	TMED1	23.24	1.0228E-08
ENST00000520648	STC2	23.24	1.0228E-08
ENST00000494549	ACTR1A	23.24	1.0270E-08
ENST00000565205	TRAPPC2L	23.24	1.0270E-08
ENST00000598544	NOSIP	23.24	1.0270E-08
ENST00000356508	PLD3	23.23	1.0312E-08
ENST00000442970	NKTR	23.23	1.0312E-08
ENST00000527906	PSMC3	23.23	1.0312E-08
ENST00000359551	SHARPIN	23.23	1.0363E-08
ENST00000474052	PHYKPL	23.23	1.0363E-08
ENST00000235958	HMGCL	23.22	1.0409E-08
ENST00000411695	ST13	23.22	1.0409E-08
ENST00000413566	EXOC5	23.22	1.0409E-08
ENST00000583921	RAB31	23.21	1.0458E-08
ENST00000600697	ZNF749	23.21	1.0458E-08
ENST00000265260	PCNP	23.21	1.0458E-08
ENST00000423467	TENT5A	23.21	1.0511E-08
ENST00000445842	NKTR	23.21	1.0511E-08
ENST00000486653	UBE2V1	23.21	1.0511E-08
ENST00000463880	ARF4	23.20	1.0552E-08
ENST00000524427	FEZ1	23.20	1.0552E-08
ENST00000593149	EIF3K	23.20	1.0552E-08
ENST00000593287	C19orf48	23.20	1.0552E-08
ENST00000634474	None	23.20	1.0552E-08
ENST00000369754	TENT5A	23.20	1.0609E-08
ENST00000558340	DHRS1	23.20	1.0609E-08
ENST00000645396	None	23.20	1.0609E-08
ENST00000450518	ACTL6A	22.96	1.4516E-08
ENST00000621783	CPNE3	22.96	1.4516E-08
ENST00000644078	CYSTM1	22.96	1.4516E-08
ENST00000393155	DCN	17.51	1.7434E-06
ENST00000555619	NPC2	16.06	9.5458E-06
ENST00000276390	ATP6V1B2	15.87	5.5114E-05
ENST00000408941	SCP2	15.82	1.0124E-04
ENST00000340852	HM13	15.63	6.2700E-05
ENST00000397721	CADPS2	15.47	2.1224E-04
ENST00000389093	PKM	15.25	4.9598E-05
ENST00000338663	SLC3A2	15.22	2.7182E-04

ENST00000442997	AKR1C1	15.18	2.8227E-04
ENST00000644362	HEL-S-68p	15.11	2.1039E-04
ENST00000464781	RBMX	15.03	4.9598E-05
ENST00000303436	ARF4	14.98	1.4132E-04
ENST00000413971	TOP2B	14.92	3.6638E-04
ENST00000230340	BYSL	14.82	4.0539E-04
ENST00000656419	GNAS	14.76	2.0489E-04
ENST00000478000	ADAP1	14.72	4.3271E-05
ENST00000315423	TNIP2	14.71	1.3439E-04
ENST00000611080	None	14.71	4.5006E-04
ENST00000561457	TMEM250	14.70	1.5689E-04
ENST00000358406	GSTK1	14.68	3.7186E-04
ENST00000417189	NRP2	14.62	3.9488E-04
ENST00000327892	TUBB	14.51	1.2015E-04
ENST00000518857	ATP6V1C1	14.50	1.7354E-04
ENST00000495447	MGST3	14.49	1.6761E-04
ENST00000449428	MXRA7	14.45	2.4952E-05
ENST00000617716	LOC102724159	14.45	8.1174E-04
ENST00000645908	RPL5	14.45	2.1868E-04
ENST00000535090	RAN	14.44	2.1224E-04
ENST00000464916	COL1A2	14.37	3.2665E-04
ENST00000351018	ARHG	14.36	6.3038E-04
ENST00000291386	SSU72	14.34	6.3799E-04
ENST00000281938	HSPB8	14.33	2.2327E-04
ENST00000393128	MRPL42	14.30	5.3736E-04
ENST00000531329	EIF3F	14.28	5.4467E-04
ENST00000431664	SAR1A	14.25	4.9752E-04
ENST00000644020	PMP22	14.24	5.6716E-04
ENST00000551003	PRR13	14.18	2.3599E-04
ENST00000301012	MVD	14.17	7.5023E-04
ENST00000246802	NOP53	14.17	1.9808E-04
ENST00000388825	GPX3	14.15	1.3029E-04
ENST00000348124	TRAF6	14.14	1.0789E-03
ENST00000222005	CDC37	14.13	1.0914E-03
ENST00000405140	CLU	14.10	9.9973E-04
ENST00000265857	GET4	14.08	8.2266E-04
ENST00000550777	METAP2	14.07	8.2487E-04
ENST00000340607	PTGES	14.07	1.3285E-04
ENST00000355653	VAT1	14.06	2.1647E-04
ENST00000381187	CD99	14.05	2.0321E-04
ENST00000370435	OGFRL1	14.02	7.0067E-04
ENST00000217233	TRIB3	13.96	3.0568E-05
ENST00000519004	PABPC1	13.96	4.2089E-04
ENST00000285908	BOD1	13.92	2.3169E-04
ENST00000258405	SERPINE2	13.89	2.7450E-04

ENST00000520687	SPARC	13.88	1.3631E-03
ENST00000378470	FAM107B	13.88	1.2241E-03
ENST00000240333	SLC35B1	13.86	3.7186E-04
ENST00000410113	EVA1A	13.77	1.0906E-03
ENST00000482600	EIF3L	13.77	3.4473E-04
ENST00000000233	ARF5	13.77	1.0947E-03
ENST00000317897	MAGEF1	13.76	4.1039E-04
ENST00000475650	TBC1D10B	13.76	1.5237E-03
ENST00000400531	TUBB	13.74	7.4761E-04
ENST00000396059	UBE2V1	13.73	4.0960E-04
ENST00000525900	FDFT1	13.68	3.6982E-04
ENST00000229563	TMEM14C	13.67	4.0947E-04
ENST00000643226	None	13.67	4.0947E-04
ENST00000552962	DCN	13.67	3.7030E-04
ENST00000572383	PFN1	13.66	5.3048E-04
ENST00000360840	MRPL37	13.65	9.2971E-05
ENST00000292314	CCDC12	13.65	1.6799E-03
ENST00000619426	PSMB3	13.63	4.3103E-04
ENST00000343528	PDXK	13.58	5.4185E-04
ENST00000611014	None	13.58	4.9465E-04
ENST00000652253	NKAP	13.56	4.7371E-04
ENST00000584008	PSMD12	13.56	1.8100E-03
ENST00000301522	PRDX2	13.55	3.0902E-04
ENST00000470017	PRDX6	13.54	5.2761E-04
ENST00000611688	None	13.51	5.0825E-04
ENST00000295006	CAPN2	13.50	2.1559E-03
ENST00000334678	RPS19BP1	13.41	9.6826E-04
ENST00000622702	None	13.40	5.2709E-04
ENST00000395044	VDAC1	13.39	1.5440E-03
ENST00000635671	EFEMP1	13.36	3.6220E-04
ENST00000276096	EBP	13.32	1.6515E-03
ENST00000368687	CHTOP	13.31	1.1284E-03
ENST00000350021	NDUFB6	13.30	1.0168E-03
ENST00000481073	XPO1	13.30	1.9514E-03
ENST00000611428	CD99	13.28	6.7110E-04
ENST00000425762	PSMB5	13.24	5.8790E-04
ENST00000296289	None	13.19	1.5324E-03
ENST00000303904	COPS6	13.18	2.5121E-03
ENST00000601655	RPS16	13.18	5.5977E-04
ENST00000368884	PSMD4	13.14	2.9402E-03
ENST00000196061	PLOD1	13.13	2.3537E-03
ENST00000202834	TMEM230	13.13	1.9591E-03
ENST00000471273	TBC1D23	13.11	3.0028E-03
ENST00000543462	VEGFB	13.11	6.9710E-04
ENST00000267812	MFAP1	13.07	2.4753E-03

ENST00000470074	TANC1	13.07	2.5121E-03
ENST00000264748	FGFRL1	13.06	1.7143E-03
ENST00000537814	SLC35B2	13.06	2.0828E-03
ENST00000390687	SNRPN	13.06	2.5354E-03
ENST00000267996	TPM1	13.04	1.1600E-03
ENST00000288986	NCK1	13.04	2.8427E-03
ENST00000418939	PPP1R2	13.03	2.8534E-03
ENST00000361439	NSMCE1	13.03	1.3170E-03
ENST00000380539	SERPINB6	13.02	6.6199E-04
ENST00000535217	CCDC86	13.02	2.8851E-03
ENST00000272167	EPHX1	13.00	1.4161E-03
ENST00000367885	MGST3	13.00	9.0606E-04
ENST00000288439	SLC38A10	13.00	2.1939E-03
ENST00000601220	CHMP2A	12.98	6.3153E-04
ENST00000614502	PDLIM2	12.96	8.5227E-04
ENST00000486823	GSTM3	12.94	8.1059E-04
ENST00000542928	COPS2	12.92	2.3574E-03
ENST00000362091	FBH1	12.88	1.3483E-03
ENST00000549164	ATP5MC2	12.87	1.0209E-03
ENST00000257013	RTL8C	12.86	1.3439E-04
ENST00000206020	SPAG7	12.86	1.0396E-03
ENST00000444683	HLA-E	12.85	1.4345E-03
ENST00000425603	HLA-E	12.85	1.4345E-03
ENST00000498411	FLNA	12.84	1.7347E-03
ENST00000256689	SLC38A2	12.83	2.3683E-04
ENST00000368010	PFDN2	12.83	7.6185E-04
ENST00000269080	ABCA8	12.82	3.0557E-03
ENST00000440930	None	12.82	1.4297E-03
ENST00000361901	CALD1	12.79	1.4182E-03
ENST00000637215	CELF2	12.78	3.5258E-03
ENST00000381192	CD99	12.76	2.1039E-04
ENST00000490426	ITGB1BP1	12.76	1.0410E-03
ENST00000426447	PDAP1	12.76	1.0041E-03
ENST00000379852	NRBP1	12.75	4.0368E-03
ENST00000554455	HNRNPC	12.74	3.6151E-03
ENST00000403564	RPS7	12.74	1.2131E-03
ENST00000392826	SLC4A2	12.74	1.0299E-03
ENST00000436648	GLYR1	12.73	3.3073E-03
ENST00000370010	SENP6	12.69	3.4462E-03
ENST00000536503	MED21	12.68	1.1615E-03
ENST00000648919	ERLIN2	12.67	3.8305E-03
ENST00000460450	TTL	12.67	3.8462E-03
ENST00000297109	SAP30L	12.65	3.3374E-03
ENST00000272902	SUMF1	12.64	3.5514E-03
ENST00000328434	COA3	12.63	3.9544E-03

ENST00000393470	CAV1	12.63	3.0100E-03
ENST00000371614	KTI12	12.63	3.5829E-03
ENST00000282032	ARL14EP	12.63	1.6772E-03
ENST00000263774	NDUFS3	12.60	3.0836E-03
ENST00000645714	None	12.60	3.0836E-03
ENST00000330579	NOC4L	12.60	4.0532E-03
ENST00000672504	NOC4L	12.60	4.0532E-03
ENST00000425642	ATP6V0E2	12.60	4.0701E-03
ENST00000460720	ITGB1BP1	12.59	9.5678E-04
ENST00000645300	RPL5	12.59	4.5529E-03
ENST00000370247	BCAR3	12.59	3.7115E-03
ENST00000275036	HMGN3	12.59	3.1263E-03
ENST00000553995	CALM1	12.56	1.2201E-03
ENST00000255764	MED10	12.54	3.8919E-03
ENST00000509282	GLRB	12.54	4.2671E-03
ENST00000511832	PHB	12.52	2.7732E-03
ENST00000451378	NDUFAF3	12.52	1.5145E-03
ENST00000329970	GTF3C6	12.50	4.8622E-03
ENST00000503501	SENP6	12.49	3.4054E-03
ENST00000479853	SDHAF3	12.48	4.4449E-03
ENST00000414083	DAXX	12.48	4.4455E-03
ENST00000531743	FAU	12.48	1.5776E-03
ENST00000369937	CUEDC2	12.47	4.1041E-03
ENST00000264335	HEL2	12.47	2.0398E-03
ENST00000379715	EEF1E1	12.42	4.2993E-03
ENST00000620073	HSP90AB1	12.41	1.2893E-03
ENST00000546323	MED21	12.39	1.7328E-03
ENST00000375499	SDHB	12.39	4.3437E-03
ENST00000590161	PALM	12.39	5.3513E-03
ENST00000465242	POLE4	12.38	1.2292E-03
ENST00000555085	TMED10	12.37	3.1482E-03
ENST00000561006	PPP2R5C	12.37	2.8585E-03
ENST00000487010	COL3A1	12.37	4.2773E-03
ENST00000376499	TLE1	12.37	4.4154E-03
ENST00000491229	PSMD1	12.36	4.8746E-03
ENST00000504492	MAP1B	12.36	4.8746E-03
ENST00000319285	DCTPP1	12.35	4.5375E-03
ENST00000470701	POLR2F	12.34	1.7414E-03
ENST00000613102	GGNBP2	12.34	4.5539E-03
ENST00000450592	NUSAP1	12.33	3.8811E-03
ENST00000399272	RCAN1	12.33	4.4734E-03
ENST00000614574	C8orf58	12.29	5.7720E-03
ENST00000366466	NDUFB6	12.29	2.7047E-03
ENST00000376582	TIMM17B	12.29	1.3149E-03
ENST00000260952	ASNSD1	12.29	4.7208E-03

ENST00000527449	RBM25	12.29	4.7634E-03
ENST00000447927	EIF6	12.27	4.7928E-03
ENST00000624234	EIF3L	12.27	2.1935E-03
ENST00000199320	DIMT1	12.27	4.7820E-03
ENST00000550515	ESYT1	12.27	5.2731E-03
ENST00000004982	HSPB6	12.26	1.5786E-03
ENST00000497078	RPS18	12.26	3.4762E-03
ENST00000413140	ACP1	12.26	2.1361E-03
ENST00000300086	TERF2IP	12.25	2.7557E-04
ENST00000356064	ARFIP1	12.22	4.2290E-03
ENST00000572517	SERPINF1	12.21	5.0334E-03
ENST00000401959	SNU13	12.21	6.0886E-03
ENST00000295491	MRPS18C	12.20	2.2104E-03
ENST00000269195	GALNT1	12.19	4.8283E-03
ENST00000350028	SAMM50	12.19	5.1297E-03
ENST00000250454	EAPP	12.19	6.2504E-03
ENST00000476292	SMYD4	12.18	5.6647E-03
ENST00000409764	CYCS	12.18	4.3795E-03
ENST00000360472	PEA15	12.17	4.3476E-04
ENST00000372520	None	12.16	1.6899E-03
ENST00000304636	COL3A1	12.16	5.0792E-04
ENST00000412716	DAZAP2	12.16	6.3890E-03
ENST00000551727	NFYB	12.16	1.9259E-03
ENST00000409658	WDR33	12.16	5.2737E-03
ENST00000637771	FGL2	12.16	5.7619E-03
ENST00000522521	MRPL15	12.16	4.4455E-03
ENST00000358402	MAX	12.16	3.0369E-03
ENST00000328879	KLHL22	12.15	4.9747E-03
ENST00000586742	TLE5	12.14	2.0386E-03
ENST00000378764	OPTN	12.14	5.2954E-03
ENST00000361298	BEX3	12.12	1.0372E-03
ENST00000462608	FAP	12.12	5.9515E-03
ENST00000577429	THOC1	12.10	3.9848E-03
ENST00000392367	RAN	12.09	2.2660E-03
ENST00000540225	None	12.08	1.9208E-03
ENST00000484872	IMPDH2	12.08	2.0398E-03
ENST00000587393	TLE5	12.07	2.8299E-03
ENST00000228318	SLC25A3	12.07	4.7678E-03
ENST00000534794	RSF1	12.07	1.9514E-03
ENST00000613953	TPI1	12.05	5.4121E-03
ENST00000380753	AKR1C2	12.05	5.4132E-03
ENST00000248378	EMC6	12.05	5.7750E-03
ENST00000336812	DHX15	12.04	5.8268E-03
ENST00000532986	EEF1G	12.03	3.2462E-03
ENST00000380927	SECISBP2L	12.02	5.8831E-03

ENST00000381854	CDC37L1	12.02	2.1788E-03
ENST00000020945	SNAI2	12.00	7.7856E-04
ENST00000624481	CD99	11.98	7.3629E-03
ENST00000497557	EMC3	11.97	6.6641E-03
ENST00000265523	BLVRA	11.96	7.4835E-03
ENST00000485641	UXT	11.95	3.8403E-03
ENST00000541679	RAN	11.95	7.4761E-04
ENST00000435120	MLF2	11.94	6.0620E-03
ENST00000225525	TAX1BP3	11.92	1.8741E-03
ENST00000512903	DHX15	11.90	5.8460E-04
ENST00000431232	PGAP6	11.90	6.4306E-03
ENST00000473593	PDCD6IP	11.89	4.6988E-03
ENST00000503756	None	11.89	7.1563E-03
ENST00000397375	MRPS34	11.88	6.6226E-03
ENST00000539573	NDUFA9	11.87	5.6344E-03
ENST00000514911	DIMT1	11.87	7.2607E-03
ENST00000354895	VKORC1	11.87	2.6498E-03
ENST00000439363	EIF2B3	11.86	4.3795E-03
ENST00000346213	RAB5C	11.86	1.5730E-03
ENST00000368300	LMNA	11.86	1.0452E-03
ENST00000532402	HEL-S-164nA	11.85	2.8252E-03
ENST00000300434	FAM102A	11.84	5.7767E-03
ENST00000407721	DHCR7	11.84	6.5695E-03
ENST00000522934	COX6C	11.82	2.7337E-03
ENST00000360403	EIF2B3	11.81	1.3487E-03
ENST00000373238	SAR1A	11.81	8.0748E-03
ENST00000267102	LMBR1L	11.80	7.6406E-03
ENST00000467246	HPS1	11.79	1.4918E-03
ENST00000510906	PDPN	11.78	7.7489E-03
ENST00000418115	RHOA	11.78	2.7995E-04
ENST00000343575	CXCL12	11.77	6.7762E-03
ENST00000403685	ETV1	11.77	7.8110E-03
ENST00000372836	CNPY3	11.77	5.5978E-04
ENST00000354361	SYF2	11.77	7.2350E-03
ENST00000394564	AKAP9	11.76	7.8356E-03
ENST00000441864	BCL2L12	11.76	7.8480E-03
ENST00000381858	CDC37L1	11.73	7.3629E-03
ENST00000288398	HEL-S-265	11.72	2.5275E-03
ENST00000533542	ZNF143	11.71	8.1342E-03
ENST00000322157	NPEPPS	11.71	8.1342E-03
ENST00000373491	TBC1D22B	11.70	3.0728E-03
ENST00000346166	RNF6	11.69	7.2750E-03
ENST00000471706	POLR3GL	11.69	6.4646E-03
ENST00000397370	RBM39	11.67	2.7178E-03
ENST00000449164	KRT223P	11.67	8.3855E-03

ENST00000555473	AHSA1	11.66	6.6696E-03
ENST00000503715	DEK	11.65	8.5075E-03
ENST00000622633	LITAF	11.64	7.5519E-03
ENST00000542701	RNF10	11.64	7.9439E-03
ENST00000372829	ARMCX1	11.62	7.9806E-03
ENST00000508368	DHX15	11.62	3.2616E-03
ENST00000373821	SRPK1	11.61	8.7717E-03
ENST00000611779	TAX1BP3	11.61	8.1214E-03
ENST00000409724	CAPG	11.60	8.0416E-03
ENST00000394613	PLAAT3	11.59	8.8796E-03
ENST00000360718	SQSTM1	11.59	8.8903E-03
ENST00000481383	FXR1	11.58	8.9723E-03
ENST00000272065	ACP1	11.58	3.4930E-03
ENST00000544576	MROH1	11.57	3.0532E-03
ENST00000478013	NRP2	11.56	9.0894E-03
ENST00000223369	YKT6	11.56	8.1119E-03
ENST00000307296	PDCD6IP	11.56	8.0145E-03
ENST00000262061	COPZ1	11.55	8.0134E-03
ENST00000314787	PINX1	11.54	8.5532E-03
ENST00000425375	TOM1	11.54	9.2578E-03
ENST00000552235	ZCRB1	11.53	9.0830E-04
ENST00000524628	BANF1	11.53	5.4163E-03
ENST00000266085	TIMP3	11.53	3.4387E-03
ENST00000279839	CWC15	11.53	8.1801E-03
ENST00000224784	ACTA2	11.52	8.2194E-03
ENST00000527696	RIC8A	11.52	3.9911E-03
ENST00000426877	TRAPPC5	11.51	3.4698E-03
ENST00000556218	PPP2R5C	11.49	8.8641E-03
ENST00000491291	PGK1	11.49	7.6720E-03
ENST00000409157	KCTD18	11.48	8.8641E-03
ENST00000328649	CIB1	11.48	8.4691E-03
ENST00000491011	DHX36	11.47	9.7229E-03
ENST00000531186	EIF3M	11.47	9.7683E-03
ENST00000293831	EIF4A1	11.46	3.9775E-03
ENST00000554011	EXOC5	11.45	9.0963E-03
ENST00000261220	METAP2	11.43	9.2578E-03
ENST00000477583	CACUL1	11.42	8.0463E-03
ENST00000314797	COPG1	11.42	1.3728E-03
ENST00000274063	SFRP2	11.42	1.7347E-03
ENST00000297350	TNFRSF11B	11.41	3.6578E-03
ENST00000377818	MZT1	11.38	4.0701E-03
ENST00000485527	POLE4	11.38	3.8403E-03
ENST00000413219	SDCBP	11.38	9.0894E-03
ENST00000482739	RNF187	11.38	7.1930E-03
ENST00000645627	RNF187	11.38	7.1930E-03

ENST00000304434	ELOVL5	11.37	2.6255E-03
ENST00000552141	LMBR1L	11.36	1.0555E-02
ENST00000615544	ACTG1	11.36	2.2398E-03
ENST00000661807	None	11.36	5.9656E-03
ENST00000475042	ABTB1	11.36	1.0603E-02
ENST00000328724	PPP2R5C	11.35	9.3766E-03
ENST00000513367	SRPK1	11.34	1.0742E-02
ENST00000416660	SLC51A	11.34	1.0798E-02
ENST00000663894	TPM4	11.33	8.8665E-04
ENST00000533861	TMEM179B	11.32	9.9940E-03
ENST00000548534	WASHC4	11.31	1.1024E-02
ENST00000336916	FN1	11.30	4.0515E-03
ENST00000526339	NDUFS8	11.30	4.7678E-03
ENST00000465762	NDFIP2	11.30	1.1107E-02
ENST00000452413	KCTD3	11.29	1.1156E-02
ENST00000619128	PSMD1	11.29	1.6402E-03
ENST00000476605	MICU1	11.28	1.1269E-02
ENST00000469467	SLC4A2	11.28	1.1269E-02
ENST00000339249	ZNF449	11.27	9.9672E-03
ENST00000375807	SECISBP2	11.27	9.9770E-03
ENST00000398733	UBE2D2	11.26	2.8156E-03
ENST00000335754	ACTR8	11.25	1.0347E-02
ENST00000602911	PSMD8	11.25	3.0369E-03
ENST00000358187	ITPRIP	11.24	4.0752E-03
ENST00000371482	NSMF	11.24	1.0367E-02
ENST00000406620	UBE2I	11.23	1.1631E-02
ENST00000580453	FLII	11.23	1.1631E-02
ENST00000640847	None	11.23	1.1631E-02
ENST00000572579	CHD3	11.23	9.3069E-03
ENST00000557006	TMEM229B	11.23	4.4725E-03
ENST00000299335	COX11	11.22	1.0414E-02
ENST00000421682	RBM6	11.21	1.1802E-02
ENST00000528699	SIPA1	11.21	1.1802E-02
ENST00000372270	PLAC9	11.21	1.1802E-02
ENST00000533596	KAT5	11.21	5.8085E-03
ENST00000568415	METRN	11.20	4.9342E-03
ENST00000376957	SRM	11.20	1.3043E-02
ENST00000621032	None	11.20	6.4306E-03
ENST00000647178	FDFT1	11.20	2.4072E-03
ENST00000647069	None	11.20	2.4072E-03
ENST00000493494	TRIM22	11.20	1.1948E-02
ENST00000405557	POLR2F	11.20	6.1917E-03
ENST00000400721	ARHGDIA	11.19	9.6496E-03
ENST00000536073	ARL6IP4	11.19	1.1990E-02
ENST00000573320	HGS	11.19	1.1990E-02

ENST00000586370	AP3D1	11.18	4.5044E-03
ENST00000591909	TUBB6	11.18	1.1226E-02
ENST00000630865	NCS1	11.18	1.0871E-02
ENST00000344773	BABAM2	11.17	1.0694E-02
ENST00000530026	EIF3M	11.17	9.7937E-03
ENST00000594581	STRN4	11.16	1.1292E-02
ENST00000262487	ISM1	11.16	1.1333E-02
ENST00000371761	CDKN2C	11.16	1.1043E-02
ENST00000397863	MBP	11.15	1.0968E-02
ENST00000434715	DCTN2	11.15	2.4017E-03
ENST00000504285	BDH2	11.15	1.2345E-02
ENST00000449080	CAPZA2	11.14	5.1046E-03
ENST00000508482	LUC7L3	11.12	7.2937E-03
ENST00000552403	TNS2	11.12	1.2587E-02
ENST00000223361	POLD2	11.11	1.1799E-02
ENST00000221554	YJU2B	11.11	1.1830E-02
ENST00000644855	SLC33A1	11.10	1.2789E-02
ENST00000489964	DARS1	11.10	1.1821E-02
ENST00000574151	HCFC1R1	11.10	8.2023E-03
ENST00000380647	CNTLN	11.10	1.1410E-02
ENST00000588705	None	11.09	5.6569E-03
ENST00000573216	BSG	11.07	1.0584E-02
ENST00000610495	F8A1	11.07	1.2067E-02
ENST00000453542	GPAT2	11.06	1.3132E-02
ENST00000390658	EID2	11.06	4.5949E-03
ENST00000343677	H1-2	11.06	1.0551E-02
ENST00000430715	RTN2	11.06	1.1760E-02
ENST00000548249	DCTN2	11.05	3.2402E-03
ENST00000374846	DYNLRB1	11.04	8.1214E-03
ENST00000494677	VEPH1	11.03	8.1801E-03
ENST00000376113	BIN1	11.01	4.8070E-03
ENST00000368467	PMVK	11.01	1.2048E-02
ENST00000330154	ARMCX2	11.00	2.1515E-03
ENST00000261250	C12orf4	11.00	1.2351E-02
ENST00000446643	WDR43	10.98	1.3999E-02
ENST00000311086	BOD1	10.98	2.3902E-03
ENST00000573547	DERL2	10.98	1.2992E-02
ENST00000622125	MSRB1	10.97	1.4085E-02
ENST00000299259	COPS2	10.97	7.4036E-03
ENST00000474210	MIEN1	10.94	1.2902E-02
ENST00000426396	SLC25A17	10.94	1.4403E-02
ENST00000307098	SPRR1B	10.94	1.3410E-02
ENST00000496435	AKR1B10	10.94	6.1212E-03
ENST00000404574	SMTN	10.93	1.4494E-02
ENST00000242729	SSPN	10.92	1.4542E-02

ENST00000498159	SLC66A1L	10.91	1.4628E-02
ENST00000392547	WIPF1	10.90	1.3013E-02
ENST00000420104	ARPC2	10.90	7.7220E-03
ENST00000373606	MANBAL	10.89	1.3141E-02
ENST00000469404	NCK1	10.88	1.3441E-02
ENST00000258739	KDELR2	10.88	1.3861E-02
ENST00000537888	PEX11B	10.88	1.4924E-02
ENST00000366899	DUSP10	10.88	5.3650E-03
ENST00000539242	BCAR3	10.87	7.9096E-03
ENST00000585675	C19orf25	10.87	1.5012E-02
ENST00000533482	RAD50	10.86	1.5950E-02
ENST00000311832	COMMD1	10.86	1.4158E-02
ENST00000380516	SLTM	10.86	7.1215E-03
ENST00000623876	DDIT3	10.86	7.1991E-03
ENST00000361390	MT-ND1	10.86	2.6167E-03
ENST00000476531	PGK1	10.85	1.5207E-02
ENST00000583848	ACADVL	10.85	6.6384E-03
ENST00000507756	RACK1	10.85	6.4033E-03
ENST00000521770	CLU	10.85	1.3576E-02
ENST00000381342	SNRPB	10.84	1.3843E-03
ENST00000427908	MXD3	10.84	8.7874E-03
ENST00000619387	AATF	10.84	7.1708E-03
ENST00000527525	SIPA1	10.84	1.6732E-02
ENST00000372764	PLAU	10.83	1.3755E-02
ENST00000415247	HLA-DPA1	10.83	2.8286E-02
ENST00000380554	AKR1C3	10.83	1.4859E-02
ENST00000464074	RSU1	10.83	1.5452E-02
ENST00000480865	ACAA1	10.82	1.5506E-02
ENST00000394936	PSAP	10.82	1.9358E-02
ENST00000418988	PSMA1	10.82	1.4532E-02
ENST00000590237	KEAP1	10.82	1.5606E-02
ENST00000331113	KCND2	10.81	1.4424E-02
ENST00000451562	HEL-S-69p	10.81	1.6555E-02
ENST00000493172	CAP2	10.81	1.4628E-02
ENST00000265361	SEMA3C	10.80	4.4036E-03
ENST00000492095	NECAP2	10.80	2.8770E-02
ENST00000396060	GTF2A2	10.80	4.7536E-03
ENST00000598201	SHKBP1	10.79	1.4750E-02
ENST00000483493	CAAP1	10.78	1.4905E-02
ENST00000432899	CALM2	10.78	2.8953E-02
ENST00000374888	ZXDB	10.78	1.4501E-02
ENST00000544428	ALG1	10.77	1.6086E-02
ENST00000373192	UNC5B	10.77	6.3376E-03
ENST00000173785	KLF6	10.76	1.4512E-02
ENST00000316788	AP3S1	10.76	5.6961E-03

ENST00000401042	MIER1	10.76	2.9215E-02
ENST00000621973	DDX1	10.76	1.4502E-02
ENST00000494980	CHMP2B	10.75	1.4750E-02
ENST00000565267	GSPT1	10.75	8.8384E-03
ENST00000356327	PLD1	10.73	1.5016E-02
ENST00000361557	PJA2	10.73	1.9111E-03
ENST00000434851	EIF3FP3	10.73	1.6616E-02
ENST00000347869	RBM5	10.72	1.8663E-02
ENST00000376925	CST3	10.72	1.4170E-03
ENST00000647561	NDUFV1	10.72	1.6732E-02
ENST00000395565	MDK	10.72	2.8036E-02
ENST00000472863	HOXB3	10.72	2.8036E-02
ENST00000553490	NPC2	10.72	2.8036E-02
ENST00000588458	ABCA8	10.72	2.8036E-02
ENST00000528029	TMEM262	10.72	2.8036E-02
ENST00000498614	COL6A1	10.72	3.8816E-03
ENST00000488824	GSTM3	10.71	7.6718E-03
ENST00000632612	CARS	10.71	1.8657E-02
ENST00000437136	PABPC4	10.71	1.5556E-02
ENST00000339083	RHOC	10.71	1.5287E-02
ENST00000259486	ENPP2	10.71	2.8147E-02
ENST00000498818	AKR1B10	10.71	2.8147E-02
ENST00000557022	ZFP36L1	10.71	2.8147E-02
ENST00000378364	APRT	10.71	7.0777E-03
ENST00000593945	TEAD2	10.71	2.9915E-02
ENST00000592984	HSPB6	10.70	1.5225E-02
ENST00000345057	TRADD	10.70	1.5759E-02
ENST00000336695	ROMO1	10.70	2.8286E-02
ENST00000607829	ASDURF	10.70	2.8286E-02
ENST00000420625	None	10.69	5.9782E-03
ENST00000472729	COMMD1	10.69	2.8401E-02
ENST00000261847	SECISBP2L	10.69	2.8401E-02
ENST00000348022	RBBP6	10.69	2.8401E-02
ENST00000438114	None	10.69	2.8401E-02
ENST00000566820	DEF8	10.69	2.8401E-02
ENST00000355209	POLR3H	10.69	2.8401E-02
ENST00000337566	POLR3H	10.69	9.1461E-03
ENST00000608606	None	10.68	1.7174E-02
ENST00000355674	RSL1D1	10.68	2.8492E-02
ENST00000438389	ISOC2	10.68	2.8492E-02
ENST00000544805	RNF10	10.68	2.8492E-02
ENST00000545322	RBIS	10.68	2.8492E-02
ENST00000546283	NDUFS7	10.68	2.8492E-02
ENST00000511473	RACK1	10.68	2.8492E-02
ENST00000313468	NDUFS8	10.68	1.9991E-03

ENST00000419359	KIZ	10.67	2.8650E-02
ENST00000568233	NUTF2	10.67	2.8650E-02
ENST00000414441	MEPCE	10.67	3.9586E-03
ENST00000475057	SUPT3H	10.66	1.7358E-02
ENST00000413150	MAP4K4	10.66	2.8770E-02
ENST00000560200	EIF5	10.66	2.8770E-02
ENST00000536615	OGFOD2	10.66	2.8770E-02
ENST00000522453	EIF3H	10.65	2.8893E-02
ENST00000628517	BCLAF1	10.65	2.8893E-02
ENST00000375060	MAGED2	10.65	1.5631E-02
ENST00000430372	CYREN	10.65	3.0691E-02
ENST00000648940	None	10.65	1.4532E-02
ENST00000392369	RAN	10.64	2.6722E-03
ENST00000432649	EVA1A	10.64	2.8953E-02
ENST00000475789	STEAP1	10.64	2.8953E-02
ENST00000488034	CUTA	10.64	2.8953E-02
ENST00000503306	SMUG1	10.64	2.8953E-02
ENST00000523107	ERLIN2	10.64	2.8953E-02
ENST00000565075	RAB11A	10.64	2.8953E-02
ENST00000292494	LY6E	10.64	1.6413E-02
ENST00000511338	SEC31A	10.64	1.9245E-02
ENST00000535813	TMEM134	10.63	2.9093E-02
ENST00000539074	POLD4	10.63	2.9093E-02
ENST00000641811	PHGDH	10.63	2.9093E-02
ENST00000405315	NUCB1	10.63	2.9093E-02
ENST00000327772	NDUFA12	10.63	2.3047E-03
ENST00000373703	PEF1	10.63	1.6160E-02
ENST00000460875	DHX36	10.63	1.7854E-02
ENST00000551983	HSP90B1	10.63	1.4704E-02
ENST00000380728	GPS2	10.63	3.0877E-02
ENST00000673265	None	10.63	3.0877E-02
ENST00000586339	HDAC5	10.62	2.9234E-02
ENST00000612212	SELENOW	10.62	2.9234E-02
ENST00000357997	ANKLE2	10.62	1.4621E-02
ENST00000312352	PFKM	10.62	1.2245E-03
ENST00000622144	PAMR1	10.62	8.8641E-03
ENST00000374038	RBM39	10.62	8.9723E-03
ENST00000269593	IGFBP4	10.62	4.0108E-04
ENST00000530446	HTATIP	10.61	2.9300E-02
ENST00000463190	ITGB1BP1	10.61	2.9300E-02
ENST00000478488	NKTR	10.61	2.9300E-02
ENST00000519721	FAM91A1	10.61	2.9300E-02
ENST00000534176	UBXN1	10.61	2.9300E-02
ENST00000368234	NAXE	10.61	2.9300E-02
ENST00000259457	PSMB7	10.61	1.6207E-02

ENST00000372459	CTSA	10.61	2.9409E-02
ENST00000446158	EBP	10.61	2.9409E-02
ENST00000540977	RFXANK	10.61	2.9409E-02
ENST00000392008	COPS8	10.61	3.1072E-02
ENST00000568288	UBE2I	10.60	2.9542E-02
ENST00000584027	NBPF9	10.60	2.9542E-02
ENST00000632872	PUF60	10.60	2.9542E-02
ENST00000579618	SNRPD1	10.60	2.9542E-02
ENST00000266659	GLIPR1	10.59	7.4866E-03
ENST00000610127	TXNDC12	10.59	2.9642E-02
ENST00000672656	IFT46	10.59	2.9642E-02
ENST00000343484	TCEA2	10.59	2.9642E-02
ENST00000261813	PFDN1	10.58	9.1765E-03
ENST00000551224	WASHC4	10.58	1.8476E-02
ENST00000378875	ALKBH6	10.58	2.9809E-02
ENST00000439527	UBA2	10.58	2.9809E-02
ENST00000332556	LAMP1	10.58	7.3677E-03
ENST00000581486	NCOA4	10.58	1.6409E-02
ENST00000462320	NFU1	10.57	2.0081E-02
ENST00000470529	BSCL2	10.57	2.9946E-02
ENST00000626119	COPS7A	10.57	2.9946E-02
ENST00000520813	EIF3H	10.57	2.9946E-02
ENST00000543672	DCTN2	10.57	2.9946E-02
ENST00000613082	CYP1B1	10.57	2.9946E-02
ENST00000561741	DEF8	10.57	1.8613E-02
ENST00000613570	CACYBP	10.56	3.1651E-02
ENST00000429018	GOLGA4	10.56	3.1651E-02
ENST00000409856	CCDC74A	10.56	1.8663E-02
ENST00000567671	RAB11A	10.56	1.8663E-02
ENST00000514251	ZBTB38	10.56	1.8595E-02
ENST00000471167	PRPF40A	10.56	3.0090E-02
ENST00000481580	RALY	10.56	3.0090E-02
ENST00000534515	POLD4	10.56	3.0090E-02
ENST00000476522	PRRC2C	10.56	3.0090E-02
ENST00000319041	SH3BGRL3	10.56	1.1983E-02
ENST00000317734	SLC35C2	10.55	3.0197E-02
ENST00000468372	MAD1L1	10.55	3.0197E-02
ENST00000489164	PEF1	10.55	3.0197E-02
ENST00000219789	CDIPT	10.54	3.0319E-02
ENST00000514554	TGFBI	10.54	3.0319E-02
ENST00000550482	HNRNPA1	10.54	3.0319E-02
ENST00000599833	SHKBP1	10.54	3.0319E-02
ENST00000489344	RBM3	10.53	8.8384E-03
ENST00000437412	PLAGL1	10.53	1.5732E-02
ENST00000590964	METTL23	10.53	3.0456E-02

ENST00000438997	FAM9C	10.53	3.0456E-02
ENST00000473132	TOMM20	10.53	3.0456E-02
ENST00000598555	FLT3LG	10.53	3.0456E-02
ENST00000448943	TWIST2	10.53	3.0456E-02
ENST00000671947	TWIST2	10.53	3.0456E-02
ENST00000633965	PSAP	10.52	8.0416E-03
ENST00000614381	None	10.52	3.2195E-02
ENST00000400609	ABCF1	10.52	3.0623E-02
ENST00000456791	ABCF1	10.52	3.0623E-02
ENST00000457970	ABCF1	10.52	3.0623E-02
ENST00000416932	ABCF1	10.52	3.0623E-02
ENST00000377191	XRN2	10.51	8.4017E-03
ENST00000438068	UBE2K	10.51	3.0700E-02
ENST00000466207	None	10.51	3.0700E-02
ENST00000473167	None	10.51	3.0700E-02
ENST00000480913	DDAH2	10.51	3.0700E-02
ENST00000488668	None	10.51	3.0700E-02
ENST00000490871	None	10.51	3.0700E-02
ENST00000493926	None	10.51	3.0700E-02
ENST00000494351	None	10.51	3.0700E-02
ENST00000561217	TNFAIP2	10.51	3.0700E-02
ENST00000644388	SERPINB6	10.51	3.0700E-02
ENST00000369638	RHOC	10.50	3.0852E-02
ENST00000522434	TM2D2	10.50	3.0852E-02
ENST00000396388	TSEN34	10.50	3.0852E-02
ENST00000285021	XPC	10.49	1.7517E-02
ENST00000525684	C11orf58	10.49	1.4353E-02
ENST00000246747	ARL2	10.49	3.5973E-03
ENST00000375534	MFAP2	10.49	3.0929E-02
ENST00000335171	ERLIN2	10.49	3.0929E-02
ENST00000420692	CTSB	10.49	3.0929E-02
ENST00000468006	MRPL9	10.49	3.0929E-02
ENST00000529771	NARS2	10.49	3.0929E-02
ENST00000555793	GOLGA5	10.49	3.0929E-02
ENST00000314922	PENK	10.48	1.9706E-02
ENST00000477116	COA8	10.48	3.2838E-02
ENST00000220847	PHF20L1	10.48	3.1038E-02
ENST00000343820	TGIF1	10.48	3.1038E-02
ENST00000367321	MTHFD1L	10.48	3.1038E-02
ENST00000470287	FUBP1	10.48	3.1038E-02
ENST00000588297	GLYR1	10.48	3.1038E-02
ENST00000616395	TRAPPC3	10.48	3.1038E-02
ENST00000456128	TOM1	10.48	3.1038E-02
ENST00000482434	VEPH1	10.47	1.9883E-02
ENST00000259154	KCTD3	10.47	1.7976E-02

ENST00000475894	DLGAP4	10.47	1.7991E-02
ENST00000505423	AP3S1	10.47	1.9963E-02
ENST00000231504	PPP2CA	10.47	3.1165E-02
ENST00000372492	CFAP57	10.47	3.1165E-02
ENST00000527637	CASP4LP	10.47	3.1165E-02
ENST00000625311	CAAP1	10.47	3.1165E-02
ENST00000369124	PLEKHO1	10.46	9.3492E-03
ENST00000376327	PLP2	10.46	8.6521E-03
ENST00000508658	SNX14	10.46	2.1740E-02
ENST00000458500	RPL10	10.46	1.1653E-02
ENST00000396062	FKBP3	10.46	1.8294E-02
ENST00000345896	CERS2	10.45	3.1301E-02
ENST00000381760	UCHL1	10.45	3.1301E-02
ENST00000480563	TTC28	10.45	3.1301E-02
ENST00000544637	SPX	10.45	3.1301E-02
ENST00000421109	NR2F2	10.45	3.1301E-02
ENST00000336023	TUBA1B	10.45	2.8941E-03
ENST00000574444	C1QBP	10.45	2.2192E-02
ENST00000234677	SARS1	10.45	9.1561E-03
ENST00000618908	LAP3	10.45	1.7991E-02
ENST00000443297	ACTR3	10.44	3.1439E-02
ENST00000507382	TMEM92	10.44	3.1439E-02
ENST00000533403	CD59	10.44	3.1439E-02
ENST00000646643	CLPP	10.44	3.1439E-02
ENST00000439267	BCL2L1	10.44	2.1912E-02
ENST00000528437	CASP4LP	10.43	3.1639E-02
ENST00000546672	SDSL	10.43	3.1639E-02
ENST00000462160	STK32C	10.43	3.1639E-02
ENST00000483534	UBE2V1	10.43	3.3590E-02
ENST00000367495	RAB32	10.43	9.9940E-03
ENST00000327773	GAS6	10.42	1.0306E-02
ENST00000572585	ABR	10.42	4.2773E-03
ENST00000628336	ABR	10.42	4.2773E-03
ENST00000629638	ABR	10.42	4.2773E-03
ENST00000278919	FEZ1	10.42	3.1738E-02
ENST00000478100	NCBP1	10.42	3.1738E-02
ENST00000493378	ARF4	10.42	3.1738E-02
ENST00000506521	BDH2	10.42	3.1738E-02
ENST00000456705	PWP2	10.42	3.1738E-02
ENST00000313386	RMND5B	10.42	3.1738E-02
ENST00000477661	AKR1C1	10.42	3.6220E-03
ENST00000421656	RANBP1	10.41	3.1904E-02
ENST00000435456	SLC25A17	10.41	3.1904E-02
ENST00000495168	TCEA2	10.41	3.1904E-02
ENST00000544883	NR2C2AP	10.41	3.1904E-02

ENST00000642285	DNAJC21	10.41	3.3955E-02
ENST00000371123	SMARCA1	10.41	2.2396E-02
ENST00000504279	DHX15	10.40	1.7292E-02
ENST00000509031	PPA2	10.40	3.2040E-02
ENST00000538026	SPARC	10.40	3.2040E-02
ENST00000541412	TMEM14C	10.40	3.2040E-02
ENST00000642513	TMEM14C	10.40	3.2040E-02
ENST00000428892	APOBEC3C	10.39	2.3798E-02
ENST00000547969	NAP1L1	10.39	1.9565E-02
ENST00000576553	POLR2A	10.39	2.1065E-02
ENST00000505548	UBE2D2	10.39	1.9591E-02
ENST00000431250	MTMR14	10.39	3.2181E-02
ENST00000504797	SMUG1	10.39	3.2181E-02
ENST00000525649	MTCH2	10.39	3.2181E-02
ENST00000643736	None	10.39	3.2181E-02
ENST00000216190	EIF3D	10.39	9.8002E-03
ENST00000361505	PNP	10.38	2.3368E-02
ENST00000489056	UQCRH	10.38	3.4319E-02
ENST00000508775	RNASET2	10.38	3.4319E-02
ENST00000646474	CCDC88A	10.38	3.4319E-02
ENST00000622375	None	10.38	9.6851E-03
ENST00000541630	RAN	10.38	8.9195E-04
ENST00000483713	POLR2F	10.38	3.2370E-02
ENST00000511289	ARFIP1	10.38	3.2370E-02
ENST00000523131	YWHAZ	10.38	3.2370E-02
ENST00000534113	SECISBP2	10.38	3.2370E-02
ENST00000341942	LAMTOR4	10.38	3.7610E-03
ENST00000484841	ACTB	10.37	1.4501E-02
ENST00000291294	PTGIR	10.37	2.1350E-02
ENST00000449964	RBSN	10.37	3.2589E-02
ENST00000318407	BOK	10.36	9.8188E-03
ENST00000472150	ARHGAP21	10.36	2.0035E-02
ENST00000527009	SCYL1	10.36	2.1541E-02
ENST00000392622	S100A13	10.36	3.4751E-02
ENST00000304979	PPIH	10.36	3.4751E-02
ENST00000295530	FLAD1	10.36	3.2686E-02
ENST00000316804	GRB2	10.36	3.2686E-02
ENST00000392594	THYN1	10.36	3.2686E-02
ENST00000413890	KTN1	10.36	3.2686E-02
ENST00000503326	COPB2	10.36	3.2686E-02
ENST00000527344	CFL1	10.36	3.2686E-02
ENST00000622235	SCARF2	10.36	3.2686E-02
ENST00000671868	AIMP1	10.36	3.2686E-02
ENST00000376055	BCL2L1	10.36	1.9176E-02
ENST00000541996	YTHDF2	10.36	2.1604E-02

ENST00000379751	CENPB	10.35	9.4206E-03
ENST00000439395	SPATS2L	10.35	1.8023E-02
ENST00000322569	MMP19	10.34	3.2838E-02
ENST00000311141	ZFYVE21	10.34	3.2838E-02
ENST00000436384	CD36	10.34	3.2838E-02
ENST00000531366	TMEM126A	10.34	3.2838E-02
ENST00000575605	DERL2	10.34	3.2838E-02
ENST00000583380	SKA2	10.34	3.2838E-02
ENST00000543683	NDUFB10	10.34	2.0125E-02
ENST00000472851	PEX14	10.34	2.1814E-02
ENST00000490289	UBE2V1	10.33	3.5228E-02
ENST00000538954	STOM	10.33	3.5228E-02
ENST00000419477	YWHAZ	10.33	3.3027E-02
ENST00000527718	PABPC4	10.33	3.3027E-02
ENST00000565777	MAZ	10.33	3.3027E-02
ENST00000602997	AKR1C3	10.33	3.3027E-02
ENST00000538434	EIF3K	10.33	1.2587E-02
ENST00000634857	None	10.33	1.2587E-02
ENST00000405489	ATRAID	10.32	2.3565E-02
ENST00000431534	POLR3H	10.32	3.3220E-02
ENST00000618312	MTHFD1L	10.32	1.0530E-02
ENST00000460191	DNTTIP2	10.32	2.2081E-02
ENST00000482525	RAB7A	10.32	2.0576E-02
ENST00000543369	MFAP5	10.31	3.3443E-02
ENST00000508623	QDPR	10.31	2.0764E-02
ENST00000453270	NR2F2	10.31	2.2252E-02
ENST00000340384	TUBB4B	10.30	3.5201E-03
ENST00000547869	SLC25A3	10.30	1.6377E-02
ENST00000501272	ANXA5	10.30	2.2192E-02
ENST00000578845	CDKN2A	10.30	1.0426E-02
ENST00000409181	SUMO1	10.30	3.3590E-02
ENST00000471276	TMEM198B	10.30	3.3590E-02
ENST00000487482	MAGED2	10.30	3.3590E-02
ENST00000519186	TM2D2	10.30	3.3590E-02
ENST00000564091	CLN3	10.30	3.3590E-02
ENST00000596362	CALM3	10.30	3.3590E-02
ENST00000651853	RIT1	10.30	3.3590E-02
ENST00000373292	HDLBP	10.29	1.0519E-02
ENST00000488607	ZCCHC7	10.29	3.3793E-02
ENST00000522887	EIF3E	10.29	3.3793E-02
ENST00000314475	TLCD5	10.29	3.3793E-02
ENST00000345807	CLTB	10.29	2.1912E-02
ENST00000440064	RSF1	10.28	4.2882E-03
ENST00000493562	MTMR11	10.28	2.2626E-02
ENST00000425361	MZT2B	10.27	3.3955E-02

ENST00000429220	ZPR1	10.27	3.3955E-02
ENST00000505120	CDC23	10.27	3.3955E-02
ENST00000542907	CWC15	10.27	3.3955E-02
ENST00000542923	WASHC3	10.27	3.3955E-02
ENST00000547439	APPL2	10.27	3.3955E-02
ENST00000611958	None	10.27	3.3955E-02
ENST00000467954	FTSJ1	10.27	2.2744E-02
ENST00000498522	TESK1	10.27	2.0699E-02
ENST00000397426	FBXO7	10.26	3.4145E-02
ENST00000559145	RORA	10.26	3.4145E-02
ENST00000541640	PRKAB1	10.26	3.4145E-02
ENST00000377921	RSU1	10.26	3.4145E-02
ENST00000477609	FLAD1	10.26	2.1541E-02
ENST00000492656	ADAMTS1	10.26	3.6657E-02
ENST00000602624	DDOST	10.25	1.9300E-02
ENST00000355346	ITGB1BP1	10.25	3.4319E-02
ENST00000373547	PPP6C	10.25	3.4319E-02
ENST00000419113	NDUFA3	10.25	3.4319E-02
ENST00000492991	C9orf78	10.25	3.4319E-02
ENST00000620289	None	10.25	3.4319E-02
ENST00000261811	CYSTM1	10.25	1.0354E-02
ENST00000534358	KMT2A	10.25	3.5691E-03
ENST00000529191	FTH1	10.25	1.5105E-02
ENST00000427084	SLC25A17	10.24	3.4552E-02
ENST00000488451	ITGB1BP1	10.24	3.4552E-02
ENST00000551673	YARS2	10.24	3.4552E-02
ENST00000560202	USP3	10.23	3.7020E-02
ENST00000442995	HYPK	10.23	1.4009E-02
ENST00000631496	CCL18	10.23	3.4762E-02
ENST00000470286	DYNC1I2	10.23	3.4762E-02
ENST00000496013	FBH1	10.23	3.4762E-02
ENST00000616474	CCL18	10.23	3.4762E-02
ENST00000649847	DVL3	10.23	3.4762E-02
ENST00000395850	CD59	10.22	3.0018E-03
ENST00000672586	ASL	10.22	1.1888E-02
ENST00000372556	PPCS	10.22	4.7000E-03
ENST00000490140	FDPS	10.21	4.2671E-03
ENST00000481633	MAD1L1	10.21	3.5045E-02
ENST00000413907	AEBP1	10.21	1.1967E-02
ENST00000550898	KRR1	10.21	2.2174E-02
ENST00000476847	CPB1	10.21	2.3693E-02
ENST00000479050	SLC25A20	10.21	2.3693E-02
ENST00000288532	COQ5	10.21	2.2090E-02
ENST00000313104	RPLP0	10.20	1.2231E-02
ENST00000631679	DVL1	10.20	2.5687E-02

ENST00000596702	ZNF611	10.20	3.5305E-02
ENST00000377104	SPRY2	10.20	2.1590E-02
ENST00000471426	RBMS3	10.20	8.5809E-03
ENST00000576579	SAT2	10.20	2.2359E-02
ENST00000221466	FCGRT	10.19	2.3092E-02
ENST00000564910	RAB11A	10.19	2.1665E-02
ENST00000278353	HSD17B12	10.19	3.5570E-02
ENST00000350435	UBE2D3	10.19	3.5570E-02
ENST00000559234	ZHX3	10.19	3.5570E-02
ENST00000506799	NLN	10.19	2.1875E-02
ENST00000370151	DPCD	10.18	2.6090E-02
ENST00000531323	UQCC3	10.18	3.7905E-02
ENST00000314250	TNS2	10.18	3.5812E-02
ENST00000320307	TAGLN2	10.18	3.5812E-02
ENST00000357402	MVP	10.17	1.0525E-02
ENST00000570828	CTDNEP1	10.17	1.6799E-02
ENST00000672576	CTDNEP1	10.17	1.6799E-02
ENST00000597020	AP2S1	10.16	5.1935E-03
ENST00000451553	KLHL22	10.16	2.4448E-02
ENST00000367108	DYRK3	10.16	3.5976E-02
ENST00000307145	KLF13	10.16	3.5976E-02
ENST00000434727	DPCD	10.16	3.5976E-02
ENST00000444761	MCFD2	10.16	3.5976E-02
ENST00000488731	MUTYH	10.16	3.5976E-02
ENST00000491206	RFC2	10.16	3.5976E-02
ENST00000531741	VPS26B	10.16	3.5976E-02
ENST00000672401	ACSL4	10.16	3.5976E-02
ENST00000270824	EVA1B	10.16	1.1107E-02
ENST00000462990	PPP2R1A	10.16	2.3964E-02
ENST00000462965	ARPC5	10.15	5.3364E-03
ENST00000522997	IMPA1	10.15	3.6201E-02
ENST00000453973	CAPG	10.15	2.2359E-02
ENST00000470551	DNAJC5	10.15	3.8387E-02
ENST00000617480	None	10.15	3.8387E-02
ENST00000475996	HLA-F	10.15	2.4714E-02
ENST00000220659	BRF2	10.14	1.8434E-02
ENST00000558311	WDR61	10.14	2.2434E-02
ENST00000446331	FDFT1	10.14	2.3199E-02
ENST00000528628	CRYAB	10.14	2.3199E-02
ENST00000613280	None	10.14	2.3199E-02
ENST00000597611	FKBP8	10.14	3.6484E-02
ENST00000612800	CIB1	10.13	2.2561E-02
ENST00000634600	SEPTIN7	10.13	2.3199E-02
ENST00000511526	ACAD9	10.12	1.2084E-02
ENST00000458442	TCEA2	10.12	3.6676E-02

ENST00000476103	DNAJC1	10.12	3.6676E-02
ENST00000492366	BDH2	10.12	3.6676E-02
ENST00000549106	ALDH2	10.12	3.6676E-02
ENST00000557961	MORF4L1	10.12	3.6676E-02
ENST00000590339	VPS25	10.12	3.6676E-02
ENST00000464553	SSNA1	10.12	3.6676E-02
ENST00000512621	ADH5	10.12	2.3515E-02
ENST00000316757	APBA3	10.11	2.3565E-02
ENST00000308961	NDUFA11	10.11	3.4690E-03
ENST00000344537	DTNBP1	10.11	3.6859E-02
ENST00000464275	PDLIM2	10.11	3.6859E-02
ENST00000486138	CAMTA1	10.11	3.6859E-02
ENST00000506847	SNX2	10.11	3.6859E-02
ENST00000533115	ZNRD2	10.11	3.6859E-02
ENST00000586539	ABCA8	10.11	3.6859E-02
ENST00000587902	ATP5F1A	10.11	3.6859E-02
ENST00000596386	PSMC4	10.11	3.6859E-02
ENST00000625352	None	10.11	3.6859E-02
ENST00000580018	TIMM23	10.10	2.4448E-02
ENST00000560793	CERS2	10.10	1.3013E-02
ENST00000037243	GABARAPL2	10.10	2.3693E-02
ENST00000400093	ATP5PF	10.10	3.7131E-02
ENST00000520187	CAAP1	10.10	3.7131E-02
ENST00000587735	PRELID3A	10.10	3.7131E-02
ENST00000300181	TSC22D4	10.09	2.2935E-02
ENST00000525690	RPS3	10.08	2.1814E-02
ENST00000252984	ALKBH6	10.08	3.7352E-02
ENST00000445667	None	10.08	3.7352E-02
ENST00000509769	USP53	10.08	3.7352E-02
ENST00000544702	C1RL	10.08	3.7352E-02
ENST00000614830	NOP16	10.08	3.7352E-02
ENST00000672447	None	10.08	3.7352E-02
ENST00000472127	NKTR	10.08	1.4740E-02
ENST00000543988	OTUB1	10.08	1.2587E-02
ENST00000529806	RPS2	10.07	4.2790E-03
ENST00000458159	ACTA2	10.07	2.6116E-02
ENST00000607062	ASNSD1	10.07	3.7579E-02
ENST00000361278	TBRG4	10.07	3.7579E-02
ENST00000458153	MKLN1	10.07	3.7579E-02
ENST00000262746	PRDX1	10.07	3.7579E-02
ENST00000373095	FAM102A	10.07	2.4332E-02
ENST00000393196	NME1	10.07	1.1114E-02
ENST00000282388	ZFP36L2	10.07	8.9723E-03
ENST00000317483	RAB23	10.06	1.1918E-02
ENST00000634045	None	10.06	1.5211E-02

ENST00000632449	None	10.06	3.9966E-02
ENST00000409500	GSTK1	10.05	3.7754E-02
ENST00000483770	PABPC4	10.05	3.7754E-02
ENST00000518974	PENK	10.05	3.7754E-02
ENST00000524000	FBXO32	10.05	3.7754E-02
ENST00000542279	GTF2H3	10.05	3.7754E-02
ENST00000614395	TMEM191B	10.05	3.7754E-02
ENST00000369651	NT5E	10.05	3.7754E-02
ENST00000429498	DDX59	10.05	2.8277E-02
ENST00000266458	GABARAPL1	10.05	1.4150E-02
ENST00000268603	CDH11	10.05	2.7779E-02
ENST00000368317	RSPO3	10.04	2.7859E-02
ENST00000619718	None	10.04	2.8724E-02
ENST00000367429	CFH	10.04	2.2366E-02
ENST00000396894	FTSJ1	10.04	3.8059E-02
ENST00000316615	GRB2	10.04	3.8059E-02
ENST00000401987	PPP1R7	10.04	3.8059E-02
ENST00000651325	SLC66A1L	10.04	3.8059E-02
ENST00000355426	EFEMP1	10.04	1.1152E-03
ENST00000564677	HEXA	10.03	2.0025E-02
ENST00000463472	ODF3B	10.03	4.0551E-02
ENST00000424616	None	10.03	3.8279E-02
ENST00000442966	RPP21	10.03	3.8279E-02
ENST00000515114	MCUB	10.03	3.8279E-02
ENST00000531593	GPAA1	10.03	3.8279E-02
ENST00000593646	RNASEK	10.02	4.6716E-03
ENST00000407627	ELAVL1	10.01	1.1970E-02
ENST00000460910	NKTR	10.01	3.8529E-02
ENST00000526522	RPS2	10.01	3.8529E-02
ENST00000551143	LMBR1L	10.01	3.8529E-02
ENST00000571874	ANAPC11	10.01	3.8529E-02
ENST00000579248	RPL17	10.01	3.8529E-02
ENST00000586967	CALR	10.01	7.3828E-03
ENST00000367294	ARMT1	10.00	2.7319E-02
ENST00000375549	SDHD	10.00	2.8016E-02
ENST00000395808	MED16	10.00	2.4670E-02
ENST00000436812	IGFBP2	10.00	4.1063E-02
ENST00000539672	RECQL	10.00	4.1063E-02
ENST00000570259	HSBP1	10.00	4.1063E-02
ENST00000589695	TREM1	10.00	4.1063E-02
ENST00000484464	ACP1	10.00	2.5617E-02
ENST00000396070	RPL30	10.00	3.8828E-02
ENST00000573786	OXLD1	10.00	3.8828E-02
ENST00000366987	ATF3	10.00	1.3483E-02
ENST00000509082	SDHA	9.99	1.7606E-02

ENST00000308982	ACAD9	9.98	3.9119E-02
ENST00000368299	LMNA	9.98	3.9119E-02
ENST00000496655	COPRS	9.98	3.9119E-02
ENST00000513740	SLC49A3	9.98	3.9119E-02
ENST00000519386	MRPS28	9.98	3.9119E-02
ENST00000528680	DNTTIP2	9.98	3.9119E-02
ENST00000593547	PEX11G	9.98	3.9119E-02
ENST00000622051	TSC22D1	9.98	1.5671E-02
ENST00000492245	SAP18	9.98	1.4925E-02
ENST00000368097	TAGLN2	9.98	2.1107E-03
ENST00000297268	COL1A2	9.98	1.2527E-03
ENST00000570099	YPEL3	9.97	2.9300E-02
ENST00000345436	CKLF	9.97	3.9417E-02
ENST00000412390	ZNF143	9.97	3.9417E-02
ENST00000561248	COPS2	9.97	3.9417E-02
ENST00000574322	CTDNEP1	9.97	3.9417E-02
ENST00000620044	None	9.97	3.9417E-02
ENST00000672741	None	9.97	3.9417E-02
ENST00000383434	None	9.97	4.1678E-02
ENST00000418032	GATAD2A	9.97	4.1678E-02
ENST00000453899	None	9.97	4.1678E-02
ENST00000407006	RANGRF	9.96	1.0295E-02
ENST00000469435	KLF6	9.96	2.6276E-02
ENST00000401394	CTCF	9.95	3.9622E-02
ENST00000417410	SMIM19	9.95	3.9622E-02
ENST00000549184	HEATR5A	9.95	3.9622E-02
ENST00000549966	TMBIM6	9.95	3.9622E-02
ENST00000597959	None	9.95	3.9622E-02
ENST00000380872	AKR1C1	9.95	3.6434E-03
ENST00000393580	RELT	9.95	2.9587E-02
ENST00000262193	PSMB1	9.95	1.4353E-02
ENST00000532672	BIRC2	9.94	2.9263E-02
ENST00000578809	ACADVL	9.94	7.6275E-03
ENST00000338517	SCOC	9.94	3.9890E-02
ENST00000490576	CYP1B1	9.94	3.9890E-02
ENST00000496468	TSSC4	9.94	3.9890E-02
ENST00000515687	STX18	9.94	3.9890E-02
ENST00000530485	BSDC1	9.94	3.9890E-02
ENST00000629123	None	9.94	3.9890E-02
ENST00000523688	NKD2	9.94	3.9890E-02
ENST00000483407	CSDE1	9.94	1.8294E-02
ENST00000431380	SPON2	9.93	4.2342E-02
ENST00000482168	PPCS	9.93	4.2342E-02
ENST00000567114	RPUSD1	9.93	4.2342E-02
ENST00000420712	RPL37A	9.93	1.5606E-02

ENST00000338435	GLS	9.93	2.5547E-02
ENST00000621366	KIZ	9.93	2.6598E-02
ENST00000300875	DACT3	9.93	2.6078E-02
ENST00000367114	EIF2D	9.92	4.0167E-02
ENST00000469699	MADD	9.92	4.0167E-02
ENST00000486135	MCEE	9.92	4.0167E-02
ENST00000525600	KAT5	9.92	4.0167E-02
ENST00000436160	SPCS2P4	9.92	4.0167E-02
ENST00000474253	TPI1	9.92	1.5671E-02
ENST00000284440	UCHL1	9.92	3.0289E-02
ENST00000534032	FIBP	9.92	7.3272E-03
ENST00000334743	PPP2R5C	9.92	1.9407E-02
ENST00000592388	VAT1	9.92	1.0087E-02
ENST00000340748	DNMT1	9.91	4.0491E-02
ENST00000463219	MGME1	9.91	4.0491E-02
ENST00000484412	HDLBP	9.91	4.0491E-02
ENST00000419610	RBM6	9.91	4.0491E-02
ENST00000541272	UBC	9.91	3.0197E-02
ENST00000453310	RBM39	9.90	1.8304E-02
ENST00000541738	PREPL	9.90	4.3011E-02
ENST00000442760	TPT1	9.90	8.8244E-03
ENST00000396757	CD72	9.89	4.0828E-02
ENST00000481432	NAA50	9.89	4.0828E-02
ENST00000568449	EME2	9.89	4.0828E-02
ENST00000229270	HEL-S-49	9.89	2.6254E-02
ENST00000326739	IMPDH2	9.89	1.9923E-02
ENST00000635172	Sep	9.89	2.7376E-02
ENST00000672422	EFCAB14	9.88	1.4667E-02
ENST00000425807	COMP	9.88	4.1063E-02
ENST00000470239	TFB1M	9.88	4.1063E-02
ENST00000540584	CAPRIN2	9.88	4.1063E-02
ENST00000577796	RAB31	9.88	4.1063E-02
ENST00000599701	FCGRT	9.88	4.1063E-02
ENST00000428990	RPL23AP1	9.88	4.1063E-02
ENST00000433438	RPL23AP1	9.88	4.1063E-02
ENST00000430582	RPL23AP1	9.88	4.1063E-02
ENST00000446846	RPL23AP1	9.88	4.1063E-02
ENST00000417214	RPL23AP1	9.88	4.1063E-02
ENST00000429422	MAP4	9.87	4.3793E-02
ENST00000455106	FKBP10	9.87	4.3793E-02
ENST00000493678	PARK7	9.87	4.3793E-02
ENST00000523872	PIK3R1	9.87	4.3793E-02
ENST00000580234	BRD2	9.87	2.8016E-02
ENST00000518611	BNIP3L	9.87	2.2561E-02
ENST00000553408	ATP6V1D	9.86	2.5257E-02

ENST00000622254	MAPK1IP1L	9.86	2.5486E-02
ENST00000473842	CWF19L1	9.86	4.1357E-02
ENST00000477484	SECISBP2	9.86	4.1357E-02
ENST00000483659	CAPG	9.86	4.1357E-02
ENST00000529187	TLCD5	9.86	4.1357E-02
ENST00000548870	SLC38A2	9.86	4.1357E-02
ENST00000551070	KRR1	9.86	4.1357E-02
ENST00000639141	ZNF271P	9.85	1.0849E-02
ENST00000236273	SYF2	9.85	4.3529E-03
ENST00000296444	SHISA5	9.85	2.8097E-02
ENST00000619757	HIC1	9.84	2.8147E-02
ENST00000334884	KMT2E	9.84	4.1678E-02
ENST00000281243	QDPR	9.84	4.1678E-02
ENST00000342753	MXRA8	9.84	4.1678E-02
ENST00000394908	FLOT2	9.84	4.1678E-02
ENST00000498022	NAGK	9.84	4.1678E-02
ENST00000582438	CYBC1	9.84	4.1678E-02
ENST00000470295	PPP1R35	9.84	4.1678E-02
ENST00000551946	None	9.84	4.1678E-02
ENST00000424877	KMT2C	9.83	4.4443E-02
ENST00000529464	FDFT1	9.83	4.4443E-02
ENST00000369387	H3P6	9.83	1.8984E-02
ENST00000437078	None	9.83	4.1936E-02
ENST00000479250	EIF2B5	9.83	4.1936E-02
ENST00000545033	OGFOD2	9.83	4.1936E-02
ENST00000564456	ZNF668	9.83	4.1936E-02
ENST00000643241	DNAJC19	9.83	4.1936E-02
ENST00000537590	SLC39A1	9.83	4.1936E-02
ENST00000601636	SMIM7	9.83	4.1936E-02
ENST00000451736	None	9.83	4.1936E-02
ENST00000650688	DKFZp762B153	9.82	2.8492E-02
ENST00000405123	ERLEC1	9.81	2.6309E-02
ENST00000248553	HEL-S-102	9.81	1.7494E-02
ENST00000258530	APPL2	9.81	4.2342E-02
ENST00000539759	MTCH2	9.81	4.2342E-02
ENST00000646923	MTCH2	9.81	4.2342E-02
ENST00000409077	MORN2	9.81	4.2342E-02
ENST00000478890	SRRM1	9.81	4.2342E-02
ENST00000424032	ZNF880	9.81	4.2342E-02
ENST00000394650	MCUB	9.81	2.8953E-02
ENST00000483063	POLE4	9.80	2.1517E-03
ENST00000509724	SPAG9	9.80	2.8926E-02
ENST00000392185	SNX9	9.80	3.2380E-03
ENST00000553191	CNPY2	9.80	4.5227E-02
ENST00000531921	EIF3M	9.79	4.2667E-02

ENST00000432483	PPA2	9.79	4.2667E-02
ENST00000436439	HMGCL	9.79	4.2667E-02
ENST00000521328	YWHAZ	9.79	4.2667E-02
ENST00000525045	PABPC4	9.79	4.2667E-02
ENST00000546199	SPX	9.79	4.2667E-02
ENST00000391791	PPP2R1A	9.79	2.0637E-02
ENST00000374379	CLIC4	9.79	1.2218E-02
ENST00000634597	SPATA20	9.79	3.2304E-02
ENST00000367569	SF3B5	9.78	1.7414E-03
ENST00000528095	RSF1	9.78	8.1214E-03
ENST00000395183	ARHGAP24	9.78	4.3011E-02
ENST00000488494	ITGB1	9.78	4.3011E-02
ENST00000507722	ZBTB38	9.78	4.3011E-02
ENST00000515697	FAM200B	9.78	4.3011E-02
ENST00000602679	DMKN	9.78	4.3011E-02
ENST00000648640	RPL29	9.78	4.3011E-02
ENST00000579456	CLTC	9.78	2.9396E-02
ENST00000372656	TCEAL9	9.78	3.1907E-02
ENST00000342312	ADSL	9.76	2.7348E-02
ENST00000550407	TARBP2	9.76	1.6047E-02
ENST00000449692	C11orf68	9.76	4.3392E-02
ENST00000518936	PCM1	9.76	4.3392E-02
ENST00000537289	COA4	9.76	4.3392E-02
ENST00000618074	None	9.76	4.3392E-02
ENST00000409957	PREPL	9.76	4.6152E-02
ENST00000427040	DPH5	9.76	4.6152E-02
ENST00000618169	None	9.76	2.9409E-02
ENST00000568871	FAM214A	9.75	1.6403E-02
ENST00000382456	AP2M1	9.75	2.9403E-02
ENST00000479806	DYNC1I2	9.75	2.9409E-02
ENST00000341249	METTL23	9.74	4.3793E-02
ENST00000394826	NSRP1	9.74	4.3793E-02
ENST00000489758	CLIC4	9.74	4.3793E-02
ENST00000568704	CYLD	9.74	3.2396E-02
ENST00000426086	VEGFB	9.73	5.8448E-03
ENST00000331314	DDX59	9.73	4.4128E-02
ENST00000466951	PSMB7	9.73	4.4128E-02
ENST00000510372	SNX2	9.73	4.4128E-02
ENST00000523095	ZNF395	9.73	4.4128E-02
ENST00000524114	PINX1	9.73	4.4128E-02
ENST00000527348	BANF1	9.73	4.4128E-02
ENST00000537016	TRMT10B	9.73	4.4128E-02
ENST00000559113	ANXA2	9.73	4.4128E-02
ENST00000550201	DCTN2	9.72	3.2686E-02
ENST00000531115	FIBP	9.72	4.6954E-02

ENST00000400090	ATP5PF	9.72	2.9300E-02
ENST00000356537	CCDC25	9.71	4.4443E-02
ENST00000415680	TRIM24	9.71	4.4443E-02
ENST00000453466	PMPCB	9.71	4.4443E-02
ENST00000613856	SLIRP	9.71	4.4443E-02
ENST00000616539	NKD2	9.70	3.0197E-02
ENST00000290902	SPON2	9.70	3.1849E-02
ENST00000262225	TMED2	9.70	1.7163E-02
ENST00000443816	FN1	9.70	2.0537E-02
ENST00000409321	PTMA	9.69	2.0272E-02
ENST00000253110	SHFL	9.69	3.3085E-02
ENST00000622695	LINC00869	9.69	4.4773E-02
ENST00000343524	ATG4A	9.69	4.4773E-02
ENST00000414866	DMKN	9.69	4.4773E-02
ENST00000450796	NFU1	9.69	4.4773E-02
ENST00000457805	PTP4A2	9.69	4.4773E-02
ENST00000559706	GTF2A2	9.69	4.4773E-02
ENST00000566416	VAC14	9.69	4.4773E-02
ENST00000574447	HSD3B7	9.69	4.4773E-02
ENST00000460638	EIF3L	9.69	9.5788E-03
ENST00000551475	CNPY2	9.68	4.7812E-02
ENST00000354216	UCKL1	9.68	4.7812E-02
ENST00000477649	VMA21	9.68	4.7812E-02
ENST00000527950	CRYAB	9.68	2.9642E-02
ENST00000461065	AGAP3	9.68	2.9580E-02
ENST00000248450	AAMP	9.68	3.6203E-02
ENST00000383589	USP14	9.67	4.5227E-02
ENST00000392597	PTPN11	9.67	4.5227E-02
ENST00000526507	FEZ1	9.67	4.5227E-02
ENST00000565726	ZDHHC1	9.67	4.5227E-02
ENST00000591161	NANOS3	9.67	4.5227E-02
ENST00000671888	NANOS3	9.67	4.5227E-02
ENST00000361731	SFT2D1	9.67	3.0708E-02
ENST00000370626	AVPI1	9.66	7.8301E-03
ENST00000537569	TAOK3	9.66	3.0821E-02
ENST00000336180	LIMK1	9.66	1.5393E-02
ENST00000281172	EPS8	9.65	4.5745E-02
ENST00000368409	EFNA4	9.65	4.5745E-02
ENST00000406711	CEBPZOS	9.65	4.5745E-02
ENST00000541360	OGFOD2	9.65	4.5745E-02
ENST00000593892	SELENOW	9.65	4.5745E-02
ENST00000393250	OSBPL8	9.65	3.0668E-02
ENST00000263384	FAM32A	9.65	1.5879E-02
ENST00000548925	BLOC1S1	9.65	3.1931E-02
ENST00000344366	CA12	9.64	4.8582E-02

ENST00000558278	HOMEZ	9.64	3.3955E-02
ENST00000537505	RABEP1	9.64	3.0864E-02
ENST00000397713	AMZ2P1	9.64	3.4032E-02
ENST00000649266	ITM2B	9.64	4.6152E-02
ENST00000462511	TECPR1	9.64	4.6152E-02
ENST00000486645	CMC2	9.64	4.6152E-02
ENST00000575556	PSMB10	9.64	4.6152E-02
ENST00000599519	PEX11G	9.64	4.6152E-02
ENST00000646312	GPI	9.64	4.6152E-02
ENST00000476133	S100A13	9.64	1.4924E-02
ENST00000258526	PLXNC1	9.63	1.7601E-02
ENST00000347770	AZIN1	9.63	3.0321E-02
ENST00000337231	CDR2L	9.63	3.0319E-02
ENST00000444891	HLA-F	9.62	2.9543E-02
ENST00000341491	ATF3	9.62	4.6603E-02
ENST00000375533	BAMBI	9.62	4.6603E-02
ENST00000537952	TAOK3	9.62	4.6603E-02
ENST00000614349	None	9.62	4.6603E-02
ENST00000495837	RPL29P14	9.62	4.6603E-02
ENST00000299705	TMED3	9.62	4.6603E-02
ENST00000453788	HSPA8	9.62	3.3873E-02
ENST00000556605	SLC39A9	9.61	1.9510E-02
ENST00000663135	CALM1	9.61	6.9440E-03
ENST00000402752	RANBP1	9.61	3.1439E-02
ENST00000347934	WAC	9.60	4.9506E-02
ENST00000422974	ZFP91-CNTF	9.60	4.9506E-02
ENST00000491976	SERINC2	9.60	6.2060E-03
ENST00000451583	NBDY	9.60	4.6988E-02
ENST00000503610	GOLPH3	9.60	4.6988E-02
ENST00000641023	PHGDH	9.60	4.6988E-02
ENST00000645471	TPM4	9.60	4.6988E-02
ENST00000518050	CLU	9.60	3.1651E-02
ENST00000265087	STC2	9.59	3.0944E-03
ENST00000367478	TPR	9.59	3.2686E-02
ENST00000536711	MED21	9.59	2.7744E-02
ENST00000530472	PDE4DIP	9.58	3.0852E-02
ENST00000253047	TMEM160	9.58	3.0918E-02
ENST00000295633	FSTL1	9.58	4.7820E-03
ENST00000266987	TARBP2	9.58	4.7419E-02
ENST00000415537	HLA-C	9.58	4.7419E-02
ENST00000524537	NAGK	9.58	4.7419E-02
ENST00000528385	L3MBTL3	9.58	4.7419E-02
ENST00000544741	None	9.58	4.7419E-02
ENST00000367334	TMEM9	9.58	4.7419E-02
ENST00000610709	None	9.57	1.8074E-02

ENST00000530004	TTC39A	9.57	3.1830E-02
ENST00000467531	RPS10	9.57	1.1333E-02
ENST00000273920	ENOPH1	9.56	4.7879E-02
ENST00000464468	GET4	9.56	4.7879E-02
ENST00000560816	ETFA	9.56	4.7879E-02
ENST00000570856	GABARAP	9.56	4.7879E-02
ENST00000383794	CCDC174	9.56	3.1165E-02
ENST00000371730	EPS15	9.55	1.9162E-02
ENST00000324096	MAP1S	9.55	3.1233E-02
ENST00000488803	RPS2P5	9.55	2.4016E-02
ENST00000646664	PS1TP5BP1	9.55	2.5863E-03
ENST00000618010	RAB4A	9.55	3.5865E-02
ENST00000518829	CIBAR1	9.54	4.8185E-02
ENST00000352886	SHMT1	9.54	4.8185E-02
ENST00000472328	IMPDH2	9.54	4.8185E-02
ENST00000526883	SPCS2	9.54	4.8185E-02
ENST00000531601	SCYL1	9.54	4.8185E-02
ENST00000549596	NAP1L1	9.54	4.8185E-02
ENST00000558462	ZNF839	9.54	4.8185E-02
ENST00000437004	PPP1R12A	9.54	4.8185E-02
ENST00000531943	ZNF143	9.54	4.8185E-02
ENST00000561251	ZNF839	9.54	4.8185E-02
ENST00000451270	ANXA2	9.54	6.9348E-03
ENST00000557589	TIMELESS	9.53	3.5683E-02
ENST00000263202	ufd1	9.53	1.6781E-02
ENST00000381401	SLC25A6	9.53	3.6395E-03
ENST00000222266	PSENEN	9.52	2.2313E-02
ENST00000427183	HDLBP	9.52	1.5671E-02
ENST00000328392	SKP1	9.52	3.3085E-02
ENST00000528164	NDUFC2	9.52	3.1738E-02
ENST00000292330	PPP1R35	9.52	4.8684E-02
ENST00000393150	CASP4	9.52	4.8684E-02
ENST00000439980	PIN4	9.52	4.8684E-02
ENST00000470095	HPS1	9.52	4.8684E-02
ENST00000596873	RPS11	9.52	4.8684E-02
ENST00000489852	SDHAF3	9.52	4.8684E-02
ENST00000216442	ATP6V1D	9.52	1.9475E-02
ENST00000395938	PMP22	9.52	3.1774E-02
ENST00000279387	PPP4C	9.52	3.7711E-02
ENST00000439383	PSMD2	9.51	1.8396E-02
ENST00000634374	EFEMP1	9.51	2.3701E-02
ENST00000437418	FAM210B	9.51	3.2686E-02
ENST00000519872	ERLIN2	9.51	3.3590E-02
ENST00000465122	PDCD6IP	9.51	3.2877E-02
ENST00000539539	CCAR1	9.51	2.8926E-02

ENST00000646101	ARPC1B	9.51	3.7988E-03
ENST00000481997	ARPC1B	9.51	2.8458E-03
ENST00000530054	NDUFC2-KCTD14	9.50	3.3027E-02
ENST00000597274	NAPA	9.50	4.9142E-02
ENST00000620199	TMEM191B	9.50	4.9142E-02
ENST00000652401	TYMP	9.50	4.9142E-02
ENST00000535425	SCO2	9.50	4.9142E-02
ENST00000564661	METRN	9.50	3.3117E-02
ENST00000479630	MAP3K7	9.49	3.6758E-02
ENST00000524606	PSMA1	9.49	3.6067E-02
ENST00000268607	MAP1LC3B	9.49	3.8447E-02
ENST00000310837	EIF1	9.48	1.7115E-02
ENST00000318190	TPRKB	9.48	4.9525E-02
ENST00000417312	BNIP2	9.48	4.9525E-02
ENST00000533642	IMMP1L	9.48	4.9525E-02
ENST00000562523	NIP7	9.48	4.9525E-02
ENST00000580090	CENPX	9.48	4.9525E-02
ENST00000635047	SEPTIN7	9.48	4.9525E-02
ENST00000204566	SPG21	9.48	4.9525E-02
ENST00000588354	ZNF585A	9.48	3.6676E-02
ENST00000272102	ARF1	9.47	4.1792E-03
ENST00000470422	RRBP1	9.47	3.3590E-02
ENST00000503026	CCT5	9.47	3.1571E-02
ENST00000470336	ABI3BP	9.47	2.0819E-02
ENST00000463851	GLUL	9.47	3.2838E-02
ENST00000379863	NRBP1	9.46	3.7341E-02
ENST00000482560	KMT2E	9.46	3.3324E-02
ENST00000315717	ARPC2	9.46	2.8453E-03
ENST00000376946	DNAJC1	9.46	4.9960E-02
ENST00000452171	ACAA1	9.46	4.9960E-02
ENST00000459834	PSPH	9.46	4.9960E-02
ENST00000475893	TBRG4	9.46	4.9960E-02
ENST00000486134	PLD3	9.46	4.9960E-02
ENST00000520535	ZNF395	9.46	4.9960E-02
ENST00000533571	MTCH2	9.46	4.9960E-02
ENST00000636293	UROD	9.46	4.9960E-02
ENST00000643386	None	9.46	4.9960E-02
ENST00000338008	ZFYVE16	9.46	4.9960E-02
ENST00000620338	ACD	9.46	3.3590E-02
ENST00000484253	BIN1	9.45	3.7442E-02
ENST00000402886	TEAD3	9.45	3.5812E-02
ENST00000582401	TXNIP	9.45	1.8273E-02
ENST00000272748	LNPK	9.44	2.3798E-02
ENST00000552297	MYL6	9.44	3.4120E-02
ENST00000394030	CBLB	9.44	3.7752E-02

ENST00000335181	PKM	9.43	3.1112E-03
ENST00000303204	PRELID1	9.43	3.5976E-02
ENST00000435275	RPS24	9.42	2.5332E-02
ENST00000554366	COX16	9.42	3.2589E-02
ENST00000529725	SIPA1	9.42	3.5359E-02
ENST00000267328	RAB20	9.41	3.6859E-02
ENST00000455009	ARPC1B	9.41	3.9622E-02
ENST00000541722	GLRB	9.40	3.4552E-02
ENST00000586925	FXYD5	9.40	1.7517E-02
ENST00000367843	DCAF6	9.38	3.8783E-02
ENST00000327374	TANGO2	9.38	3.3955E-02
ENST00000477994	CUEDC2	9.38	3.6032E-02
ENST00000592940	GPX4	9.38	3.0877E-02
ENST00000257663	TMEM60	9.37	3.4019E-02
ENST00000397113	FGFR1	9.37	3.4059E-02
ENST00000548022	ZCRB1	9.37	1.2587E-02
ENST00000260443	C15orf15	9.36	2.3006E-02
ENST00000599310	SMIM7	9.36	3.5152E-02
ENST00000392710	PRKAR1A	9.36	4.1646E-02
ENST00000359755	AKAP12	9.35	3.6371E-02
ENST00000250489	PIP4P1	9.35	8.0639E-03
ENST00000370982	GNG12	9.35	2.7047E-03
ENST00000610189	GPN1	9.35	3.5645E-02
ENST00000323666	METAP2	9.34	3.4946E-02
ENST00000478050	SF3B6	9.34	3.5754E-02
ENST00000584056	RPL23	9.34	3.0263E-02
ENST00000343150	CTSL	9.33	1.5451E-02
ENST00000375400	SVIL	9.33	1.6989E-02
ENST00000336057	HDAC5	9.32	3.4986E-02
ENST00000654051	None	9.32	3.5929E-02
ENST00000265462	PRDX5	9.32	1.2252E-02
ENST00000616058	SUCO	9.32	3.6068E-02
ENST00000548547	IGFBP6	9.32	1.3761E-02
ENST00000441080	PFDN4	9.30	2.3565E-02
ENST00000419016	CHMP5	9.30	3.5638E-02
ENST00000419938	ZEB2	9.30	1.5265E-02
ENST00000376112	ID1	9.30	3.9970E-02
ENST00000522433	KHDRBS3	9.29	3.6676E-02
ENST00000514924	HEL-117	9.29	3.6859E-02
ENST00000657090	GNAS	9.29	1.7101E-02
ENST00000309032	BAD	9.29	4.0219E-02
ENST00000244520	SNRPC	9.29	2.3482E-02
ENST00000418310	LIMK1	9.28	3.7754E-02
ENST00000460284	XRCC5	9.28	3.8917E-02
ENST00000396705	TPI1	9.28	4.3686E-03

ENST00000585132	NCOA4	9.28	2.3121E-02
ENST00000369603	SMNDC1	9.27	9.1793E-03
ENST00000253814	NDFIP1	9.26	2.4221E-02
ENST00000617516	DUSP14	9.26	1.4353E-02
ENST00000409716	TPRKB	9.26	2.1912E-02
ENST00000396194	GEM	9.25	2.1502E-02
ENST00000252603	PGLS	9.25	3.6372E-02
ENST00000328089	SPSB1	9.25	2.2272E-02
ENST00000328965	OAF	9.24	2.3434E-02
ENST00000463214	RBSN	9.24	3.7560E-02
ENST00000521641	CIBAR1	9.24	3.7560E-02
ENST00000265062	RAB7A	9.24	2.3482E-02
ENST00000561428	KIAA0256	9.23	3.6054E-02
ENST00000459785	CASTOR1	9.23	3.8790E-02
ENST00000552783	LIMA1	9.23	3.9304E-02
ENST00000394580	RPL36	9.23	1.4700E-02
ENST00000438270	CYB5R3	9.22	3.8059E-02
ENST00000525212	NDUFS3	9.22	2.8800E-02
ENST00000644568	None	9.22	2.8800E-02
ENST00000422708	DARS1	9.22	3.7282E-02
ENST00000265729	SRI	9.22	2.9409E-02
ENST00000319006	TALDO1	9.21	1.3293E-02
ENST00000546651	C12orf76	9.20	3.8159E-02
ENST00000460192	PARK7	9.20	3.9418E-02
ENST00000468249	ODF3B	9.20	2.9234E-02
ENST00000433482	DAXX	9.19	3.7352E-02
ENST00000253778	GFPT2	9.19	3.7752E-02
ENST00000558562	TMED3	9.18	2.3581E-02
ENST00000329138	HGS	9.18	3.7631E-02
ENST00000427980	ATXN1L	9.18	2.7313E-02
ENST00000303251	RAB24	9.18	2.2468E-02
ENST00000474342	SLTM	9.18	3.0796E-02
ENST00000591919	UBXN6	9.17	3.1916E-02
ENST00000569545	ALDOA	9.17	4.0415E-02
ENST00000517391	CPNE3	9.17	3.9970E-02
ENST00000567983	DCTPP1	9.16	4.3011E-02
ENST00000444129	RECQL	9.16	3.0042E-02
ENST00000469568	MIEN1	9.16	3.8059E-02
ENST00000592764	RNMT	9.15	4.2464E-02
ENST00000358460	FAM104B	9.14	4.2862E-02
ENST00000370322	NDUFB8	9.14	1.4522E-02
ENST00000377575	HMGN4	9.14	3.9622E-02
ENST00000228938	MGP	9.14	4.3320E-02
ENST00000320334	OLFML3	9.14	4.8340E-02
ENST00000467792	PSMD1	9.13	1.5799E-02

ENST00000376630	HLA-E	9.13	1.4916E-02
ENST00000596358	NOSIP	9.13	2.4829E-02
ENST00000495146	RAB10	9.12	2.6251E-02
ENST00000638207	FLII	9.12	4.0960E-02
ENST00000327031	FLII	9.12	4.0960E-02
ENST00000253193	LRP3	9.12	3.8760E-02
ENST00000532829	FTH1	9.12	3.0929E-02
ENST00000479067	MIER1	9.11	4.3872E-02
ENST00000667629	EGLN1	9.11	4.3392E-02
ENST00000429120	LY6E	9.10	4.1063E-02
ENST00000632424	None	9.10	4.1063E-02
ENST00000427521	MEST	9.10	2.5750E-02
ENST00000566906	MAZ	9.10	4.0960E-02
ENST00000371897	SLC2A6	9.09	4.0219E-02
ENST00000625425	None	9.09	4.0219E-02
ENST00000517625	SKP1	9.09	3.9417E-02
ENST00000619818	CWC25	9.09	3.9754E-02
ENST00000633741	None	9.09	3.9754E-02
ENST00000411938	LSM8	9.08	3.4391E-02
ENST00000356415	RAP1A	9.08	2.1150E-02
ENST00000477919	METTL21A	9.06	3.2914E-02
ENST00000362012	PTGS1	9.06	4.0219E-02
ENST00000367142	NUCKS1	9.05	7.6720E-03
ENST00000650521	None	9.05	4.1102E-02
ENST00000639739	LOX	9.05	4.5404E-02
ENST00000486085	RTN4	9.04	1.4299E-02
ENST00000263864	VAMP8	9.02	4.3070E-02
ENST00000322927	ZNF335	9.02	4.1678E-02
ENST00000372634	BEX3	9.02	2.6803E-02
ENST00000263238	ACTR3	9.02	2.8953E-02
ENST00000476371	MRPS14	9.02	4.3070E-02
ENST00000268758	PIGS	9.01	3.2589E-02
ENST00000368706	S100A16	9.01	1.6435E-02
ENST00000449723	DAZAP2	9.01	4.1063E-02
ENST00000456179	FUNDC2	9.01	1.7300E-02
ENST00000284984	ADAMTS1	9.00	2.9029E-02
ENST00000447219	CAPG	8.99	4.1611E-02
ENST00000536012	CCAR1	8.99	3.3282E-02
ENST00000578386	SMURF2	8.99	3.8059E-02
ENST00000258341	LAMC1	8.98	7.5136E-03
ENST00000614837	ATP5F1D	8.98	4.3995E-02
ENST00000298316	ARF6	8.98	2.8727E-02
ENST00000532972	SPCS2	8.97	4.1691E-02
ENST00000570917	TXNDC11	8.97	2.8585E-02
ENST00000428119	RHBDD2	8.97	4.4881E-02

ENST00000437736	PSMD4	8.96	4.3005E-02
ENST00000299424	TAF10	8.96	4.4527E-02
ENST00000460138	TXNDC5	8.95	4.6916E-02
ENST00000330398	ATP6V0C	8.95	4.8779E-02
ENST00000534082	CD44	8.95	4.4443E-02
ENST00000372210	UCK1	8.95	4.7849E-02
ENST00000232125	FAM162A	8.95	2.9566E-02
ENST00000239223	DUSP1	8.95	2.8090E-02
ENST00000601869	STRN4	8.94	4.4759E-02
ENST00000266529	ZCRB1	8.94	1.2093E-02
ENST00000303577	PCBP1	8.94	6.3480E-03
ENST00000368413	ADAM15	8.94	4.8118E-02
ENST00000438323	IFI35	8.94	4.3276E-02
ENST00000360270	MSN	8.93	1.2046E-02
ENST00000616066	None	8.93	1.7324E-02
ENST00000264230	NOA1	8.93	4.3774E-02
ENST00000588732	GLYR1	8.93	1.4358E-02
ENST00000269321	ARHGDIA	8.93	4.6810E-02
ENST00000580793	RFNG	8.92	4.8251E-02
ENST00000506908	HINT1	8.92	4.4128E-02
ENST00000296511	ANXA5	8.92	3.2682E-02
ENST00000256496	ARL8B	8.92	4.4204E-02
ENST00000261401	CORO1C	8.91	1.6603E-02
ENST00000471897	NCOA6	8.91	4.4204E-02
ENST00000394884	CDC23	8.91	4.2928E-02
ENST00000375224	UBR4	8.91	3.0197E-02
ENST00000637937	CTSD	8.91	3.0075E-02
ENST00000236051	EBNA1BP2	8.91	4.8893E-02
ENST00000648836	None	8.90	4.4204E-02
ENST00000461539	C9orf78	8.90	4.4443E-02
ENST00000357348	RTF2	8.90	1.2806E-02
ENST00000394231	MIEN1	8.90	4.4773E-02
ENST00000553660	CNIH1	8.89	4.4443E-02
ENST00000468748	EIF2B5	8.89	4.4488E-02
ENST00000380051	RPP40	8.88	4.4670E-02
ENST00000590466	UBXN6	8.88	3.7630E-02
ENST00000351450	PPA2	8.87	4.9309E-02
ENST00000399443	OSBPL1A	8.86	3.1301E-02
ENST00000290354	HEL-S-25	8.86	4.9309E-02
ENST00000467415	TMEM14C	8.86	3.2181E-02
ENST00000646203	None	8.86	3.2181E-02
ENST00000462332	GTPBP1	8.86	3.6201E-02
ENST00000634130	RBM8A	8.85	4.6988E-02
ENST00000373358	PBDC1	8.85	3.9436E-02
ENST00000351293	CAMK2G	8.84	4.7419E-02

ENST00000371873	CMPK1	8.84	6.2276E-03
ENST00000632401	None	8.83	3.7114E-02
ENST00000645831	FOXC1	8.83	2.9809E-02
ENST00000296255	RPN1	8.83	2.0674E-02
ENST00000532786	C11orf98	8.82	4.7653E-02
ENST00000530876	BSDC1	8.82	4.7653E-02
ENST00000434677	RPL3P2	8.82	4.4773E-02
ENST00000436473	RPL10	8.81	3.0946E-02
ENST00000550786	WASHC4	8.81	4.6433E-02
ENST00000421093	ZNF219	8.81	2.9118E-02
ENST00000394600	MTIF2	8.81	4.7975E-02
ENST00000614345	None	8.81	4.7975E-02
ENST00000447266	SENP6	8.80	4.6709E-02
ENST00000545882	CNN3	8.80	1.4661E-02
ENST00000243040	PFDN5	8.80	3.7754E-02
ENST00000226798	FRG1	8.79	4.8185E-02
ENST00000636590	FRG1	8.79	4.8185E-02
ENST00000637998	FRG1	8.79	4.8185E-02
ENST00000371419	PFDN4	8.79	4.8185E-02
ENST00000488133	RPL13P6	8.79	4.8185E-02
ENST00000599551	UBA52	8.79	2.1814E-02
ENST00000480213	ILF2	8.79	4.6122E-02
ENST00000543117	TAX1BP1	8.78	4.5752E-02
ENST00000263754	KAT2B	8.78	4.6472E-02
ENST00000219070	MMP2	8.77	8.2194E-03
ENST00000489417	SND1	8.77	4.6152E-02
ENST00000504271	SPATA20	8.77	3.1738E-02
ENST00000621073	MED16	8.77	4.8893E-02
ENST00000564811	COX5A	8.76	4.6858E-02
ENST00000346162	SRPK1	8.76	4.6810E-02
ENST00000523094	CLINT1	8.75	4.6916E-02
ENST00000463402	TECPR1	8.75	3.1301E-02
ENST00000358377	DCAF4	8.75	4.7879E-02
ENST00000336139	ZNF202	8.75	4.7260E-02
ENST00000570308	MMP2	8.75	1.4353E-02
ENST00000649906	SLC35B1	8.75	2.0797E-02
ENST00000555944	NSFL1C	8.74	3.1738E-02
ENST00000492092	SELENOO	8.74	4.8044E-02
ENST00000646407	CD9	8.74	4.6858E-02
ENST00000401813	FAAP20	8.74	4.9525E-02
ENST00000613633	DDX52	8.74	4.9525E-02
ENST00000632030	None	8.74	4.9525E-02
ENST00000465788	SRM	8.72	4.9868E-02
ENST00000564374	RPGRIP1L	8.72	4.9868E-02
ENST00000389532	CAMSAP1	8.72	3.1170E-02

ENST00000588674	SBSN	8.71	4.7453E-02
ENST00000611653	GPX4	8.71	6.4508E-03
ENST00000392217	FXYD5	8.70	4.9096E-02
ENST00000397604	RNH1	8.70	1.8884E-02
ENST00000632954	None	8.70	1.8884E-02
ENST00000545751	FMN2	8.70	4.8091E-02
ENST00000564314	RBBP6	8.70	4.7728E-02
ENST00000370355	SCD	8.70	4.6255E-03
ENST00000622875	EBF1	8.69	2.1392E-02
ENST00000324871	METTL1	8.69	4.8118E-02
ENST00000315731	RPL7A	8.69	1.8593E-02
ENST00000627260	None	8.69	1.8593E-02
ENST00000550671	RPL18	8.69	4.8046E-02
ENST00000440400	ACTN4	8.68	1.9591E-02
ENST00000635719	None	8.68	1.9591E-02
ENST00000308162	CFL1	8.68	7.9805E-03
ENST00000434372	FAU	8.68	2.4596E-02
ENST00000330233	CRIP1	8.68	4.9678E-02
ENST00000346916	BSG	8.67	3.2139E-02
ENST00000510098	CAST	8.67	4.8340E-02
ENST00000248114	GFER	8.67	4.9630E-02
ENST00000327435	ADI1	8.67	1.1277E-02
ENST00000461096	TAF13	8.66	3.2056E-02
ENST00000163416	GOLGA5	8.66	4.8498E-02
ENST00000546989	RPLP0	8.66	4.0828E-02
ENST00000366510	SCCPDH	8.66	2.8802E-02
ENST00000347941	PRDX5	8.65	2.3066E-02
ENST00000523636	PABPC1	8.65	4.8693E-02
ENST00000474694	DDX18	8.64	2.2994E-02
ENST00000344774	FAM166A	8.63	4.6433E-02
ENST00000497142	HYPK	8.63	4.6433E-02
ENST00000522572	VDAC3	8.63	4.6527E-02
ENST00000592012	MBD3	8.63	4.9810E-02
ENST00000459859	RPL3	8.63	4.9309E-02
ENST00000395126	RASSF1	8.62	3.2742E-02
ENST00000370179	PPDPF	8.62	1.5020E-02
ENST00000518950	MRNIP	8.62	4.2515E-02
ENST00000639158	None	8.62	4.2515E-02
ENST00000594862	SHKBP1	8.60	4.9859E-02
ENST00000354171	GPX4	8.59	2.6476E-02
ENST00000490936	FLNA	8.59	1.3413E-02
ENST00000480266	ENG	8.59	3.3297E-02
ENST00000216727	PABPN1	8.59	3.3620E-02
ENST00000468830	MKLN1	8.59	1.5122E-02
ENST00000650687	CRYAB	8.57	7.4459E-03

ENST00000228438	CLEC2B	8.57	2.2252E-02
ENST00000237500	MYL12B	8.56	3.4619E-02
ENST00000379965	TRIM22	8.56	3.3793E-02
ENST00000536703	GLIPR1	8.55	3.8447E-02
ENST00000295748	AZI2	8.55	4.0828E-02
ENST00000233468	SF3B6	8.54	4.1678E-02
ENST00000327858	FBLN1	8.54	3.5882E-02
ENST00000471714	ABI3BP	8.53	3.5389E-02
ENST00000589599	H3-3B	8.53	3.7571E-02
ENST00000303127	LMAN2	8.52	1.4158E-02
ENST00000422424	CIAO2B	8.51	2.7529E-02
ENST00000417640	NECAB1	8.51	3.3793E-02
ENST00000451315	NOL7	8.51	3.0981E-02
ENST00000539250	CCAR1	8.50	2.6993E-02
ENST00000380752	SLC7A1	8.50	2.1151E-02
ENST00000271651	CTSK	8.50	1.4955E-02
ENST00000602569	IFITM2	8.49	3.1038E-02
ENST00000425043	DCN	8.48	4.1102E-02
ENST00000444128	NCOA7	8.48	3.4246E-02
ENST00000434693	RBMS3	8.48	2.0578E-02
ENST00000023939	RTF2	8.47	2.3006E-02
ENST00000371646	HSP90AB1	8.45	1.1851E-02
ENST00000246792	RRAS	8.44	3.7029E-02
ENST00000282007	ZC3H13	8.44	3.9595E-02
ENST00000643777	ALDOA	8.44	3.7579E-02
ENST00000457942	CHCHD3	8.43	2.1192E-02
ENST00000335895	BTF3	8.42	3.3191E-02
ENST00000402395	SELENOM	8.42	2.6251E-02
ENST00000400299	SELENOM	8.42	2.9579E-02
ENST00000508783	RPS3A	8.40	4.2464E-02
ENST00000216146	RPL3	8.40	4.7043E-02
ENST00000361813	SMG5	8.39	2.1912E-02
ENST00000595223	KHSRP	8.39	3.1382E-02
ENST00000478503	SH3D19	8.38	3.8224E-02
ENST00000354050	PLTP	8.37	1.6670E-02
ENST00000395246	CDC42SE2	8.37	4.9142E-02
ENST00000379719	IPO7	8.36	2.9490E-02
ENST00000022615	VDAC3	8.35	3.1348E-02
ENST00000614884	DDB2	8.35	3.9890E-02
ENST00000301464	IGFBP6	8.34	1.2345E-02
ENST00000200639	LAMP2	8.33	4.1901E-02
ENST00000482018	FABP3	8.33	2.2192E-02
ENST00000397386	LMCD1	8.33	3.9436E-02
ENST00000432641	SDHAF3	8.32	3.9417E-02
ENST00000570123	COX4I1	8.32	3.1996E-02

ENST00000497275	FABP3	8.31	2.1814E-02
ENST00000217961	STS	8.29	2.5830E-02
ENST00000396024	ANXA2	8.29	1.8224E-02
ENST00000467029	MICOS10	8.28	3.1076E-02
ENST00000344529	PIGC	8.28	4.1063E-02
ENST00000456057	None	8.27	1.1700E-02
ENST00000234111	ODC1	8.27	3.8792E-02
ENST00000544233	WSB2	8.27	2.2359E-02
ENST00000505593	LOX	8.27	3.9436E-02
ENST00000504470	None	8.26	3.1072E-02
ENST00000619951	PSMB3	8.25	2.6301E-02
ENST00000344843	MRPL20	8.24	3.4246E-02
ENST00000263239	DDX18	8.23	1.4859E-02
ENST00000389805	SQSTM1	8.23	1.4353E-02
ENST00000643389	None	8.23	1.4353E-02
ENST00000640444	None	8.23	1.4353E-02
ENST00000271843	JTB	8.23	3.7514E-02
ENST00000261313	HEL-S-34	8.22	3.5978E-02
ENST00000283131	SMARCA5	8.21	3.1913E-02
ENST00000369449	CLIC2	8.20	4.4121E-02
ENST00000492354	SZRD1	8.20	4.1682E-02
ENST00000269228	NPC1	8.20	3.7752E-02
ENST00000370223	LZTS2	8.17	4.4741E-02
ENST00000371222	JUN	8.17	4.1711E-02
ENST00000569696	RPL4	8.15	4.3573E-02
ENST00000443503	CTDSPL	8.15	2.8893E-02
ENST00000246868	SBDS	8.14	4.7344E-02
ENST00000348035	RAC1	8.13	1.7333E-02
ENST00000257770	NT5E	8.12	4.4527E-02
ENST00000279022	MYL9	8.11	2.5334E-02
ENST00000656829	H3-3A	8.11	2.7744E-02
ENST00000646926	RMND1	8.10	4.5916E-02
ENST00000566354	VPS4A	8.09	4.4135E-02
ENST00000433297	HMGCS1	8.09	4.4204E-02
ENST00000302907	RPS9	8.07	1.9019E-02
ENST00000374050	ATP6V1G1	8.07	1.2202E-02
ENST00000546788	NDUFA12	8.06	3.2686E-02
ENST00000467736	RPL13	8.06	1.8392E-02
ENST00000199448	EPDR1	8.03	4.7395E-02
ENST00000526395	SIGIRR	8.03	3.4392E-02
ENST00000267811	TCF12	8.02	2.6122E-02
ENST00000417584	GLUL	8.01	3.8673E-02
ENST00000435381	HSPH1	8.01	3.9418E-02
ENST00000215115	BCL7C	7.99	3.6864E-02
ENST00000622194	NPRL3	7.99	4.6737E-02

ENST00000357037	CAVIN1	7.96	2.1918E-02
ENST00000396210	MGST1	7.96	2.1912E-02
ENST00000298295	DEPP1	7.95	2.9792E-02
ENST00000314675	UBXN11	7.92	2.1151E-02
ENST00000356861	TNPO2	7.91	4.9525E-02
ENST00000380859	AKR1C1	7.90	4.8841E-02
ENST00000402924	BLVRA	7.88	4.8779E-02
ENST00000288466	ZNF618	7.87	2.9300E-02
ENST00000548577	RNASEK	7.87	3.3238E-02
ENST00000397195	PAFAH1B1	7.86	4.3392E-02
ENST00000231061	SPARC	7.85	3.9467E-02
ENST00000498722	LMNA	7.83	3.2181E-02
ENST00000513185	RGMB	7.83	4.9309E-02
ENST00000498309	UQCRQ	7.82	4.4773E-02
ENST00000353704	CREB3	7.81	1.6076E-02
ENST00000373232	HEL-S-66p	7.80	3.9436E-02
ENST00000270792	SH3BGRL3	7.79	2.5732E-02
ENST00000481029	FBH1	7.78	4.0828E-02
ENST00000527987	SIGIRR	7.78	3.5976E-02
ENST00000346473	DDIT3	7.76	1.9742E-02
ENST00000346786	MYL9	7.75	2.8016E-02
ENST00000354332	S100A4	7.73	2.0674E-02
ENST00000369817	RPL10	7.72	2.7063E-02
ENST00000568717	PSMD7	7.72	3.5228E-02
ENST00000242719	RNF11	7.71	4.5793E-02
ENST00000593054	EIF3G	7.69	3.5978E-02
ENST00000535419	PGRMC1	7.67	2.8419E-02
ENST00000468027	PTMA	7.66	4.8185E-02
ENST00000372661	TCEAL9	7.66	3.9622E-02
ENST00000324460	HEL-S-89n	7.65	2.6254E-02
ENST00000427358	FAHD1	7.65	4.0656E-02
ENST00000572355	HCFC1R1	7.65	4.3872E-02
ENST00000357214	SFPQ	7.63	3.9951E-02
ENST00000533498	RPL38	7.58	2.6000E-02
ENST00000398881	TOMM6	7.56	2.6254E-02
ENST00000054666	VAMP3	7.54	4.4204E-02
ENST00000078429	GNA11	7.54	2.3487E-02
ENST00000396373	ETV6	7.53	4.2533E-02
ENST00000610579	FURIN	7.53	4.1691E-02
ENST00000394349	ATP5MC2	7.53	2.5971E-02
ENST00000251413	TUBG1	7.53	4.9868E-02
ENST00000352397	CYB5R3	7.50	3.0263E-02
ENST00000348108	MYL6	7.49	2.9409E-02
ENST00000355801	COMMD6	7.48	4.8190E-02
ENST00000300060	ANPEP	7.47	4.0167E-02

ENST00000344877	ANAPC11	7.46	3.0197E-02
ENST00000225964	COL1A1	7.44	4.7344E-02
ENST00000607373	BNIP2	7.43	2.6367E-02
ENST00000643776	MEA1	7.41	4.9770E-02
ENST00000355693	CHCHD8	7.37	4.9630E-02
ENST00000184266	NDUFB4	7.37	3.0541E-02
ENST00000309311	EEF2	7.35	3.8529E-02
ENST00000253452	COX4I1	7.35	3.3096E-02
ENST00000493224	RPS27	7.34	2.3896E-02
ENST00000367921	DDR2	7.31	2.5732E-02
ENST00000588061	TNRC6C	7.29	3.8387E-02
ENST00000550973	RPL18	7.29	2.9290E-02
ENST00000206423	CCDC80	7.28	3.2450E-02
ENST00000486132	HECTD3	7.28	3.0162E-02
ENST00000481009	S100A4	7.26	3.1070E-02
ENST00000271638	S100A11	7.24	3.3162E-02
ENST00000550697	MYL6	7.21	3.1301E-02
ENST00000339399	ANP32B	7.20	3.9436E-02
ENST00000426013	MORF4L1	7.20	4.6152E-02
ENST00000339647	UBC	7.19	4.9112E-02
ENST00000645147	GPR174	7.18	4.9096E-02
ENST00000552051	HSP90B1	7.17	4.6988E-02
ENST00000334828	PGAM1	7.17	3.2040E-02
ENST00000377612	COMMD6	7.17	4.8942E-02
ENST00000398174	HM13	7.16	3.7352E-02
ENST00000546939	CD63	7.12	3.8059E-02
ENST00000373586	EIF3I	7.11	4.9398E-02
ENST00000353999	EIF4H	7.07	3.0140E-02
ENST00000549920	RPL18	7.05	3.8929E-02
ENST00000317089	DNAJA2	7.05	4.9515E-02
ENST00000497035	RPS8	7.05	4.9142E-02
ENST00000498161	FTH1P8	7.04	3.9750E-02
ENST00000591160	NDUFA11	6.99	3.7361E-02
ENST00000614737	RPS9	6.99	3.6667E-02
ENST00000355968	PPIA	6.99	3.9970E-02
ENST00000301587	ATP5PD	6.96	3.6372E-02
ENST00000547649	MYL6	6.91	3.6114E-02
ENST00000648965	MYL12B	6.87	3.8141E-02
ENST00000421351	PERP	6.87	4.2862E-02
ENST00000378292	TPM2	6.87	4.6433E-02
ENST00000250263	ERI1	6.85	4.8190E-02
ENST00000566564	MMP2	6.85	3.6519E-02
ENST00000569301	HSBP1	6.85	3.3590E-02
ENST00000624069	RPL13A	6.84	3.8077E-02
ENST00000370645	GNG5	6.82	3.8141E-02

ENST00000380636	TMSB4X	6.74	4.4023E-02
ENST00000409248	C4orf48	6.74	4.9083E-02
ENST00000422760	LSM8	6.71	4.2862E-02
ENST00000372692	SET	6.69	4.3793E-02
ENST00000559463	RPL28	6.68	4.4204E-02
ENST00000314133	COX8	6.67	4.6745E-02
ENST00000548898	CD63	6.66	4.7064E-02
ENST00000361899	ATP6	6.64	3.9970E-02
ENST00000610745	CYP1B1	6.64	4.4312E-02
ENST00000361739	MT-CO2	6.61	4.9960E-02
ENST00000509535	RACK1	6.60	4.8185E-02
ENST00000233813	IGFBP5	6.53	4.7653E-02
ENST00000437131	GOLGA4	6.45	4.5227E-02
ENST00000254322	DNAJB1	6.34	4.9525E-02
ENST00000548578	BAZ2A	-7.71	4.7419E-02
ENST00000522356	ZDHHC11B	-7.77	4.6916E-02
ENST00000372371	POLR3A	-7.79	3.8792E-02
ENST00000638782	GABRG2	-8.05	3.8059E-02
ENST00000225831	CCL2	-8.07	4.0491E-02
ENST00000641666	OR7C1	-8.17	4.9525E-02
ENST00000378268	EBPL	-8.19	4.9309E-02
ENST00000434571	MR1	-8.22	4.8185E-02
ENST00000312037	RPS14	-8.33	4.9357E-02
ENST00000632437	NAP1L4	-8.35	4.7395E-02
ENST00000632134	None	-8.39	4.4128E-02
ENST00000518237	IDO1	-8.73	3.6770E-02
ENST00000618876	None	-8.76	3.7352E-02
ENST00000438197	SYTL2	-10.60	7.9550E-03
ENST00000335007	PPP1CC	-21.62	4.7576E-07
