# Supplementary material for: Effects of Hypoxia on RNA Cargo in Extracellular Vesicles from Human Adipose-Derived Stromal/Stem Cells
Source: Int J Mol Sci. 2022 Jul 2;23(13):7384. doi: 10.3390/ijms23137384 (PMC9266528; doi:10.3390/ijms23137384)
Supplement: Supplementary file 1 [file ijms-23-07384-s001.zip › Suppl_Table S3.pdf]

Table S3. List of all 190 miRs detected in nEVs and hEVs.

ID	log2FC (hEVs/nEVs)	padj (hEVs/nEVs)
HSA_PIR_020009/GB/DQ597484/HOMO	4,47	NA
HSA_PIR_019914/GB/DQ597347/HOMO	4,27	4,88E-10
HSA_PIR_013624/GB/DQ588594/HOMO	4,15	1,27E-06
HSA_PIR_020829/GB/DQ598677/HOMO	3,99	3,12E-15
HSA_PIR_018570/GB/DQ595536/HOMO	3,83	4,41E-07
HSA_PIR_020541/GB/DQ598252/HOMO	3,62	0,003228131
HSA_PIR_001312/GB/DQ571813/HOMO	3,58	2,18E-06
HSA-MIR-542-3P	2,91	NA
HSA_PIR_014923/GB/DQ590404/HOMO	2,78	0,00953244
HSA-MIR-193A-5P	2,65	NA
HSA_PIR_004153/GB/DQ575660/HOMO	2,58	0,00953244
HSA_PIR_015026/GB/DQ590548/HOMO	2,49	0,168150406
HSA_PIR_020496/GB/DQ598175/HOMO	2,04	0,3315956
HSA_PIR_016659/GB/DQ592932/HOMO	1,89	0,274316118
HSA_PIR_017716/GB/DQ594453/HOMO	1,84	0,168150406
HSA-MIR-31-3P	1,83	NA
HSA_PIR_016658/GB/DQ592931/HOMO	1,83	0,018534657
HSA-MIR-339-5P	1,77	0,3315956
HSA_PIR_020497/GB/DQ598177/HOMO	1,74	0,288395213
HSA_PIR_001170/GB/DQ571526/HOMO	1,67	NA
HSA-MIR-34C-5P	1,67	NA
HSA-MIR-671-5P	1,67	NA
HSA_PIR_020326/GB/DQ597916/HOMO	1,48	0,054099274
HSA-MIR-181A-5P	1,43	NA
HSA_PIR_000765/GB/DQ570956/HOMO	1,35	0,016437122
HSA-MIR-510-3P	1,31	NA
HSA-MIR-218-5P	1,20	0,567542541
HSA-MIR-185-5P	1,09	NA
HSA-MIR-487B-3P	1,08	NA
HSA-MIR-299-5P	0,95	NA
HSA-MIR-486-5P	0,95	0,73191585
HSA-MIR-122-5P	0,93	0,567542541
HSA-MIR-423-5P	0,90	0,73191585
HSA-MIR-493-5P	0,89	NA
HSA-MIR-140-5P	0,87	0,833107664
HSA-MIR-423-3P	0,82	NA
HSA-MIR-376C-3P	0,82	NA
HSA-MIR-29A-3P	0,80	0,3315956
HSA-MIR-125B-1-3P	0,78	0,73191585
HSA-MIR-99A-5P	0,76	0,833792261
HSA-MIR-503-5P	0,72	NA
HSA-MIR-432-5P	0,70	0,791355633
HSA-MIR-323A-3P	0,66	NA
HSA-MIR-145-5P	0,64	0,841018228
HSA-MIR-103B	0,63	NA
HSA-MIR-493-3P	0,63	NA

HSA-MIR-134-5P	0,62	0,861030031
HSA-MIR-532-5P	0,60	NA
HSA-MIR-106B-3P	0,57	NA
HSA-MIR-196B-5P	0,55	0,833107664
HSA-MIR-221-3P	0,54	0,734337669
HSA-MIR-574-5P	0,51	NA
HSA-MIR-6779-5P	0,44	NA
HSA-MIR-28-3P	0,41	0,910696502
HSA-MIR-30D-5P	0,39	0,848508529
HSA-MIR-136-5P	0,39	0,910696502
HSA-MIR-25-3P	0,39	0,867715534
HSA-MIR-6865-5P	0,38	NA
HSA-MIR-34A-5P	0,36	0,867715534
HSA-MIR-7-5P	0,36	NA
HSA-MIR-146B-5P	0,34	NA
HSA-MIR-24-3P	0,34	0,861030031
HSA-MIR-10A-3P	0,31	NA
HSA-MIR-379-5P	0,26	NA
HSA-MIR-425-5P	0,24	NA
HSA-MIR-127-3P	0,24	0,910696502
HSA-MIR-155-5P	0,22	0,912065894
HSA-MIR-137	0,22	NA
HSA-MIR-140-3P	0,22	0,912065894
HSA-MIR-151A-3P	0,21	0,910696502
HSA-MIR-6789-5P	0,21	0,910696502
HSA-MIR-100-5P	0,21	0,910696502
HSA-MIR-152-3P	0,20	0,910696502
HSA-MIR-4784	0,15	NA
HSA-MIR-31-5P	0,12	0,952402237
HSA-MIR-424-5P	0,10	NA
HSA-MIR-340-5P	0,08	NA
HSA-MIR-20A-5P	0,06	NA
HSA-LET-7D-5P	0,06	0,964075407
HSA-MIR-138-5P	0,06	NA
HSA-MIR-361-3P	0,03	NA
HSA-MIR-494-3P	0,02	NA
HSA-MIR-450B-5P	-0,02	NA
HSA-MIR-497-5P	-0,03	NA
HSA-MIR-126-3P	-0,07	NA
HSA-MIR-224-5P	-0,07	0,952402237
HSA-MIR-30C-5P	-0,09	0,952402237
HSA-MIR-30A-3P	-0,10	NA
HSA-MIR-342-3P	-0,11	0,952402237
HSA-MIR-6781-3P	-0,12	NA
HSA-MIR-29C-3P	-0,12	0,952402237
HSA-MIR-195-5P	-0,13	NA
HSA-MIR-98-5P	-0,19	0,9415499
HSA-MIR-148A-3P	-0,19	0,910696502
HSA-MIR-214-3P	-0,23	0,941261558

HSA-MIR-431-5P	-0,24	NA
HSA-MIR-382-5P	-0,24	0,910696502
HSA-MIR-148B-3P	-0,24	NA
HSA-MIR-381-3P	-0,27	0,910696502
HSA-MIR-92B-3P	-0,28	NA
HSA-MIR-369-3P	-0,28	NA
HSA-MIR-22-3P	-0,30	0,910696502
HSA-MIR-337-5P	-0,31	NA
HSA-MIR-196A-5P	-0,31	0,952402237
HSA-MIR-23A-3P	-0,33	0,861030031
HSA-MIR-574-3P	-0,35	0,910696502
HSA-MIR-191-5P	-0,35	0,864081516
HSA-LET-7G-5P	-0,37	0,853643649
HSA-LET-7B-5P	-0,38	0,734337669
HSA-MIR-27A-3P	-0,44	0,833107664
HSA-MIR-361-5P	-0,46	0,910696502
HSA-MIR-92A-3P	-0,46	0,853643649
HSA-MIR-4440	-0,47	0,910696502
HSA-MIR-186-5P	-0,48	0,853643649
HSA-MIR-16-2-3P	-0,50	NA
HSA-MIR-452-5P	-0,50	NA
HSA-MIR-10A-5P	-0,51	0,833107664
HSA-MIR-199B-3P	-0,54	0,734337669
HSA-MIR-128-3P	-0,55	NA
HSA-MIR-22-5P	-0,55	NA
HSA-LET-7C-5P	-0,56	0,582085167
HSA-MIR-197-3P	-0,57	0,867715534
HSA_PIR_008114/GB/DQ581033/HOMO	-0,57	0,853643649
HSA-MIR-409-3P	-0,66	0,853643649
HSA-MIR-103A-3P	-0,67	0,833107664
HSA-MIR-615-3P	-0,67	NA
HSA-MIR-222-3P	-0,69	0,734337669
HSA-MIR-3135B	-0,73	NA
HSA-MIR-27B-3P	-0,76	0,567542541
HSA-MIR-1307-3P	-0,79	NA
HSA-MIR-199A-3P	-0,81	0,281990907
HSA-MIR-370-3P	-0,83	NA
HSA-MIR-143-3P	-0,83	0,32452441
HSA-MIR-335-3P	-0,85	NA
HSA-MIR-30A-5P	-0,86	0,499049902
HSA-MIR-4532	-0,86	NA
HSA-MIR-454-3P	-0,90	NA
HSA-MIR-30E-5P	-0,92	0,73191585
HSA-MIR-199B-5P	-0,99	0,567542541
HSA-MIR-21-5P	-1,01	0,168150406
HSA-MIR-374B-5P	-1,02	NA
HSA-MIR-99B-5P	-1,02	0,567542541
HSA-MIR-130A-3P	-1,04	NA
HSA-MIR-320A	-1,07	0,734337669

HSA-MIR-16-5P	-1,07	0,199922617
HSA_PIR_008113/GB/DQ581032/HOMO	-1,07	0,380127572
HSA-LET-7A-3P	-1,08	NA
HSA-MIR-4324	-1,11	NA
HSA-MIR-369-5P	-1,12	NA
HSA-MIR-199A-5P	-1,13	0,346900241
HSA-MIR-181B-5P	-1,16	NA
HSA-MIR-411-5P	-1,19	NA
HSA-MIR-132-3P	-1,21	NA
HSA-MIR-26B-5P	-1,22	0,274316118
HSA-MIR-125B-5P	-1,27	0,040712116
HSA-MIR-93-5P	-1,30	0,567542541
HSA-MIR-221-5P	-1,31	NA
HSA_PIR_008112/GB/DQ581031/HOMO	-1,35	0,281990907
HSA-LET-7I-5P	-1,37	0,098197562
HSA-MIR-26A-5P	-1,40	0,065147217
HSA-MIR-29B-3P	-1,55	0,168150406
HSA-MIR-23B-3P	-1,55	0,511141585
HSA-MIR-10B-5P	-1,55	0,337162085
HSA-LET-7A-5P	-1,55	0,031265758
HSA-MIR-660-5P	-1,63	NA
HSA-MIR-654-3P	-1,72	0,567542541
HSA-MIR-30E-3P	-1,76	NA
HSA-MIR-30B-5P	-1,78	NA
HSA-MIR-125A-5P	-1,80	0,014819011
HSA-MIR-143-5P	-1,81	NA
HSA-MIR-1307-5P	-1,85	NA
HSA-MIR-365A-3P	-1,87	NA
HSA-LET-7F-5P	-1,92	0,010317864
HSA-MIR-154-5P	-1,96	NA
HSA-MIR-365B-3P	-1,98	NA
HSA-MIR-151B/151A-5P	-1,99	NA
HSA-MIR-376A-3P	-2,22	NA
HSA-MIR-15A-5P	-2,25	NA
HSA-MIR-335-5P	-2,26	0,3315956
HSA-MIR-101-3P	-2,27	0,246561771
HSA-MIR-19B-3P	-2,34	NA
HSA-LET-7E-5P	-2,34	0,048780307
HSA-MIR-190A-5P	-2,51	NA
HSA-LET-7D-3P	-2,53	NA
HSA-MIR-4301	-2,68	NA
HSA-MIR-15B-5P	-2,69	NA
HSA-LET-7I-3P	-2,83	NA
HSA-MIR-107	-3,08	NA
HSA-MIR-3613-5P	-3,51	NA
HSA-MIR-374A-5P	-3,58	NA
